# Treating Hyperglycemia From *Eryngium caeruleum* M. Bieb: *In*-*vitro* α-Glucosidase, Antioxidant, *in-vivo* Antidiabetic and Molecular Docking-Based Approaches

**DOI:** 10.3389/fchem.2020.558641

**Published:** 2020-11-26

**Authors:** Abdul Sadiq, Umer Rashid, Sadiq Ahmad, Mohammad Zahoor, Mohamed F. AlAjmi, Riaz Ullah, Omar M. Noman, Farhat Ullah, Muhammad Ayaz, Iftikhar Khan, Zia-Ul Islam, Waqar Ali

**Affiliations:** ^1^Department of Pharmacy, Faculty of Biological Sciences, University of Malakand, Chakdara, Pakistan; ^2^Department of Chemistry, COMSATS University Islamabad, Abbottabad Campus, Abbottabad, Pakistan; ^3^Department of Biochemistry, University of Malakand, Chakdara, Pakistan; ^4^Department of Pharmacognosy, College of Pharmacy, King Saud University, Riyadh, Saudi Arabia; ^5^Department of Pharmacy, COMSATS University Islamabad, Abbottabad Campus, Abbottabad, Pakistan; ^6^Department of Biotechnology, Abdul Wali Khan University Mardan, Mardan, Pakistan; ^7^Department of Biotechnology, Faculty of Biological Sciences, University of Malakand, Chakdara, Pakistan

**Keywords:** *Eryngium caeruleum*, h-glucosidase inhibitors, antioxidant, diabetes mellitus, GC- MS, HPLC, molecular docking

## Abstract

Natural-based drugs are believed to be safe, effective and economical. Based on the medicinal importance of the genus Eryngium and unexplored nature of *Eryngium caeruleum*, we have evaluated its antidiabetic and antioxidant potentials. Both *in-vitro* and *in-vivo* assays have been carried out for antidiabetic assays. The antioxidant activity was determined by using different free radicals [i.e., 1,1-diphenyl,2-picrylhydrazyl (DPPH), 2,2-azinobis[3-ethylbenzthiazoline]-6-sulfonic acid (ABTS), and hydrogen peroxide (H_2_O_2_)]. Moreover, different phytoconstituents were identified in the most active solvent fraction by GC-MS analysis. Furthermore, comparative fingerprints of methanolic extract and chloroform fraction were also analyzed via High Performance Liquid Chromatography coupled with Diode Array Detector (HPLC-DAD). The crude methanolic extract of *E. caeruleum* (Ec.Cr) and its sub-fractions [i.e., *n*-hexane (Ec.Hex), chloroform (Ec.Chf), ethyl acetate (Ec.EtAc), and aqueous (Ec.Aq) were employed in this study]. In the α-glucosidase inhibition assay, a concentration-dependent inhibitory response was observed against the enzyme. The most active sample was Ec.Chf which revealed an IC_50_ of 437 μg/ml in comparison to the standard acarbose (IC_50_ 25 μg/ml). The rest of the samples showed moderate inhibition of α-glucosidase. In antioxidant assays, Ec.Chf and Ec.Cr exhibited a considerable scavenging effect against all the free radicals. The IC_50_ values recorded for Ec.Chf were 112, 109, and 150 μg/ml against DPPH, ABTS, and H_2_O_2_ respectively. Based on the *in-vitro* potential of Ec.Chf, this was subjected to the *in-vivo* model experiment. The Ec.Chf lowered the blood glucose level up to 10.3 mmol/L at 500 μg/Kg. The Ec.Chf was also subjected to GC-MS analysis. The GC-MS analysis confirmed the presence of 60 compounds. The identified phytoconstituents consist of some essential compounds previously reported with antidiabetic and antioxidant studies, which include thymol, tocopherol, phytol, nerolidol, (I)-neophytadiene, linolenic acid, and falcarinol. Similarly, the HPLC-DAD chromatograms of Ec.Cr and Ec.Chf exhibited a variety of peaks, which further demonstrates the possibility of important phytochemicals. In a nutshell, we can conclude that *Eryngium caeruleum* is a potential source of bioactive compounds which may be beneficial for the management of ailments like diabetes and free radicals mediated disorders. Molecular docking was performed to explore the possible role of all the identified bioactive compounds in the chloroform fraction of *Eryngium caeruleum* into active sites of the homology model of α-glucosidase.

## Introduction

Diabetes mellitus (DM) is a disorder of carbohydrate, fat and protein metabolism attributed to low production of insulin or mounting resistance to its action leading to persistent hyperglycemia. DM affects about 5% of the population globally and its management exclusive of any side effects is still an issue that confronts the scientific community (Kim et al., 2005) Elevated postprandial blood glucose levels are extensively known as one of the initial disease markers in the prediction of consequent micro-vascular and macro-vascular complications, which ultimately progresses to type 2 DM (Fonseca, 2003). Type 2 DM usually results from defects in insulin secretion and its action. Such insufficiency leads to high concentrations of blood glucose, which sequentially damages many of the body's systems, particularly the blood vessels. Other complications include retinopathy, neuropathy, nephropathy, and angiopathy. One of the strategies for the management of non-insulin dependent diabetes mellitus (NIDDM) is to reduce postprandial hyperglycemia by reducing glucose absorption from the intestinal tract. This strategy is based on the inhibition of key enzymes involved in the metabolism of carbohydrates to simple monosaccharides which are subsequently absorbed (Hussain et al., 2019; Rahim et al., 2019). Among these enzymes α-glucosidase is a crucial enzyme which is widely distributed in animals, plants, and microorganisms (Kimura et al., 2004). This membrane-bound intestinal enzyme is implicated in the catabolism and liberation of α-glucose from the non-reducing side of the complex sugars, making them available for absorption (Nichols et al., 2002). Thus, the administration of enzyme inhibitors will reduce the cleavage of dietary complex carbohydrates to simple monosaccharides, thus leading to a reduction in postprandial glucose levels and suppression of postprandial hyperglycemia (van de Laar, 2008).

Among the currently available synthetic drugs that target the dual metabolic defects of type 2 DM that are impaired insulin secretion and insulin resistance, some of these drugs produce some serious side effects at high doses (Carroll et al., 2003). Therefore, the main goal of anti-diabetic research is to discover anti-hyperglycemic agents that are comparatively safe with fewer side effects. In this regard, research has begun to study traditional medicines and foods for the discovery of new therapeutic drugs against type 2 DM. Traditional medicines including plants and herbal extracts have a long history of use as anti-diabetic agents (Grover et al., 2002). Indeed, a lot of medicinal plants have been reported inhibiting the activity of α-glucosidase (Bnouham et al., 2006). Dietary α-glucosidase inhibitors that act by inhibiting the enzymatic breakdown of starch, soluble carbohydrates, and their derived products have been recognized as a potential natural and safe approach for reduction of hyperglycemia via modulation of meal derived glucose absorption (Bischoff, 1994).

In DM, hyperglycemia set free reactive oxygen species (ROS) called free radicals, which subsequently cause lipid peroxidation, membrane damage and play a vital role in the initiation of secondary complications of kidneys, eyes, blood vessels, nervous system, and DM (Hunt et al., 1988). Numerous studies established that oxidative stress is implicated in the development of insulin resistance via inhibition of insulin signals and adipokines dysregulation (Houstis et al., 2006). Antioxidants have been reported to thwart the destruction of β-cells via inhibition of peroxidation chain reaction and consequently offer protection against the development of DM (Montonen, 2005). The antioxidant can be from both organic and inorganic sources (Bibi et al., 2019; Zafar et al., 2020). However, medicinal plants are enriched with natural antioxidants that can protect β-cell function and stop diabetes mediated ROS formation (Sadiq et al., 2015a; Begum et al., 2019).

*Eryngium caeruleum* belongs to the family Apiaceae. The genus Eryngium comprises of 250 species out of which less than 30 species have been investigated phytochemically to date (Capetanos et al., 2007). *E. caeruleum* is one of the unexplored flora of Eryngium. Several species of this genus have been used traditionally for the treatment of various ailments for instance, edema, inflammatory disorders, sinusitis, snake and scorpion bites, wound healing, urinary tract infections, as antitussive, fertility disorders etc., (Singh et al., 2002).

**Figure 1:**
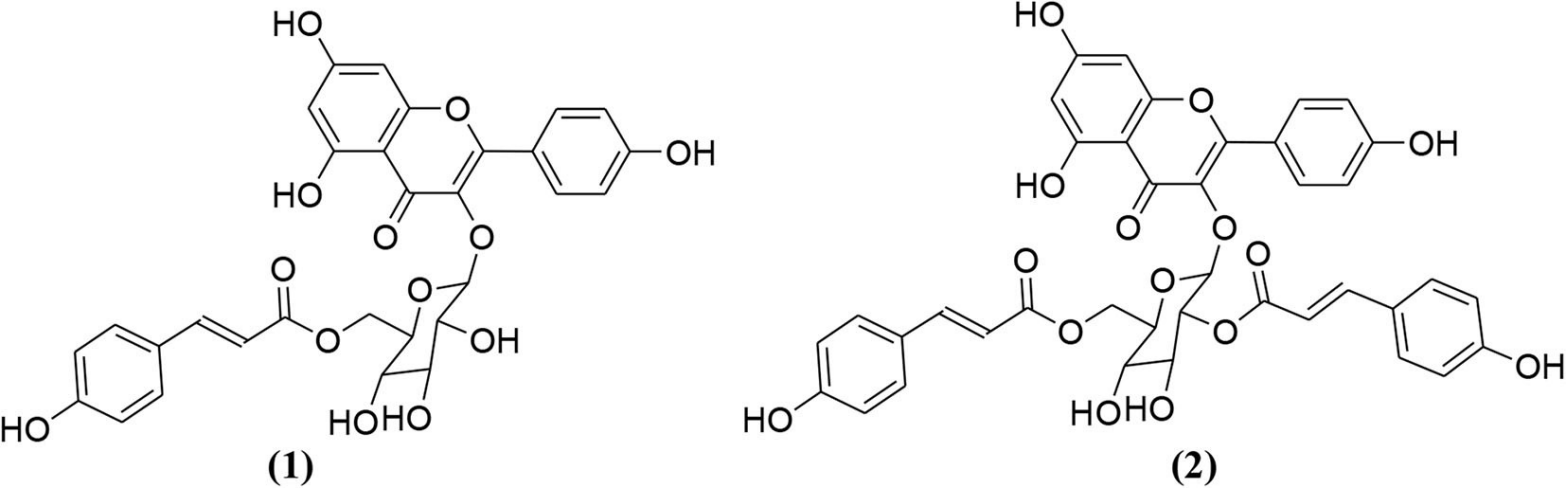
Chemical structures of two isolated phytochemicals from *E. caeruleum*.

Rehman et al. investigated the aerial parts of *Eryngium caeruleum* and isolated two new flavone glycosides (**1** and **2**, Figure 1) (Rehman et al., 2017). These two isolated compounds were evaluated for their potential against α-glucosidase, aldose reductase (ALR1 and ALR2). Compounds were only found active against ALR1 and ALR2.

We previously reported the antimicrobial properties of *E. caeruleum* (Sadiq et al., 2016). As is obvious from the current literature, only the essential oil of this plant has been explored up to some extent with excellent pharmacological activities (Saeedi and Morteza-Semnani, 2008). The plant powder and extract of *E. caeruleum* are still to be explored. Moreover, the isolated chemicals by Rehman et al. were not able to show α- β-glucosidase inhibition and antiglycation potential. Being unexplored and one of the specie of important genus, this investigational study was inevitable. Therefore, the current investigational study is arranged to assay various solvent fractions of *E. caeruleum* for inhibitions of α-glucosidase and free radicals followed by GC-MS analysis of the most potent solvent fraction.

## Methods

### Chemicals and Drugs

The solvents used in the study were of analytical grades which were purchased from Sigma Aldrich via local venders. The solvents used in HPLC analysis were of HPLC grades. The chemicals and drugs used were purchased from the local suppliers of Sigma Aldrich. The chemicals and drugs used were α-glucosidase (9001-42-7), alloxan monohydrate (ALX2244-11-3), Acarbose (56180-94-0), *p*-Nitrophenyl-α-D-glucopyranoside (3767-28-0) 2,2-diphenyl-1-picrylhydrazyl (1898-66-4), 2,2′-Azino-bis(3-ethylbenzothiazoline-6-sulfonic acid) (30931-67-0), Hydrogen peroxide, glibenclamide (10238-21-8), Ascorbic acid (50-81-7).

### Plant Collection and Extraction

The aerial parts of *Eryngium caeruleum* were collected from various areas of the Malakand division and authenticated by Dr. Nasrullah at Department of Botany, University of Malakand. The plant was pressed and kept in the herbarium of University of Malakand, Pakistan under voucher specimen number H.UOM.BG.109. After collection, the plant was cut into small pieces and allowed to dry in shade. After drying, the plant was powdered using a cutter mill and macerated in 80% methanol for two weeks. After soaking, the plant sample was filtered using Whatman filter paper. The filtrates were evaporated under reduced pressure using rotary evaporator at 40°C (Farooq et al., 2019; Sadiq et al., 2019). Semi solid masse of methanolic extract of *E. caerulum* was obtained weighing ~400 g.

### Fractionation

The successive solvent-solvent extraction method was followed for the fractionation of crude methanolic extract (Shah et al., 2014a). In brief, the crude extract of *E. caerulum* weighing 300 g was suspended in 500 ml of distilled water in separating funnel and 500 ml of *n*-hexane was poured into it. It was vigorously shaken and allowed to stand in a separating funnel to be separated into two distinct layers. The upper *n*-hexane layer was collected, and the same procedure was followed until a colorless *n*-hexane layer was obtained. After the collection of *n*-hexane fraction, it was fractionated with other solvents with increasing order of polarity (i.e., chloroform, ethyl acetate and the aqueous fraction). The fractions obtained for *n*-hexane, chloroform, ethyl acetate and aqueous fractions of *E. caerulum* were allowed to get converted into semisolid masses weighing 62 g (20. 66%), 37 g (12.33%), 59 g (19.66%), and 110 g (36.66%) respectively.

### *In-vitro* α-Glucosidase Inhibition Assay

The α-glucosidase inhibitory potentials of solvent extracts from *E. caeruleum* were investigated following the reported protocol (Aslam et al., 2018). The enzyme solution was made by dissolving 0.5 unit/ml in a 0.1 M phosphate buffer (pH 6.9). The final concentration of enzyme solution was 20 μl α-glucosidase (0.5 unit/ml). Whereas, *p*-Nitrophenyl-α-D-glucopyranoside (5 mM) was made in the same buffer (pH 6.9) which acted as substrate for the reaction. Test samples prepared were mixed with the enzyme solution and incubated for 15 min at 37°C. Finally, 20 μl substrate solution was added to the enzyme mixture and was incubated for 15 min at 37°C. The reaction was completed by the addition of 80 μl of 0.2 M sodium carbonate solution. Absorbances were measured at 405 nm using UV spectrophotometer (Thermo electron corporation, USA). The system without α-glucosidase was used as the blank and acarbose was the positive control. Each experiment was conducted in triplicate and percent inhibitions were calculated using the following formula;

$$\begin{array}{l} \%\text{ inhibition}=\frac{\text{Control absorption }-\text{ Sample absorption}}{\text{Control absorption}} \end{array}$$

### Antioxidant Assays

#### DPPH Radical Scavenging Assay

DPPH free radical scavenging ability of test samples were tested according to previously reported procedures (Shah et al., 2015; Zahoor et al., 2018). Samples solutions were prepared ranging from 125 to 1,000 μg/ml and were added to 0.004% methanolic solution of DPPH. Following incubation for 30 min, absorbance values were recorded at 517 nm using UV spectrophotometer (Thermo electron corporation, USA). DPPH scavenging activities were calculated as;

$$\begin{array}{l} \text{DPPH scavenging assay }\left(\%\right)=\frac{A_{0}-A_{1}}{A_{0}}\times 100 \end{array}$$

Where A_0_ characterizes absorbance of the control and A_1_ is the absorbance of test samples. Ascorbic acid was used as a positive control. Where A_0_ characterizes absorbance of control and A_1_ is the absorbance of test samples. All experiments were performed in triplicate and inhibition graphs were made with the help of GraphPad prism program (GraphPAD, San Diego, California, USA).

#### ABTS Free Radical Scavenging Assay

The ABTS free radical scavenging potential of Plant samples were tested following previously published procedure (Zafar et al., 2019). Briefly, solutions of ABTS 7 mM and potassium persulphate (K_2_S_2_O_4_) 2.45 mM were mixed and stored in a dark place at room temperature for about 12–16 h to obtain a dark colored solution. This solution was diluted using Phosphate buffer (0.01 M) pH 7.4 and absorbance value was adjusted to 0.70 at 734 nm. Finally, 300 μl solution of the test sample was added to 3.0 ml of ABTS solution in cuvette and was analyzed using a UV spectrophotometer (Thermo electron corporation, USA) at 734 nm. Reduction in absorbances was determined following 1 min of mixing the solutions and analysis was continued for 6 min. Ascorbic acid was used as a positive control. The assay was repeated in triplicate and percentage inhibition was calculated using the formula;

$$\begin{array}{l} \text{ABTS scavenging assay }\left(\%\right)\\ =\frac{\text{Control absorbance }-\text{ Sample absorbance}}{\text{Control absorbance}}\times 100 \end{array}$$

#### Hydrogen Peroxide Scavenging Assay

Hydrogen peroxide (H_2_O_2_) scavenging capacity of plant samples were evaluated using the previously reported method (Ahmad et al., 2019). In brief, 2 mM hydrogen peroxide solution was prepared in 50 mM phosphate buffer with 7.4 pH. Plants samples (0.1 ml) were taken in test tubes and their volumes were raised to 0.4 ml using 50 mM phosphate buffer solution. H_2_O_2_ (0.6 ml) was added to the tubes and were properly mixed using vertex mixer. Absorbance of each sample was measured at 230 nm against the blank after 10 min. H_2_O_2_ scavenging activity was calculated using the equation;

$$\begin{array}{l} {\text{H}}_{2}{\text{O}}_{2}\text{ scavenging assay }\left(\%\right)=\frac{\left(1\;-\text{ Sample absorbance}\right)}{\text{Control absorbance}}\times 100 \end{array}$$

### Experimental Animals

In the *in-vivo* antidiabetic study, we used Albino mice of either sex with an average weight of up to 30 gm. The animals were kept in an animal house as per the standard procedure of the animals' Bye-Laws and under the approval of Ethical Committee, Department of Pharmacy, Faculty of Biological Sciences, University of Malakand, Pakistan as per the animals Bye-Laws 2008 (Scientific Procedure Issue-I).

### Acute Toxicity

The mice were divided into two groups (i.e., test group and control group, having five animals in each of the group). The samples were administered I.P in a concentration ranges of 250–2,000 mg/kg body weight. After the samples' administrations, the animals were critically observed for 3 days for any abnormal response or mortality.

### Induction of Diabetes

Initially, we administered alloxan monohydrate (10%, 150 mg/kg) intraperitoneally to the experimental animals for the induction of hyperglycemia. Meanwhile, a control group was also maintained by experimental animals which received an intraperitoneal dose of normal saline. After the alloxan monohydrate injection, the animals were sorted out into diabetic and non-diabetic animals by using a glucometer. The animals with high blood glucose level (diabetes) were subjected to further studies as per the previous reported procedure (Hussain et al., 2019).

### *In-vivo* Experiment

In the *in-vivo* experiment, the diabetic mice were divided into groups I-IV (having 5 mice in each). The group I was the normal one given normal saline and having no diabetes. Group II was given Tween 80 solution and was the diabetic group. Group III was given a standard drug for the treatment of diabetes (i.e., on 500 μg/kg body weight of glibenclamide). Group IV was given different concentrations of the potent chloroform fraction (Hussain et al., 2019).

### Gas Chromatography (GC) Analysis

Chloroform fraction was analyzed by means of an Agilent USB-393752 gas chromatograph (Agilent Technologies, Palo Alto, CA, USA) with HHP-5MS 5% phenylmethylsiloxane capillary column (30 m × 0.25 mm × 0.25 μm film thickness; Restek, Bellefonte, PA) equipped with an FID detector. The temperature of the oven was retained at 70°C for 1 min at first, and then raised at the rate of 6°C/min to 180°C for 5 min and to finish at the rate of 5°C/ min to 280°C for 20 min. Injector and detector temperatures were set at 220 and 290°C, correspondingly. Helium was utilized as carrier gas at a flow rate of 1 ml/min (Mahmood et al., 2019).

### Gas Chromatography–Mass Spectrometry (GC/MS) Analysis

GC/MS analysis of the chloroform fraction was processed by means of an Agilent USB-393752 gas chromatograph (Agilent Technologies, Palo Alto, CA, USA) with a HHP-5MS 5% phenylmethylsiloxane capillary column (30 m × 0.25 mm × 0.25 μm film thickness; Restek, Bellefonte, PA) prepared with an Agilent HP-5973 mass selective detector in the electron impact mode (Ionization energy: 70 eV) working under a similar experimental environment as illustrated for GC (Shah et al., 2012).

### Identification of Components

Compounds present in chloroform fraction of *E. caeruleum* were identified by comparison of the retention times of each of them with those of authentic compounds in the literature. Additional identification was performed from the spectral data obtained from the Wiley and NIST libraries and further identifications were completed by comparisons of the fragmentation pattern of the mass spectra with the published data in the literature (Zeb et al., 2017).

### HPLC-DAD Analysis

#### Preparation of Samples for HPLC

For the preparation of samples, 20 ml of methanol and water (1:1 v/v) were mixed with 1 gram of *E. caeruleum* samples. This mixture was then heated on a water bath at 70°C for 1 h. After cooling, the sample was centrifuged at 4,000 rpm for 10 min. Then 2 ml of sample was filtered with Whatman filter paper into HPLC vials.

#### HPLC –DAD Procedure

The determination of various compounds' peaks was carried out by means of an Agilent 1260 infinity High-performance liquid chromatography (HPLC) system, having basic parts like quaternary pump, auto-sampler, degasser and ultraviolet array detector (UVAD). The separation was achieved via Agilent Zorbax Eclipse XDB-C18 column. The gradients system comprises of solvent B (methanol: acetic acid; deionized water, 100: 20: 180, v/v) and solvent C (methanol : acetic acid : deionized water, 900: 20: 80, v/v). The efficient gradient program was started with 100% B at 0 min, 85% B at 5 min, 50% B at 20 min, 30% B at 25 min, and 100% C from 30 to 40 min (Zeb et al., 2017). After 25 min, elution occurred. For the analysis of antioxidants compounds, the ultraviolet array detector (UVAD) was set to 280 nm and the spectra were recorded from 190 to 500 nm.

#### Estimation of IC_50_ Values

For α-glucosidase results, IC_50_ values were calculated from a dose response curve using Microsoft Excel program. Furthermore, in anti-radicals assays including DPPH, ABTS and H_2_O_2_ the IC_50_ were calculated following the same protocol.

#### Statistical Data Analysis

All experiments were performed in triplicate and results were expressed as mean ± S.E.M of percent inhibition values. One-way ANOVA followed by Dunnett's multiple comparison test was applied for the comparison of positive control with the test group using GraphPad prism Software USA. The *P* < 0.05 were considered as statistically significant (Shah et al., 2014b).

#### Docking Studies

Molecular docking studies were made by Molecular-Operating Environment (MOE-2016.08) (Munir et al., 2020). Docking study for α-glucosidase enzyme was performed on our previously reported homology modeled α-glucosidase (Ali et al., 2018). Preparation of ligand's downloaded enzymes such as determination of binding sites, energy minimization and 3-D protonation was performed by previously reported methods (Iftikhar et al., 2018; Ullah et al., 2018; Tanoli et al., 2019). Docking results were interpreted, and their surfaces were analyzed with graphical representation utilizing discovery-studio visualizer (Biovia, 2017).

## Results

### α-Glucosidase Inhibition Assay

In α-glucosidase inhibition assay, Ec. Chf was observed to be the most active fraction. Ec. Chf exhibited 59.57 ± 1.18, 52.67 ± 0.11, 43.86 ± 0.02, and 36.72 ± 0.45% enzyme at concentrations of 1,000, 500, 250, and 125 μg/ml respectively attaining IC_50_ of 734 μg/ml. Likewise, Ec. Cr displayed 55.46 ± 0.44, 39.51 ± 1.11, 33.77 ± 1.14, and 27.72 ± 1.45% inhibitions at the same tested concentrations with an IC_50_ value of 855 μg/ml, respectively. In comparison, the standard drug acarbose showed 76.87 ± 0.06, 73.94 ± 1.92, 67.49 ± 0.22, and 61.53 ± 0.89% inhibitions at concentrations of 1,000, 500, 250, and 125 μg/ml respectively with IC_50_ of 25 μg/ml. Other fractions, including Ec. EtAc, Ec. Hex, and Ec. Aq exhibited dose-dependent enzyme inhibition with IC_50_ of 754, 1,388, and 1,208 μg/ml respectively as shown in Table 1.

**Table 1:** Alpha glucosidase inhibitory potentials of the samples from *Eryngium caeruleum*.

**Samples**	**Concentrations (μg/ml)**	**Percent inhibition**	**IC_**50**_ (μg/ml)**
Ec.Cr	1,000	55.46 ± 0.44^**^	855
	500	39.51 ± 1.11^***^	
	250	33.77 ± 1.14^***^	
	125	27.72 ± 1.45^***^	
Ec.Hex	1,000	46.61 ± 1.73^***^	1,388
	500	39.70 ± 0.03^***^	
	250	32.77 ± 1.14^***^	
	125	26.55 ± 0.15^***^	
Ec.Chf	1,000	59.57 ± 1.18^**^	437
	500	52.67 ± 0.11^***^	
	250	43.86 ± 0.02^***^	
	125	36.72 ± 0.45^***^	
Ec.EtAc	1,000	53.62 ± 0.97^**^	754
	500	47.41 ± 1.73^***^	
	250	41.92 ± 0.19^***^	
	125	35.57 ± 0.23^***^	
Ec.Aq	1,000	48.72 ± 2.77^***^	1,208
	500	42.35 ± 0.12^***^	
	250	37.28 ± 2.28^***^	
	125	30.69 ± 0.14^***^	
Acarbose (Positive control)	1,000	76.87 ± 0.06	25
	500	73.94 ± 1.92	
	250	67.49 ± 0.22	
	125	61.53 ± 0.89	

*All values are taken as Mean ± SEM (n = 3). The P-values <0.05 were considered as statistically significant. Values significantly different in comparison to standard drug (i.e.*,

** P < 0.01 and

*** *P < 0.001)*.

### Antioxidant Assays

The results obtained from the antioxidant activity of *E. caeruleum* using DPPH, ABTS and H_2_O_2_ free radicals assays are summarized in Table 2.

**Table 2:** Antioxidant potential of *Eryngium caeruleum* samples using different free radicals assays.

**Samples**	**Conc (μg/ml)**	**DPPH**		**ABTS**		**H**_****2****_**O**_****2****_	
		**Percent inhibition (mean ± SEM)**	**IC_**50**_ (μg/ml)**	**Percent inhibition (mean ± SEM)**	**IC_**50**_ (μg/ml)**	**Percent inhibition (mean ± SEM)**	**IC_**50**_ (μg/ml)**
Ec.Cr	1,000	73.33 ± 0.60^*^	163	72.30 ± 0.62^*^	175	75.16 ± 0.72^ns^	145
	500	64.16 ± 0.44^***^		64.13 ± 0.46^**^		66.06 ± 0.52^ns^	
	250	52.83 ± 0.72^***^		53.26 ± 0.59^**^		55.10 ± 0.66^ns^	
	125	44.50 ± 0.86^***^		46.16 ± 0.60^**^		46.33 ± 0.52^ns^	
Ec.Hex	1,000	65.16 ± 0.88^***^	405	66.16 ± 0.44^***^	332	66.13 ± 0.46^**^	305
	500	53.50 ± 0.76^***^		56.13 ± 0.46^***^		56.13 ± 0.69^***^	
	250	43.33 ± 0.44^***^		46.10 ± 0.58^***^		47.23 ± 0.61^***^	
	125	35.16 ± 0.92^***^		35.20 ± 0.41^***^		36.03 ± 0.60^***^	
Ec.Chf	1,000	76.50 ± 0.57^ns^	112	75.13 ± 0.69^*^	109	75.46 ± 0.97^ns^	150
	500	67.16 ± 0.60^***^		67.23 ± 0.61^**^		68.16 ± 0.44^ns^	
	250	58.83 ± 0.88^***^		60.56 ± 0.42^ns^		54.06 ± 0.52^ns^	
	125	52.50 ± 0.28^***^		54.06 ± 0.58^ns^		46.10 ± 0.58^ns^	
Ec.EtAc	1,000	70.16 ± 0.72^**^	204	69.10 ± 0.66^***^	200	64.03 ± 0.54^**^	330
	500	61.60 ± 0.49^***^		61.76 ± 0.78^***^		59.10 ± 0.49^***^	
	250	51.70 ± 0.47^***^		51.10 ± 0.58^***^		44.16 ± 0.72^***^	
	125	42.20 ± 0.41^***^		43.16 ± 0.60^***^		38.30 ± 0.56^***^	
Ec.Aq	1,000	57.86 ± 0.69^***^	580	57.20 ± 0.41^***^	590	53.20 ± 0.55^***^	790
	500	48.30 ± 0.40^***^		49.16 ± 0.72^***^		45.20 ± 0.63^***^	
	250	43.43 ± 0.52^***^		43.10 ± 0.58^***^		38.13 ± 0.59^***^	
	125	34.20 ± 0.61^***^		34.16 ± 0.52^***^		32.36 ± 0.77^***^	
Ascorbic acid	1,000	90.66 ± 0.70	10	89.10 ± 0.49	60	85.13 ± 0.46	85
	500	85.46 ± 0.39		81.20 ± 0.41		74.13 ± 0.55	
	250	76.70 ± 0.40		70.36 ± 0.57		65.46 ± 0.73	
	125	69.06 ± 0.58		63.26 ± 0.67		53.36 ± 0.63	

*All values are taken as Mean ± SEM (n = 3), Values significantly different in comparison to standard drug (i.e.*,

* *P < 0.05*,

** *P < 0.01*,

*** *P < 0.001*.

*ns = Values not significantly different in comparison to positive control)*.

### DPPH Free Radicals Scavenging Assay

In DPPH free radicals scavenging assay, Ec.Chf, Ec.Cr, and Ec.EtAc were the potent fractions. Ec.Chf showed 76.50 ± 0.57, 67.16 ± 0.60, 58.83 ± 0.88, and 52.50 ± 0.28% inhibitions of free radicals at concentrations of 1,000, 500, 250, and 125 μg/ml respectively. Similarly, Ec.Cr displayed 73.33 ± 0.60, 64.16 ± 0.44, 52.83 ± 0.72, and 44.50 ± 0.86% inhibitions at concentrations 1,000, 500, 250, and 125 μg/ml, respectively. Furthermore, Ec.EtAc enzyme inhibitions were 70.16 ± 0.72, 61.60 ± 0.49, 51.70 ± 0.47, and 42.20 ± 0.41% at the same tested concentrations. The IC_50_ values for Ec.Chf, Ec.Cr, and Ec.EtAc were 112, 163, and 204 μg/ml, respectively. Standard drug ascorbic acid showed 90.66 ± 0.70, 85.46 ± 0.39, 76.70 ± 0.40, and 69.06 ±0.58% scavenging of free radicals at concentrations 1,000, 500, 250, and 125 μg/ml respectively. The IC_50_ value calculated for ascorbic acid was 10 μg/ml. Ec.Hex and Ec.Aq also displayed a dose dependent response with IC_50_ of 405 and 580 μg/ml, respectively as shown in Table 2.

### ABTS Free Radicals Scavenging Assay

In ABTS anti-radicals assay, Ec.Chf displayed the highest activity causing 75.13 ± 0.69, 67.23 ± 0.61, 60.56 ± 0.42, and 54.06 ± 0.58% scavenging of free radicals at concentrations of 1,000, 500, 250, and 125 μg/ml, respectively. Ec.Cr was equally effective causing 72.30 ± 0.62, 64.13 ± 0.46, 53.26 ± 0.59, and 46.16 ± 0.60 μg/ml respectively at concentrations of 1,000, 500, 250, and 125 μg/ml, respectively. For Ec.Chf and Ec.Cr the IC_50_ values were 109 and 175 μg/ml respectively. Ec.EtAc, Ec.Hex, and Ec.Aq showed 69.10 ± 0.66, 66.16 ± 0.44, and 57.20 ± 0.41 at highest tested concentration attaining IC_50_ values of 200, 332, and 590 μg/ml, respectively. In scavenging of ABTS free radicals, the standard drug ascorbic acid exhibited 89.10 ± 0.49, 81.20 ± 0.41, 70.36 ± 0.57, and 63.26 ± 0.67% inhibitions respectively at the same concentrations with IC_50_ of 60 μg/ml (Table 2).

### H_2_O_2_ Free Radicals Scavenging Assay

In hydrogen peroxide radicals scavenging assay, Ec.Chf and Ec.Cr exhibited the highest radicals scavenging activity. Ec.Chf showed 75.46 ± 0.97, 68.16 ± 0.44, 54.06 ± 0.52, and 46.10 ± 0.58% anti-radicals activity at concentrations of 1,000, 500, 250, and 125 μg/ml, respectively. For Ec.Cr, the observed scavenging efficiency was 75.16 ± 0.72, 66.06 ± 0.52, 55.10 ± 0.66, and 46.33 ± 0.52% at the same concentrations. The IC_50_ values for Ec.Chf, Ec.Cr were 150 and 145 μg/ml respectively. Among other fractions, Ec.Hex caused 66.13 ± 0.46, 56.13 ± 0.69, 47.23 ± 0.61, and 36.03 ± 0.60% inhibitions at concentrations of 1,000, 500, 250, and 125 μg/ml respectively. The ABTS free radicals scavenging efficacy of Ec. EtAc was mediocre with IC_50_ of 330 μg/ml. The aqueous fraction showed the least activity with IC_50_ value of 790 μg/ml. Standard drug ascorbic acid inhibitory activity was 85.13 ± 0.46, 74.13 ± 0.55, 65.46 ± 0.73, and 53.36 ± 0.63% at concentrations of 1,000, 500, 250, and 125 μg/ml respectively with IC_50_ of 85 μg/ml.

### *In*-*vivo* Antidiabetic Studies

Based on the *in-vitro* assays of all fractions of *Eryngium caeruleum*, the chloroform fraction was selected for the *in-vivo* assay as can be seen in Table 3. In acute toxicity studies, the Ec.Chf was found to be safe, causing no mortality or unwanted reactions. The chloroform fraction of *Eryngium caeruleum* was observed to be a potent antidiabetic agent following the *in-vivo* experiments. The Ec.Chf at concentrations of 500, 250, 125, 62.5, and 31.25 μg/kg body weight exhibited a blood glucose level of 10.3, 15.4, 16.8, 17.8, and 19.4 mmol/liter in mice. Obviously, the blood glucose level at 24 h and 500 μg/kg was 10.3 mmol/liter which was almost in comparison to the standard drug value which was 7.9 mmol/liter.

**Table 3:** *In*-*vivo* antidiabetic results of chloroform fraction of *Eryngrium caeruleum*.

**Sample**	**Conc./administration route**	**Glucose level at varying time mmole/L**					
		**0 h**	**2 h**	**4 h**	**6 h**	**8 h**	**24 h**
Group I (Normal saline)	Intraperitoneal	5.15^***^	5.17^***^	5.21^***^	5.22^*^	5.25^*^	5.28^ns^
Group II (Tween 80)	Intraperitoneal	22.4^ns^	22.5^ns^	22.6^*^	22.8 ^**^	22.9^***^	23.1^***^
Group III (Glibenclamide)	500 μg/kg Intraperitoneal	22.8	19.8	16.9	13.6	10.4	7.9
Group IV (Ec.Chf)	Intraperitoneal						
	500 μg/kg	23.5^ns^	21.2^ns^	18.4^ns^	16.1^ns^	13.4^ns^	10.3^ns^
	250 μg/kg	22.9^ns^	21.9^ns^	19.8^ns^	18.2^ns^	16.5^ns^	15.4^ns^
	125 μg/kg	23.6^ns^	22.4^ns^	20.7^ns^	18.6^ns^	17.5^*^	16.8^*^
	62.5 μg/kg	24.0^ns^	22.4^ns^	20.7^ns^	19.6^*^	18.6^*^	17.8^**^
	31.25 μg/kg	22.7^ns^	21.4^ns^	20.8^ns^	20.2^*^	19.8^*^	19.4^**^

*Each value represents mean ± SEM of three independent experimental studies. Values significantly different when compared with compared with positive control (Glibenclamide) at the same time interval (i.e.*,

*** *P < 0.001*,

** *P < 0.01*,

* *P, 0.05*,

*ns = values not significantly different as compared to standard drug)*.

### Identification of phytoconstituents (GC-MS Analysis)

**Figure 2:**
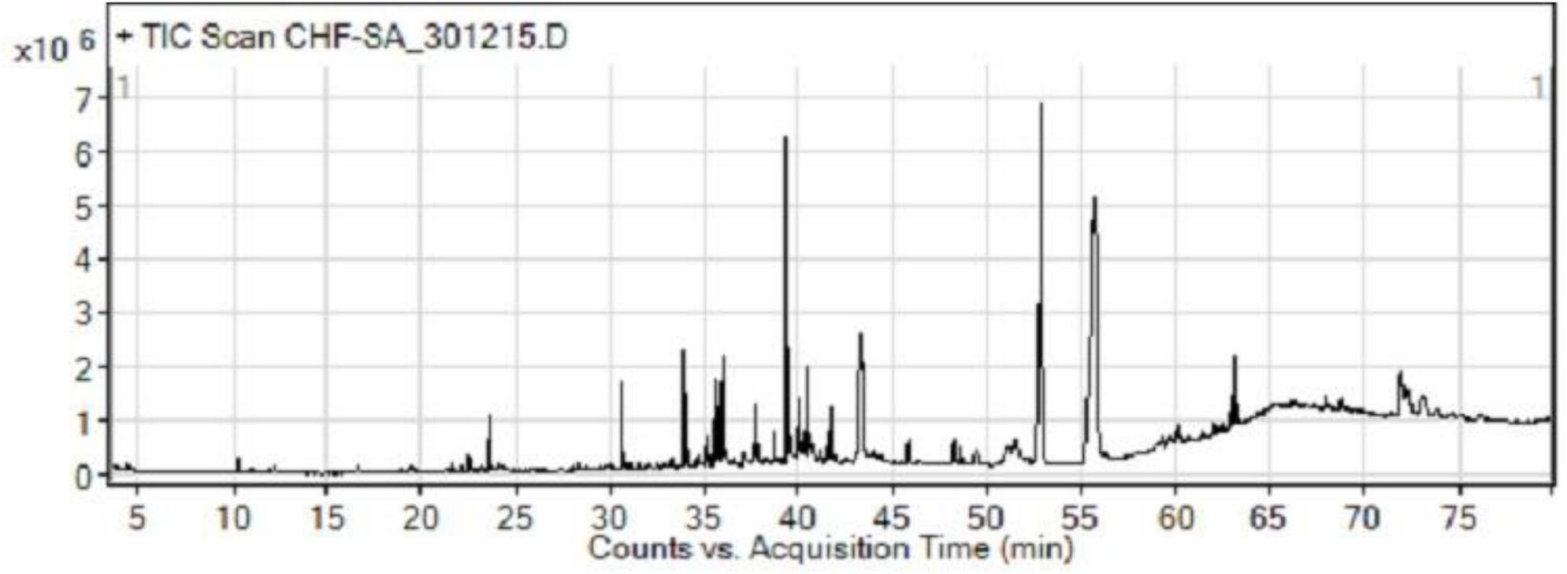
GC-MS chromatogram of chloroform fraction of *Eryngium caeruleum*.

**Figure 3:**
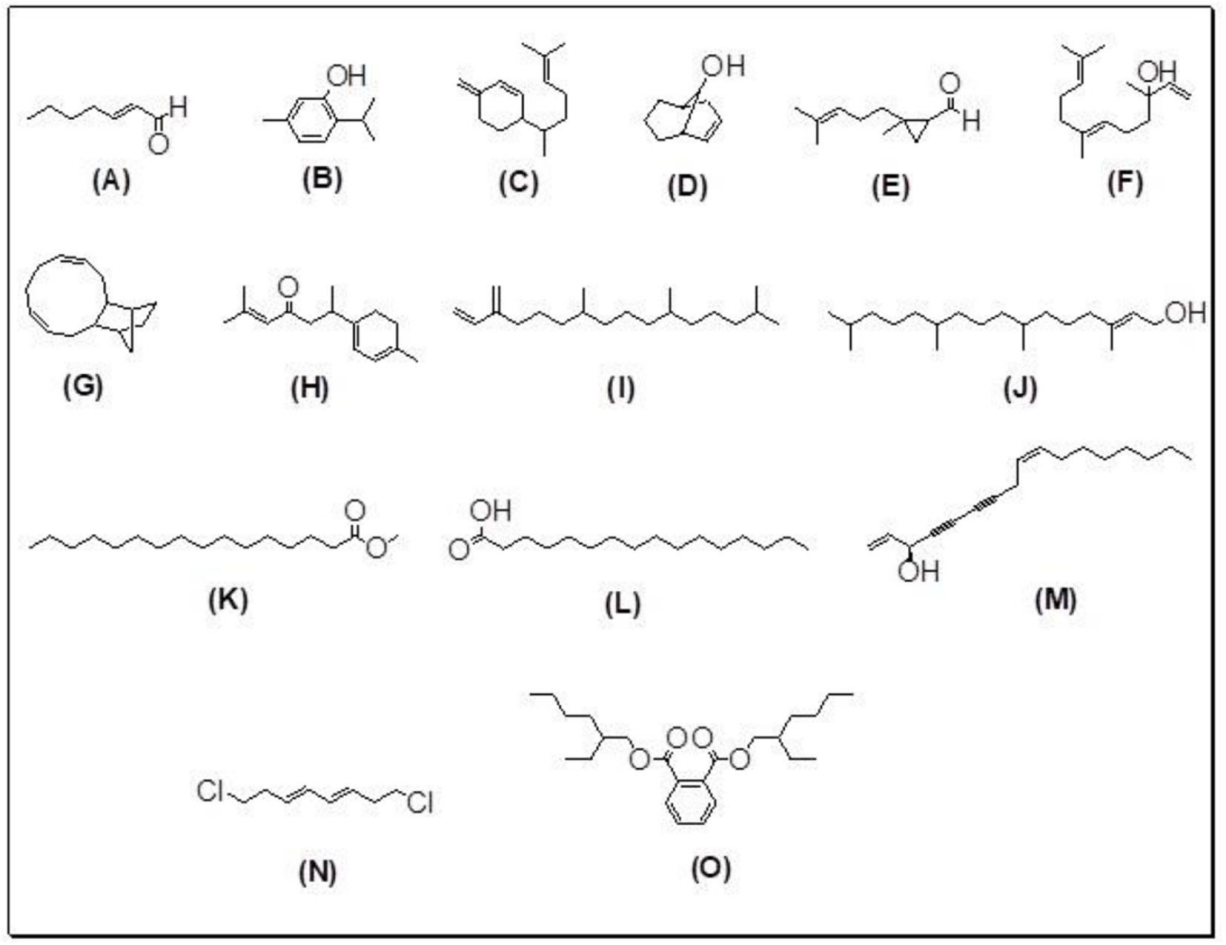
**(A–O)** Major phytoconstituents identified by GC-MS analysis in the chloroform fraction of *Eryngium caeruleum*.

**Figure 4:**
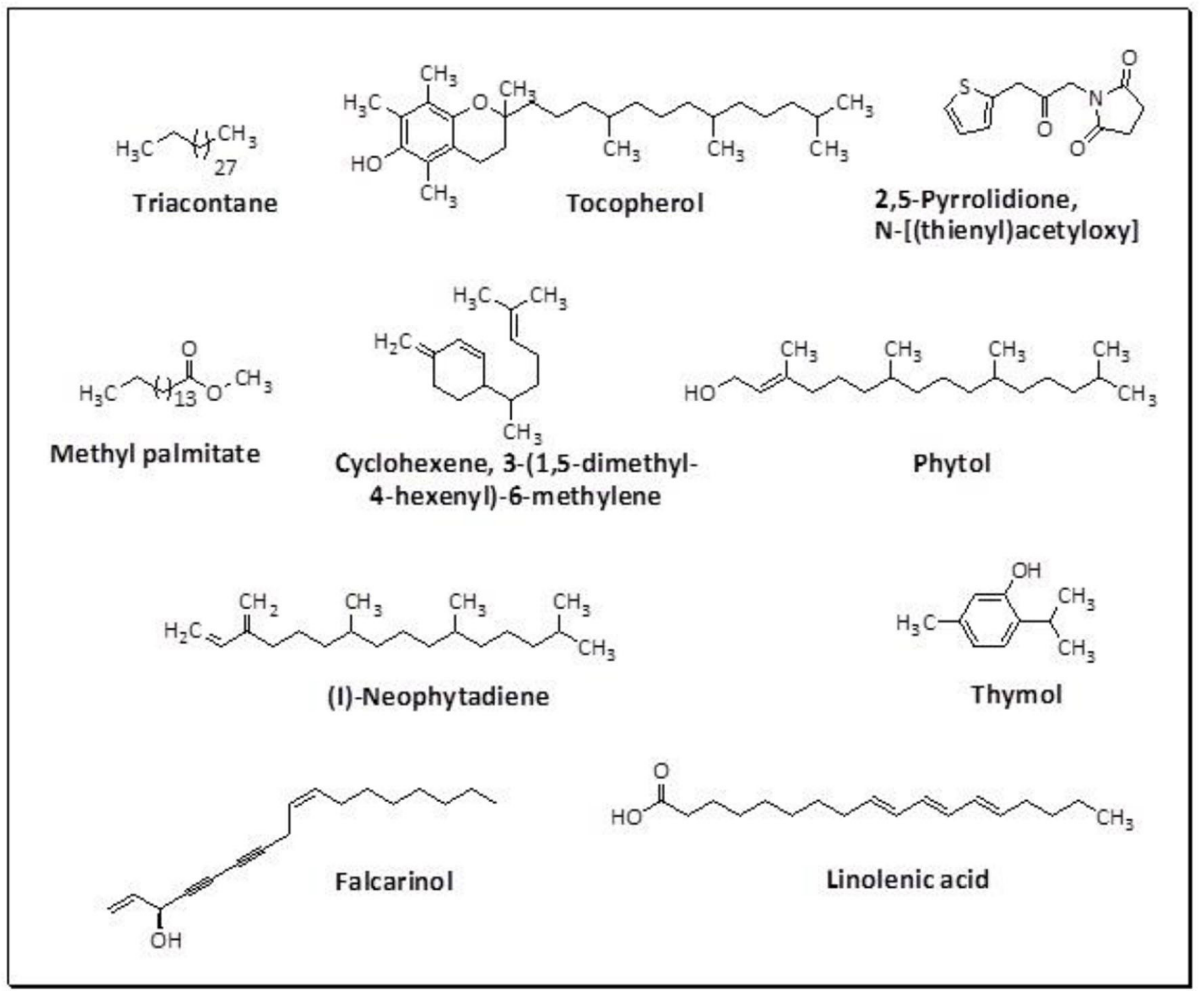
Structures of putative bioactive compounds identified in the chloroform fraction of *Eryngium caeruleum*.

The GC-MS analysis of the most active fraction (i.e., the chloroform fraction was performed as described). A total number of 60 compounds were identified in the GC-MS analysis of chloroform fraction (Table 4). Various parameters provided for the major identified compounds are summarized in Table 5. The chromatogram of this fraction representing various peaks is shown in Figure 2. Out of these compounds, 15 were found to be in dominant concentrations (compounds **A**-**O**), which are summarized in Figure 3. Furthermore, we assessed overall GC-MS analysis of the chloroform fraction and found some interesting bioactive compounds which might be responsible for the proposed activities. These compounds include triacontane, tocopherol, 2,5-phyrrolidione, N-[2-(thienyl)acetyloxyl], methyl palmitate, cyclohexene, 3-(1,5-dimethyl-4-hexenyl)-6-methylene, phytol, neophytadiene, thymol, falcarinol, and linolenic acid as shown in Figure 4.

**Table 4:** Phytoconstituents identified by GC-MS analysis in the chloroform fraction of *Eryngium caeruleum*.

**S.No**	**Compound label**	**RT**	**Common name**	**Formula**
1	Chloroform	3.63	Chloroform	CHCl_3_
2	Hexanal	4.455	Caproaldehyde	C_6_H_12_O
3	Dimethyl sulfoxide	6.052	DMSO	C_2_H_6_OS
4	*trans*-2-Heptenal	10.294	3-Butylacrolein	C_7_H_12_O
5	*trans*-2-Decalone	22.612	trans-2-Decalone	C_10_H_16_O
6	Thymol	23.557	Thymol	C_10_H_14_O
7	2,4-Nonadien-1-al	24.087	NF	C_9_H_14_O
8	3-(1,5-dimethyl-4-hexenyl)-6-methylene	30.711	NF	C_15_H_24_
9	2,6-Dimethyl-2-(4'-methylpent-4'-enyl)-1-oxaspiro[2.5]oct-5-ene	33.367	NF	C_15_H_24_O
10	Bicyclo[3.3.1]non-2-en-9-ol	34.002	NF	C_9_H_14_O
11	Tricyclo[7.1.0.0[1,3]]decane-2-carbaldehyde	34.717	NF	C_11_H_16_O
12	3-Methyl-2-cyclohexen-1-one	35.175	NF	C_7_H_10_O
13	alpha – phellandrene	35.244	α - phellandrene	C_10_H_16_
14	Cyclopropanecarboxaldehyde	35.5	NF	C_11_H_18_O
15	E-farnesol	35.699	Nerolidol	C_15_H_26_O
16	1,4-Methanobenzocyclodecene	35.962	NF	C_15_H_22_
17	Tridecanal	36.112	Tridecanal	C_13_H_26_O
18	1,5-Cycloundecadiene, 8,8-dimethyl-9-methylene-	36.221	NF	C_14_H_22_
19	4-((1E)-3-Hydroxy-1-propenyl)-2-methoxyphenol	37.072	NF	C_10_H_12_O_3_
20	Tetradecanoic acid	37.614	Myristic acid	C_14_H_28_O_2_
21	(+)-.alpha.-Atlantone	37.806	(+)-α-Atlantone	C_15_H_22_O
22	Globulol	38.735	Globulol	C_15_H_26_O
23	2-Vinyladamantane	38.938	2-Vinyladamantane	C_12_H_18_
24	Neophytadiene	39.406	Neophytadiene	C_20_H_38_
25	6,10,14-Trimethyl-pentadecan-2-ol	39.56	NF	C_18_H_38_O
26	(1S,2S)-N-Amino-2-methylamino-1-phenyl-1-propanol	39.934	NF	C_11_H_16_N_2_O
27	3,7,11,15-Tetramethyl-2-hexadecen-1-ol	40.035	NF	C_20_H_40_O
28	Sipomer IBOMA	40.27	Sipomer IBOMA	C_14_H_22_O_2_
29	3,7,11,15-Tetramethyl-2-hexadecen-1-ol	40.496	NF	C_20_H_40_O
30	Dihydro.alpha. ionone	40.64	Dihydro-α-ionone	C_13_H_22_O
31	(7R,8S)-cis-anti-cis-7,8-Epoxytricyclo[7.3.0.0(2,6)]dodecane	41.182	NF	C_12_H_18_O
32	13-Octadecenal	41.589	Z-13-Octadecenal	C_18_H_34_O
33	Hexadecanoic acid, methyl ester	41.731	Methyl palmitate	C_17_H_34_O_2_
34	3,3,5-trimethylcyclohexyl ester	41.95	Arto-espasmol	C_17_H_24_O_3_
35	3-Heptadecen-5-yne	42.434	NF	C_17_H_30_
36	Cyclobutaneacetonitrile	42.88	NF	C_10_H_15_N
37	Palmitic acid, methyl ester	43.364	Palmitic acid	C_16_H_32_O_2_
38	Dodecene	43.972	Dodecene	C_12_H_24_
39	Hexadecanoic acid, ethyl ester	43.991	Ethyl palmitate	C_18_H_36_O_2_
40	Sinapic Alcohol	44.369	Sinapic Alcohol	C_11_H_14_O_4_
41	Falcarinol	45.799	Falcarinol	C_17_H_24_O
42	9,12-Octadecadienoic acid, methyl ester	48.272	NF	C_19_H_34_O_2_
43	9,12-Octadecadienoyl chloride	48.591	Linoleoyl chloride	C_18_H_31_ClO
44	Phytol	49.425	Phytol	C_20_H_40_O
45	Octadecadienoic acid, methyl ester	51.139	NF	C_18_H_32_O_2_
46	Linolenic acid, methyl ester	51.53	Linolenic acid	C_18_H_30_O_2_
47	Cyclobuta[1,2:3,4]dicyclooctene	52.137	NF	C_16_H_28_
48	(3E,5E)-1,8-Dichloro-3,5-octadiene	55.744	NF	C_8_H_12_Cl_2_
49	Cyclohexane, 1,1'-(1-methyl-1,3-propanediyl)bis	59.287	NF	C_16_H_30_
50	Dodecanal	59.676	Lauraldehyde	C_12_H_24_O
51	2,5-Pyrrolidione, N-[2-(thienyl)acetyloxy]-	60.128	NF	C_10_H_9_NO_4_S
52	5,6-c(13)(2)-1,5,9-decatriyne	62.201	NF	C_10_H_10_
53	Eicosane	62.496	Eicosane	C_20_H_42_
54	Tetradecanal	62.891	Myristaldehyde	C_14_H_28_O
55	1,2-Benzenedicarboxylic acid, bis(2-ethylhexyl) ester	63.058	DNOP	C_24_H_38_O_4_
56	Triacontane	63.263	Triacontane	C_30_H_62_
57	n-Cetyl thiocyanate	63.763	n-Cetyl thiocyanate	C_17_H_33_NS
58	3,8-Dimethyldecane	64.709	NF	C_12_H_26_
59	Alpha.-Tocopherol	69.784	Vitamin E	C_29_H_50_O_2_
60	16-Hentriacontanone	72.349	Palmitone	C_31_H_62_O

**Table 5:** Chromatogram peak list of the major identified compounds in chloroform fraction of *Eryngium caeruleum*.

**RT**	**Height**	**Height %**	**Area**	**Area %**	**Area sum %**	**Base peak m/z**	**Width**
23.559	985159	16.87	3776010	17.89	5.62	135	0.218
30.712	2E+06	26.9	5001972	23.7	7.44	69.1	0.151
34.001	2E+06	36.7	7934080	37.59	11.81	120.1	0.231
35.501	774377	13.26	2639538	12.51	3.93	69.1	0.141
35.699	1E+06	23.32	4177734	19.8	6.22	69	0.121
35.963	2E+06	31.84	7224093	34.23	10.75	91.1	0.161
37.805	860399	14.73	2717830	12.88	4.04	83	0.127
39.406	6E+06	100	21104837	100	31.41	68.1	0.171
40.036	889000	15.22	2755271	13.06	4.1	81.1	0.121
40.495	2E+06	26.43	5332836	25.27	7.94	81.1	0.131

### Results of HPLC-DAD Analysis

**Figure 5:**
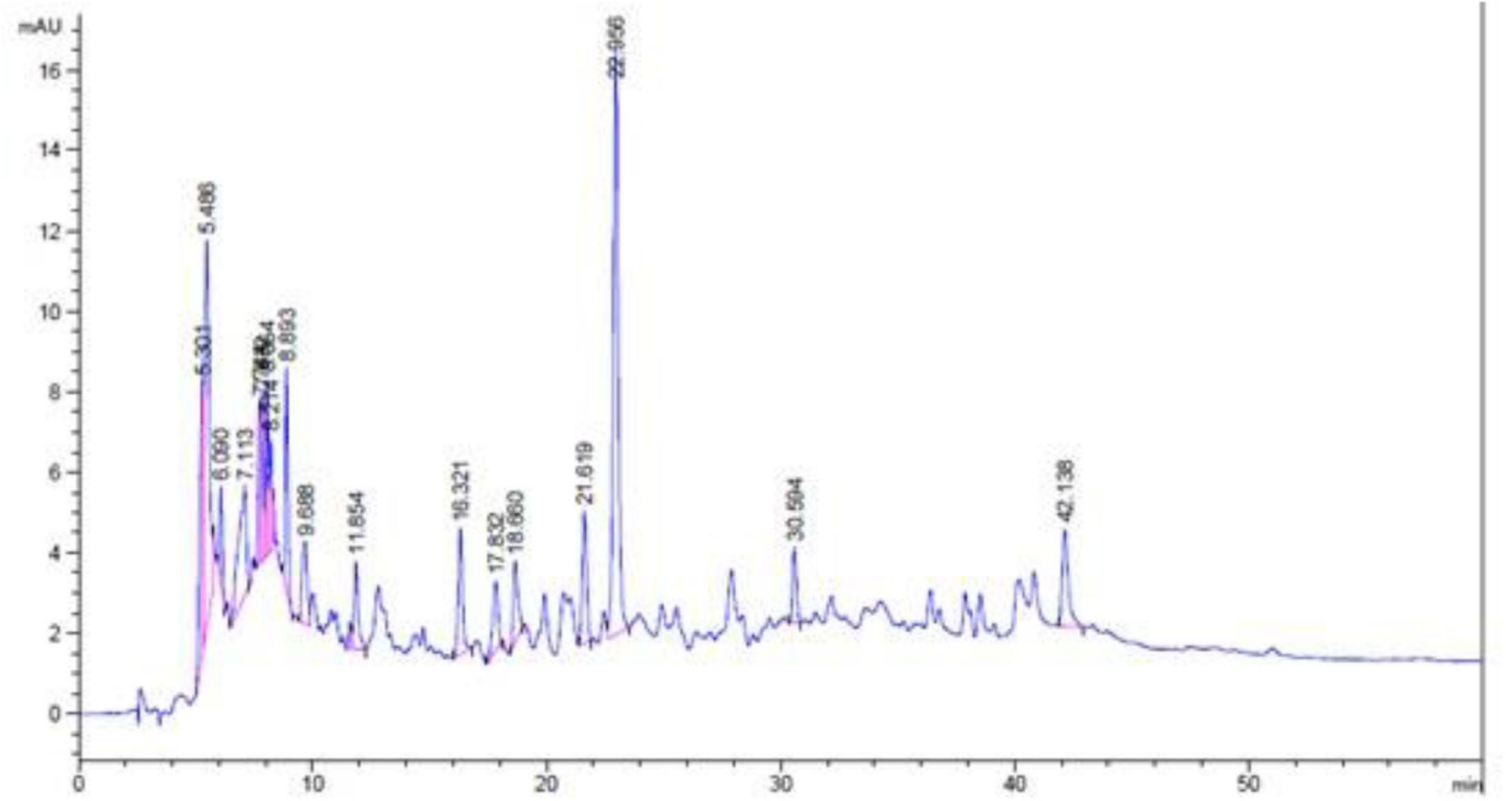
HPLC-DAD chromatogram of methanolic extract of *Eryngium caeruleum*.

**Figure 6:**
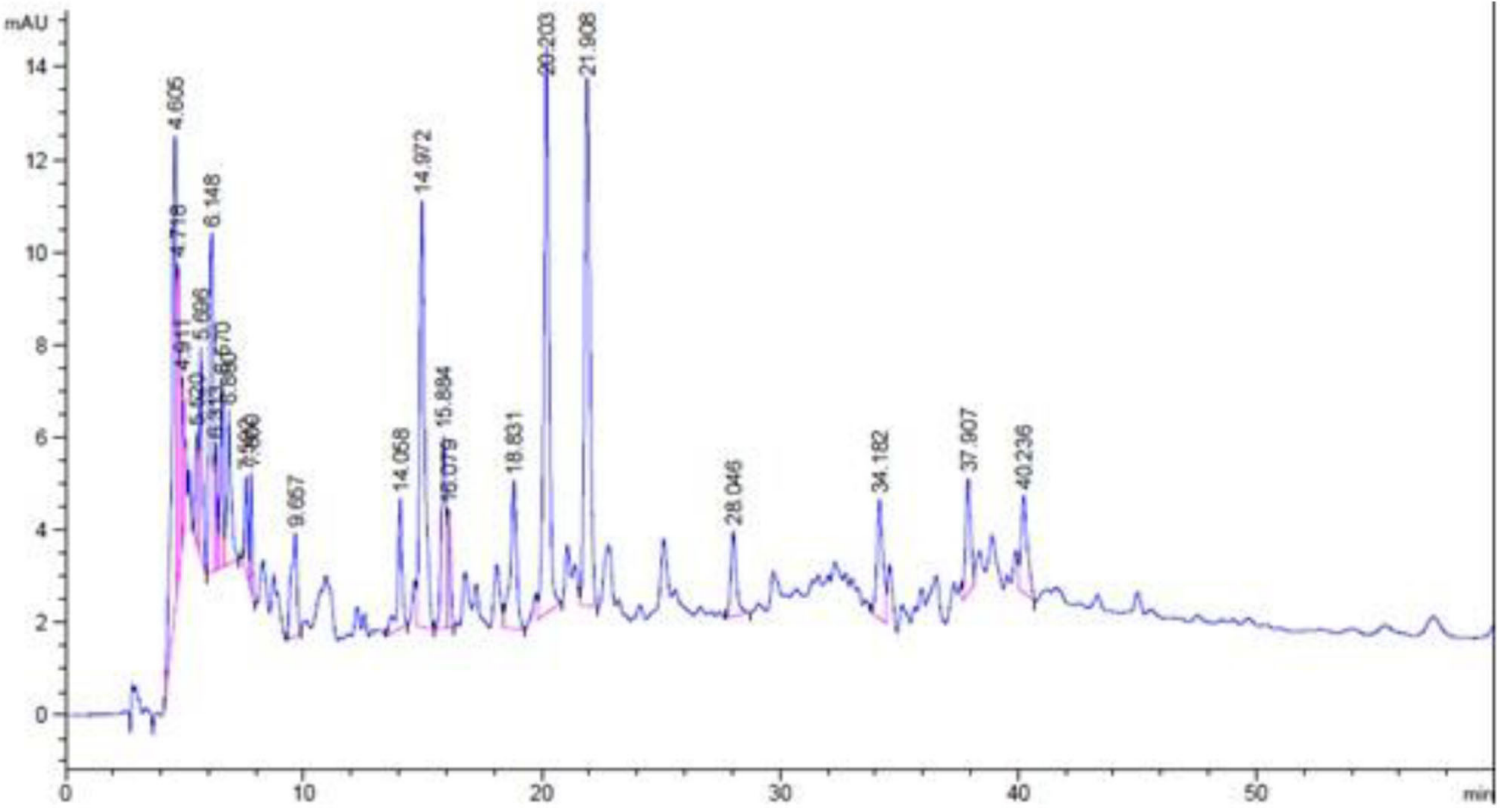
HPLC-DAD chromatogram of chloroform fraction of *Eryngium caeruleum*.

The methanolic extract and chloroform fraction of *E. caeruleum* were analyzed via HPLC-DAD. The chromatograms obtained for methanolic extract and chloroform fraction has been represented in Figures 5, 6, respectively.

In the chromatogram of methanolic extract, various components are separated in different ratios. The ratios of some distinct peaks are summarized in Table 6 with their retention times. The highest concentrated signal was observed at a retention time of 22.96 min which was 22.78%. All the other signals especially after 10 min, were smaller. On the other hand, the HPLC spectrum of chloroform fraction was observed to be different with respect to the nature and ratios of components. After retention time 10, various peaks were observed in dominant ratios, which shows the possible solubility of these components in chloroform. The dominant peaks after a retention time of 10 min were observed at 14.06, 14.97, 15.89, 16.08, 18.83, 20.20, 21.91, 28.05, 34.18, 37.91, and 40.24 min. All the peaks with their retention times and ratios are shown in Table 7.

**Table 6:** Various parameters of peaks obtained in HPLC-DAD chromatogram in the methanolic extract of *Eryngium caeruleum*.

**Peak**	**Ret time [min]**	**Type**	**Width [min]**	**Area [mAU × s]**	**Height [mAU]**	**Area %**
1	5.301	BV	0.1306	64.72926	6.76151	7.0369
2	5.486	VV	0.2125	146.56313	9.63225	15.9334
3	6.090	BB	0.1117	18.74151	2.43263	2.0375
4	7.113	BB	0.3071	66.59585	2.73729	7.2399
5	7.744	BV	0.1154	28.22402	4.01753	3.0683
6	7.832	VV	0.1156	32.61134	4.18651	3.5453
7	8.054	VV	0.1051	31.43048	4.40266	3.4169
8	8.214	VV	0.1002	19.31980	2.82343	2.1003
9	8.893	BB	0.1190	43.60714	5.56735	4.7407
10	9.688	BV	0.2554	31.76944	2.04627	3.4538
11	11.854	VB	0.1754	26.77760	2.20154	2.9111
12	16.321	BB	0.1898	39.09953	3.12287	4.2506
13	17.832	BB	0.2280	25.88051	1.70597	2.8136
14	18.660	BB	0.1968	26.97938	2.01758	2.9330
15	21.619	VB	0.2038	42.41638	3.26999	4.6112
16	22.956	VB	0.2177	209.56824	14.54069	22.7829
17	30.594	BB	0.1820	21.77686	1.87696	2.3674
18	42.138	BB	0.2679	43.75992	2.41850	4.7573

**Table 7:** Various parameters of peaks obtained in HPLC-DAD chromatogram in the chloroform fraction of *Eryngium caeruleum*.

**Peak**	**Ret time [min]**	**Type**	**Width [min]**	**Area [mAU × s]**	**Height [mAU]**	**Area %**
1	4.605	BV	0.1951	135.09828	10.21325	9.9527
2	4.718	VV	0.1219	61.16179	6.92654	4.5058
3	4.911	VV	0.0931	23.56275	3.56647	1.7359
4	5.520	BV	0.1336	22.98246	2.39954	1.6931
5	5.696	VB	0.1215	38.69032	4.53148	2.8503
6	6.148	BV	0.1933	86.46613	7.31094	6.3699
7	6.313	VV	0.0975	18.76769	2.73718	1.3826
8	6.570	VV	0.1379	34.73483	4.05182	2.5589
9	6.860	VB	0.1642	39.82209	3.33267	2.9337
10	7.592	BV	0.1341	17.76695	2.06061	1.3089
11	7.800	VB	0.0930	15.16980	2.53777	1.1176
12	9.657	BV	0.2242	36.80556	2.20064	2.7115
13	14.058	BB	0.1986	37.91294	2.80185	2.7930
14	14.972	VB	0.2261	140.51132	9.20776	10.3514
15	15.884	BV	0.2004	55.64159	4.14288	4.0991
16	16.079	VB	0.1446	24.09252	2.52876	1.7749
17	18.831	VB	0.2633	58.15710	3.19538	4.2844
18	20.203	VB	0.2232	182.45099	12.25521	13.4411
19	21.908	VB	0.2403	178.07169	11.41171	13.1185
20	28.046	BB	0.2432	29.90574	1.84323	2.2031
21	34.182	BV	0.2501	44.41979	2.56465	3.2724
22	37.907	VV	0.2304	37.03683	2.48826	2.7285
23	40.236	VB	0.2687	38.17921	2.08760	2.8127

### Results of Docking Studies

Our research group has already explored the synergistic effects of constituents of different plant extracts against α-glucosidase *via* docking simulations (Nadeem et al., 2019, 2020a,b; Farooq et al., 2020). Hence, we also performed docking simulations in order to correlate the *in-vitro* results and to explore the possible role of putative bioactive compounds identified in the chloroform fraction of *Eryngium caeruleum*. A Molecular Operating Environment (MOE) software package was used for this purpose. These putative bioactive compounds identified in the chloroform fraction of *Eryngium caeruleum* were tested *in-vitro* against α-glucosidase from Baker's yeast. The 3-D structure of Saccharomyces cerevisiae (Baker's yeast) has not yet been reported. Hence, we performed docking simulations by using our previously reported Homology modeled α-glucosidase (Ali et al., 2018).

**Figure 7:**
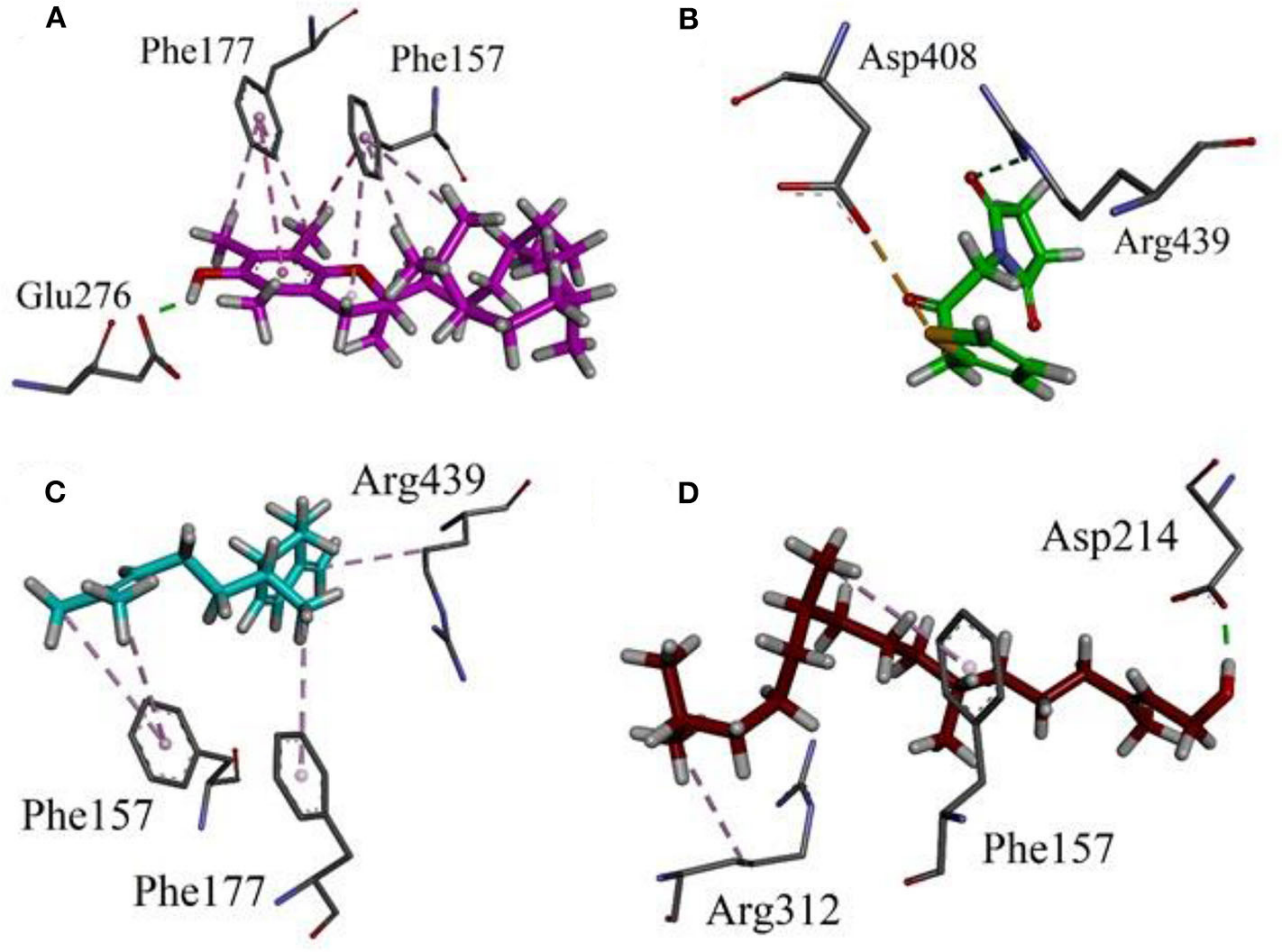
Binding orientation and 3-D interaction plot of putative bioactive compounds identified in the chloroform fraction of *Eryngium caeruleum* into active sites of homology model of α-glucosidase. **(A)** tocopherol **(B)** 2,5-Pyrrolidione, N-[(thienyl) acetyloxy] **(C)** Cyclohexene, 3-(1,5-dimethyl-4-hexenyl)-6-methylene, and **(D)** phytol.

**Figure 8:**
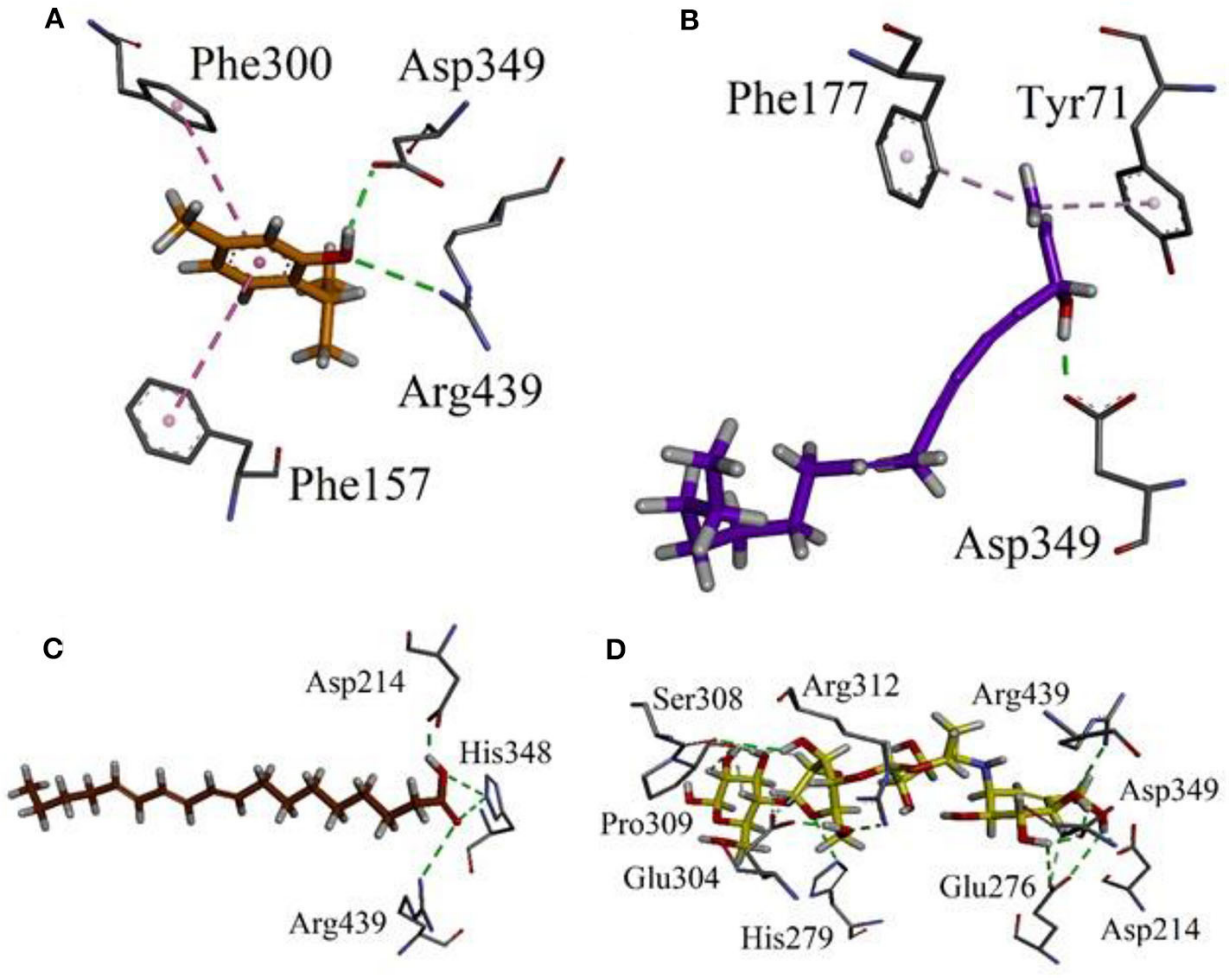
Binding orientation and 3-D interaction plot of putative bioactive compounds identified in the chloroform fraction of *Eryngium caeruleum* into active sites of homology model of α-glucosidase. **(A)** thymol **(B)** Falcarinol **(C)** linolenic acid and **(D)** standard drug acarbose.

The 3-D binding orientations and interaction plots are shown in Figures 7, 8. While the binding energy data and list of interacting residues are shown in Table 8. Tocopherol, phytol, thymol, Falcarinol and linolenic acid form hydrogen bond interactions with the residues present in the catalytic triad (Asp214, Glu276, and Asp349). A number of active constituents' form π-π stacking with key amino acid residues; Phe157, Phe177, Phe300 (Table 8) (Nadeem et al., 2019, 2020a,b; Farooq et al., 2020). Tocopherol has shown the most stable ligand-enzyme complex with the binding energy −7.7008 kcal/mol. While, phytol and linolenic acid also showed the lowest binding energy (−7.0629 and −7.01746 kcal/mol, Table 8).

**Table 8:** Binding energy values and ligand interaction pattern revealed by possible isolated phytoconstituents and acarbose toward yeast α-glucosidase.

**Compounds**	**Binding energy^a^**	**Interacting residues**	**Figure no**.
Tocopherol	−7.7008	Glu276 (HB^b^), Phe157 and Phe177 (π-π)	Figure 7A
2,5-Pyrrolidione, N-[(thienyl) acetyloxy]	−5.5557	Arg439 (HB), Asp408 (π-sulfur)	Figure 7B
Cyclohexene, 3-(1,5-dimethyl-4-hexenyl)-6-methylene	−5.5954	Phe157, Phe177 and arg439 (π-alkyl)	Figure 7C
Phytol	−7.0629	Asp214 (HB), Phe157 and Arg312 (π-alkyl)	Figure 7D
(I)-Neophytadiene	−6.5832	Tyr71, His111, His239, Phe300, His348 (π-alkyl)	**-**
Thymol	−4.5388	Asp349, Arg439 (HB), Phe157, Phe300 (π-π)	Figure 8A
Falcarinol	−6.2175	Asp349 (HB); Tyr71 and Phe177 (π-alkyl)	Figure 8B
Linolenic acid	−7.1746	Asp214, His348 and Arg439 (HB)	Figure 8C
Acarbose (Standard)	−9.2756	Asp214, His279, Glu304, Pro309, Arg312, Asp349, Arg439	Figure 8D

a *in kcal/mol*.

b *HB, hydrogen bond*.

Subsequently, we docked major phytoconstituents identified by GC-MS analysis (Figure 3) into the homology model of α-glucosidase. The binding energy data of the docked compounds is shown in Table 9. The binding energy of these compounds were found in the range of −6.6118 to −4.0372 kcal/mol. Highest activity was shown by bis(2-ethylhexyl) phthalate (**O** in Figure 3).

**Table 9:** Binding energy values and ligand interaction pattern revealed by possible isolated phytoconstituents and acarbose toward yeast α-glucosidase.

**Compounds**	**Binding energy^a^**	**Compounds**	**Figure no**.
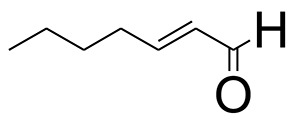	−4.4117	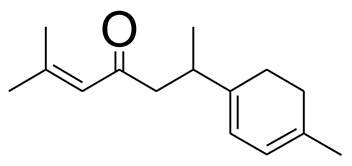	−6.2061
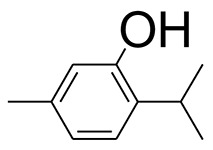	−4.3642	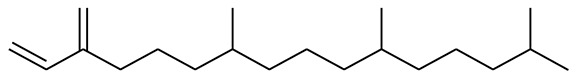	−4.0372
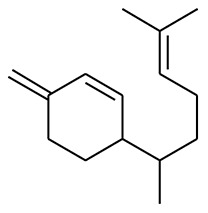	−6.2961	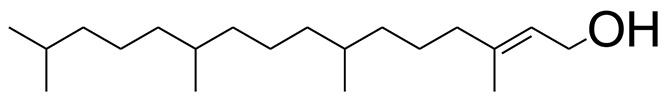	−5.5123
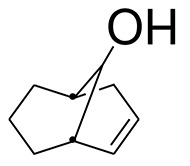	−5.9947	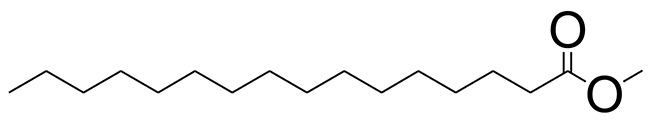	−5.0339
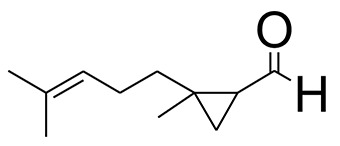	−4.8546	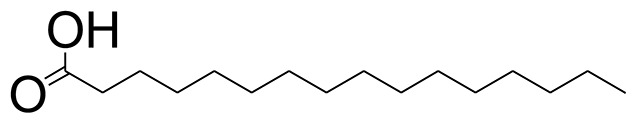	−5.9452
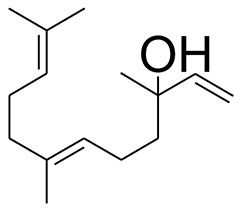	−5.9155	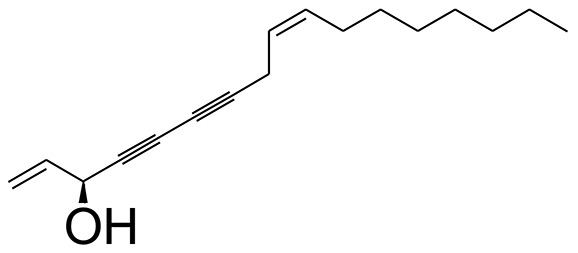	−6.2175
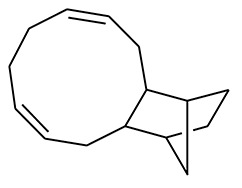	−4.8760	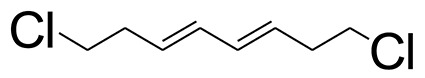	−4.9297
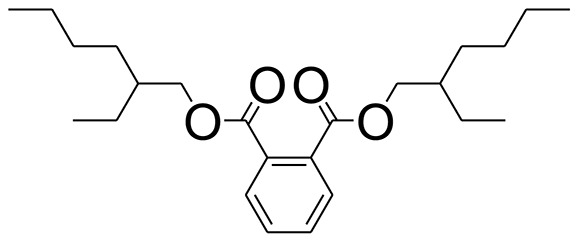	−6.6118	Acarbose (Standard)	−9.2756

a *in kcal/mol*.

## Discussion

Alpha-glucosidase inhibitors represent a group of antidiabetic drugs that regulate postprandial hyperglycemia through inhibition of carbohydrates digestion in the small intestine and thus hamper the diet associated with acute glucose excursion (Dwek et al., 2002). Among these drugs, acarbose and voglibose have got substantial attention in the past decades due to their effectiveness in controlling type 2 DM (Scheen, 2009). Acarbose is a natural product obtained from the fermentation of *Actinoplanes* species (Truscheit et al., 1981). Apart from presently available drugs, several herbal medicines have been recommended for the management DM. Traditional medicinal plants and plants-based remedies are used all over the world for a range of diseases, including DM (Aslam et al., 2018). The hypoglycaemic activity of medicinal plants including *Euclea undulata* var. *myrtina, Schkuhria pinnata, Pteronia divaricata*, and *Elaeodendron transvaalense* which are traditionally used against DM were scientifically proved (Deutschländer et al., 2009). Different solvent extracts from *Calamintha origanifolia, Satureja thymbra, Prangosasperula, Sideritis perfoliata, Asperula glomerata, Hyssopus officinalis, Erythraea centaurium, Marrubium radiatum*, and *Salvia acetabulosa* were recently reported to possess strong anti- glucosidase potentials (Loizzo et al., 2008). Likewise, the solvent extracts prepared from *Azadirachta indica, Murraya koenigii, Ocimum tenuflorum, Syzygium cumini, Linum usitatissimum*, and *Bougainvillea spectabilis* which are ethno-botanically known medicinal plants against DM was reported to possess anti-glucosidase potentials (Bhat et al., 2011).

In the current study, we screened various solvent extracts from *Eryngium caeruleum* against α-glucosidase enzyme and a number of free radicals both of which are important targets in the management of type 2 DM. Although the percent of inhibitions of crude extracts were low in comparison to the standard drug, the presence of active anti-glucosidase constituents were still observed. The Ec.Chf was observed to be more potent as compared to other fractions in all *in-vitro* assays. Similarly, the Ec.Chf was also found to be effective in *in-vivo* antidiabetic assay at various concentrations. The first extracted fraction was with n-hexane which probably showed less potency due to the presence of non-polar and inactive compounds. In comparison, the next two fractions (i.e., with chloroform and ethyl acetate were relatively effective). One of the reasons for their effectiveness might be the solubility of active polar organic compounds in it. The remaining aqueous fraction was the least active. A major reason was that the majority of the active polar compounds tend to be extracted with chloroform and ethyl acetate, leaving a very small ratio of remaining compounds in the last fraction. For the preliminary identification of active compounds GC followed by GC-MS analysis of the active fraction was performed. We observed that 15 compounds were in greater concentration. Apart from the GC-MS analysis, further fingerprinting of *E. caeruleum* has been performed with the help of HPLC-DAD. The GC-MS and HPLC-DAD reveal the importance of *E. caeruleum* in terms of natural compounds.

Experimental evidence implies the contribution of free radicals in the inception of DM and more significantly in the progress of diabetic complications (Zeb et al., 2014). Free radical scavengers are useful in the prevention of DM in experimental diabetic animal models (Sadiq et al., 2018). Unrelenting hyperglycemia in DM patients can lead to oxidative stress via auto-oxidation of glucose, non-enzymatic glycosylation and polyol pathways. Auto-oxidation of glucose results from the natural reduction of molecular oxygen species to superoxide and hydroxyl radicals which, being extremely reactive, reacts with all biomolecules (Sadiq et al., 2015b; Jabeen et al., 2018). These free radicals also speed up formation of advanced glycation end products like pyrroles and imidazoles. Cross-linking of these glycation end products with proteins in tissues fallout as irregularities in the function of cells and tissues. The third mechanism of free radical generation is Polyol pathway during which a greater amount NADPH is diminished which impair the formation of key antioxidants like glutathione (Giugliano et al., 1995). As a result, the protein glycation capability of antioxidant enzymes is also decreased. Long-lived structural proteins, collagen and elastin, experience persistent non-enzymatic cross-linking during aging, and in diabetic individuals (Vasan et al., 2003). Chronic oxidative stress also leads to insulin resistance, dyslipidemia, β-cell dysfunction, impaired glucose tolerance and type-2 DM. Prolonged oxidative stress, hyperglycemia and dyslipidemia are principally harmful for β-cells. The impairment of β-cell function leads to under production of insulin, weakened glucose-stimulated insulin secretion, fasting hyperglycemia, and ultimately the development of type-2 DM (Tangvarasittichai, 2015).

As Ec.Chf was the most active fraction in α-glucosidase inhibition and antioxidant assays, so it was subjected to *in-vivo* and GC-MS analysis for preliminary identification of bioactive compounds. The GC-MS resulted in the identification of 60 compounds. Out of these, 15 compounds were found to represent the major peaks of the spectrum. Several compounds identified in the GC-MS data were found active in terms of anti-diabetic and antioxidant potentials as evident from their reported literature. In anti-diabetic context, these include thymol, nerolidol, neophytadiene, falcarinol, linolenic acid, palmitic acid, pyrrolidione derivative etc. Briefly, the thymol a monoterpene isolated from *Thymus vulgaris* and reported to possess antioxidant and anti-diabetic potentials (Hyun et al., 2014). Nerolidol and neophytadiene are terpenes found in many plants, for example, it has been found recently in the extract of *Achillea ligustica* and in the extract was also assayed for its anti-radical and anti-diabetic potentials with excellent results (Conforti et al., 2005). Similarly, falcarinol, a known pesticide which is also found in some plant extracts along with anti-diabetic activity (An et al., 2014). In the same way, linolenic acid has also been reported to possess anti-diabetic potential (Kato et al., 2000). Palmitic acid has also been proven to be an excellent candidate in type-2 diabetes mellitus (Chae et al., 2010). Correspondingly, the pyrolidone derivative has been proven to possess strong α-glucosidase inhibitory and anti-inflammatory properties (Trapero and Llebaria, 2012; Jan et al., 2020). Moreover, several antioxidant compounds identified in the GC-MS spectrum have been reported to scavenge the free radicals effectively. Some of which include thymol, tocopherol, palmitate, phytol, triacontane, cyclohexene, 3-(1,5-dimethyl-4-hexenyl)-6-methylene etc. The thymol has been verified previously to possess strong antioxidant potential (Braga et al., 2006). The vitamin E is also an excellent antioxidant compound used in various diseased conditions (Müller et al., 2010). In the same way, ascorbyl palmitate has been reported to possess antioxidant properties (Hra et al., 2000). The antioxidant activity of phytol has been demonstrated by several investigators (Santos et al., 2013). Likewise, the 16-hydroxy-18-Triacontane and 4-hydroxy-triacontane-16,18-dione has been isolated as a natural antioxidant from Eucalyptus leaf (Osawa and Namiki, 1985). The cyclohexene, 3-(1,5-dimethyl-4-hexenyl)-6-methylene was previously identified in the Karungkuravai (a type of medicinal rice) and assayed for antioxidant activity with positive results (Krishnanunni et al., 2015). All the putative bioactive compounds identified in the Ec.Chf are shown in Figure 3. The identification of numerous bioactive compounds in the Ec.Chf and the current investigational studies shows the role of *E. caeruleum* in the management of oxidative stress and diabetes mellitus.

As discussed in the Introduction section, the isolated two new flavone glycosides reported by Rehman et al. were evaluated for their potential against α-glucosidase, aldose reductase (ALR1 and ALR2). Compounds were only found active against ALR1 and ALR2 and were not able to show α- β-glucosidase inhibition and antiglycation potential. Docking studies were performed only on aldose reductase targets. In the current study, we performed a docking experiment to justify the synergistic effect of all these isolated bioactive compounds. The data obtained from docking studies agrees with the α-glucosidase inhibition activity. The computed binding energy of standard drug acarbose is −9.2756 kcal/mol. Putative bioactive compounds showed binding energy values in the range of −7.7008 to −4.5388 kcal/mol. Among simulated constituents, tocopherol showed best binding energy (−7.7008 kcal/mol). While the binding energy from major phytoconstituents identified by GC-MS analysis were found in the range of −6.6118 to −4.0372 kcal/mol. The highest affinity was shown by bis(2-ethylhexyl) phthalate (**O** in Figure 3). The binding energy data showed in Tables 8, 9 revealed that all the compounds identified in the chloroform fraction of *Eryngium caeruleum* are responsible for the synergistic effect of hypoglycemia.

## Conclusions

The sorting out of the most active solvent fraction of *Eryngium caeruleum* in the terms of antioxidant and anti-diabetic potentials followed by its GC-MS and HPLC-DAD analyses revealed that this plant is an excellent source of bioactive compounds. Based on the literature survey of identified compounds and ethnopharmacology of *Eryngium caeruleum*, it may be concluded that this plant is rich in antioxidant and anti-diabetic compounds and may be an effective remedy against various diseases, especially free radicals mediated and diabetic mediated disorders. Binding energy data computed via docking studies revealed that all putative bioactive compounds identified in the chloroform fraction of *Eryngium caeruleum* can inhibit α-glucosidase synergistically to treat hyperglycemia.

## Data Availability Statement

The raw data supporting the conclusions of this article will be made available by the authors, without undue reservation.

## Ethics Statement

The animal study was reviewed and approved by the Ethical Committee, Department of Pharmacy, Faculty of Biological Sciences, University of Malakand, Pakistan as per the animals Bye-Laws 2008 (Scientific Procedure Issue-I).

## Author Contributions

SA, FU, and MA helped in *in-vitro* assays. IK helped in *in-vivo* experiment. MZ performed the HPLC analysis and helped in antioxidant assay. UR performed the molecular docking studies. MFA, RU, ON, Z-UI, and WA helped in GC-MS analysis and enzyme assay. AS supervised the whole project and drafted the manuscript for publication. All the authors have read the manuscript and approved for submission.

## Conflict of Interest

The authors declare that the research was conducted in the absence of any commercial or financial relationships that could be construed as a potential conflict of interest.

## Supplementary Material

Supporting Information 1 and 2 The GC-MS analysis and fragmentation pattern of major and bioactive identified compounds.
