# Supplementary material for: Treating Hyperglycemia From Eryngium caeruleum M. Bieb: In-vitro α-Glucosidase, Antioxidant, in-vivo Antidiabetic and Molecular Docking-Based Approaches
Source: Front Chem. 2020 Nov 26;8:558641. doi: 10.3389/fchem.2020.558641 (PMC7737655; doi:10.3389/fchem.2020.558641)
Supplement: Supporting Information 1 and 2 — The GC-MS analysis and fragmentation pattern of major and bioactive identified compounds. [file Data_Sheet_1.PDF]

Treating hyperglycemia from *Eryngium caeruleum* M. Bieb: *In-vitro* α-glucosidase, antioxidant, *in-vivo* antidiabetic and molecular docking-based approaches

Abdul Sadiq*[1], Umer Rashid[2], Sadiq Ahmad[1], Mohammad Zahoor[3], Farhat Ullah[1], Muhammad Ayaz[1], Muhammad Iftikhar Khan[4] and Zia-Ul Islam[5]

[1]Department of Pharmacy, Faculty of Biological Sciences, University of Malakand, Chakdara, 18000 Dir (L), KP, Pakistan.

[2]Department of Chemistry, COMSATS University Islamabad, Abbottabad Campus, Abbottabad 22060, Pakistan

[3]Department of Chemistry, University of Malakand, Chakdara, 18000 Dir (L), KP, Pakistan.

[4]Department of Pharmacy, COMSATS University Islamabad, Abbottabad Campus, Abbottabad 22060, Pakistan

[5]Department of Biotechnology, Abdul Wali Khan University Mardan, KP, Pakistan

Corresponding author (*): Dr. Abdul Sadiq, Associate Professor, Department of Pharmacy, Faculty of Biological Sciences, University of Malakand, Chakdara 18000, Dir (L), KP, Pakistan. Email: sadiquom@yahoo.com; Contact No. +92(0)301-2297 102

Supporting Information-I

Homology model of α-Glucosidase

The compounds were tested against α-glucosidase from Saccharomyces cerevisiae (Baker's yeast). To date, the X-ray crystallographic structure of this α-glucosidase has not yet been reported. In order to carry out docking studies of the tested compounds, a 3D structure of S. cerevisiae α-glucosidase was constructed by using Modeller 9.18 software (http://salilab.org/modeller/). The sequence of α-glucosidase in FASTA format was retrieved from UniProt (code P53341). The 3D structure of isomaltase from S. cerevisiae (PDB 3AJ7) was selected as template sequence (72.4% similarity). Best model was selected from five models and refinement of the constructed model (97.3 % amino acids were positioned in favored regions, 1.7% in allowed regions and 1.0% in outlier region) was validated by Ramachandran plot. The constructed model was also evaluated using ERRAT, energy minimization with Z-score using QMEAN (Qualitative Model Energy ANalysis, https://swissmodel.expasy.org/qmean/)

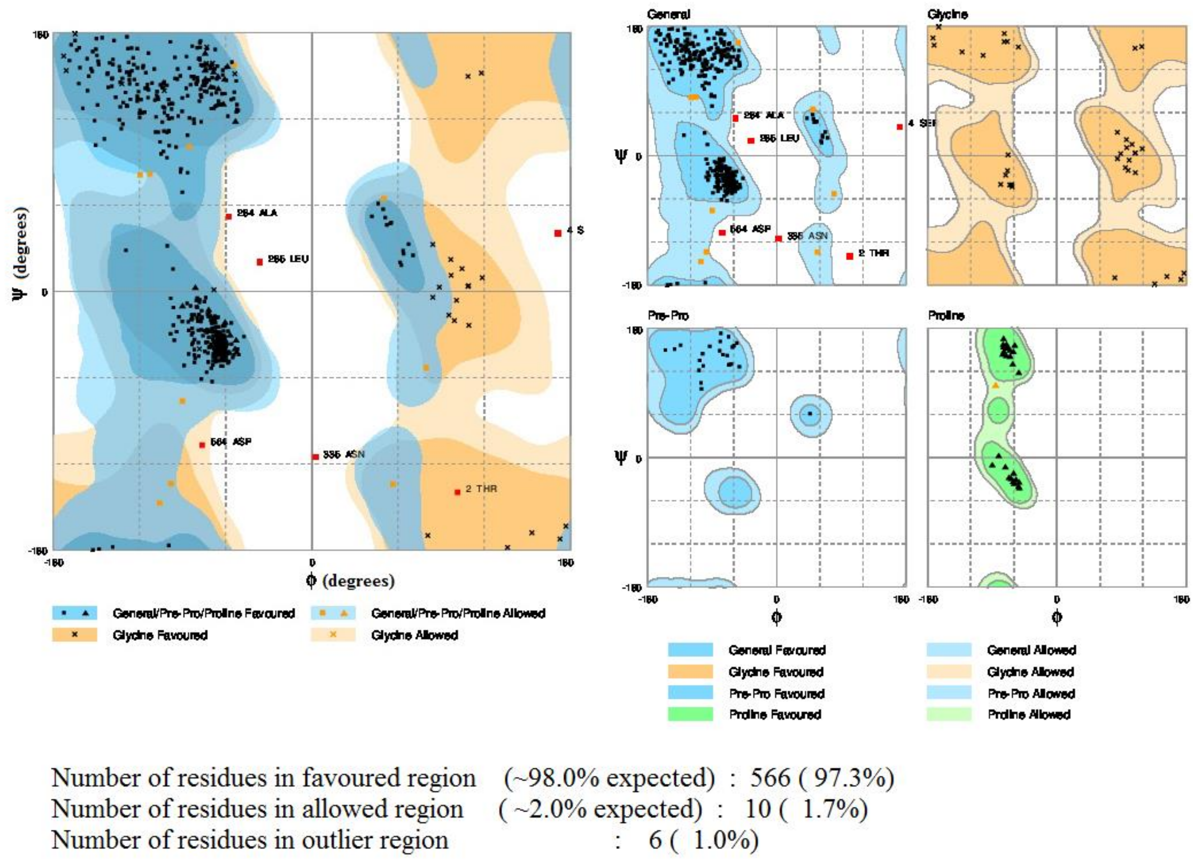

Figure S-1: Ramachandran plot of homology modelled α-glucosidase generated by Rampage.

Docking studies

Molecular docking studies were made by Molecular-Operating Environment (MOE-2016.08) [37]. Docking study for α-glucosidase enzyme was performed on our previously reported homology modelled α-glucosidase [38]. Preparation of ligand's downloaded enzymes such as determination of binding sites, energy minimization and 3-D protonation was performed by previously reported methods [39-41]. Docking results were interpreted, and their surfaces were analyzed with graphical representation utilizing discovery-studio visualizer.

Preparation of ligands and downloaded enzymes (3D protonation, energy minimization and determination of binding site was carried out by our previously reported methods. All the

ligand structures were drawn using Builder option in MOE. A data base of compounds was built as ligand.mdb. The compounds were then energy minimized upto 0.01 Gradient using MMFF94X forcefield.

The 3D protonation of modelled enzyme was done for all atoms in implicit solvated environment at pH = 7, temperature = 300 K and salt concentration of 0.1. The complete structure was energy minimized using MMFF94X forcefield. The binding site of the enzyme was determined by selecting residues of catalytic triad i.e. Asp214, Glu276 and Asp349 (Represented as red spheres in Fig. S-2).

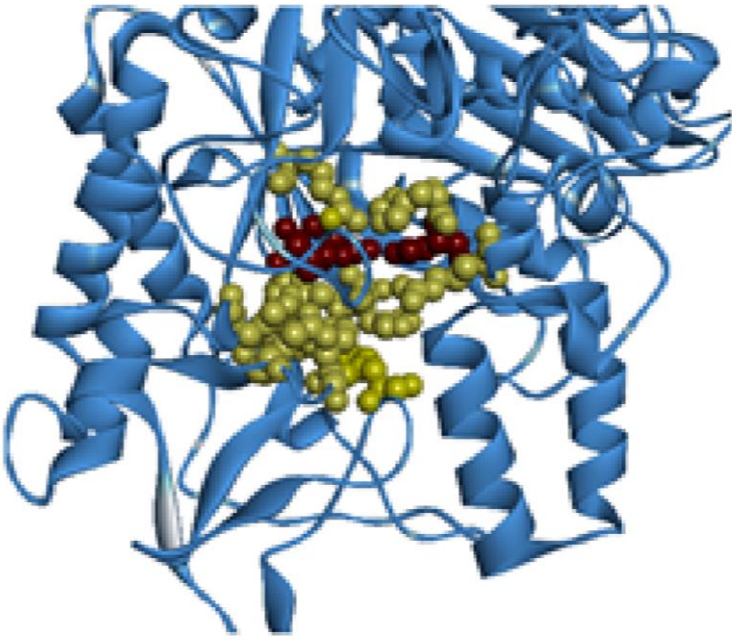

Figure S-2: A ribbon representation of homology modeled α-glucosidase. The three residues shown in red spheres are the residues of catalytic triad (Asp214, Glu276 and Asp349). The possible active site is shown in yellow spheres

Finally, all the compounds were docked into the binding sites of the prepared enzymes. Default docking parameters were set, and ten different conformations were generated for each compound. While performing docking, the ligand atom was selected and rescoring1 was set at London dG and rescoring2 at GBVI/WSA binding free energy. The ligands are ranked by the scores from the GBVI/WSA binding free energy calculation which is the score of the last stage. The GBVI/WSA is a scoring function which estimates the free energy of binding of the ligand from a given pose. Lowest binding energy ligand enzyme complexes were analyzed by MOE ligand interaction module. While, for 3-D interaction plot, discovery studio visualizer was used.

GC-MS spectra/details of the compounds

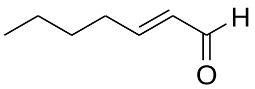

(A)

Compound Label	Name	*m/z*	RT	Algorithm
Cpd 4: 2-Heptenal, (E)- (CAS) $$ trans-2-Heptenal $$ (E)-2-Heptenal $$ 3-Butylacrolein	**2-Heptenal, (E)- (CAS) $$ trans-2-Heptenal $$ (E)-2-Heptenal $$ 3-Butylacrolein**	55,1	10,29	Find by Chromatogram Deconvolution

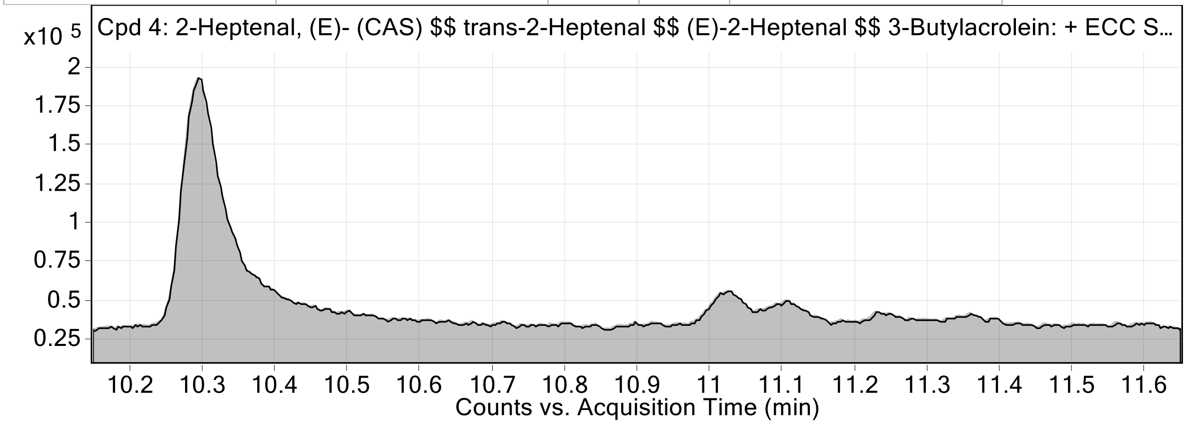

MS Spectrum

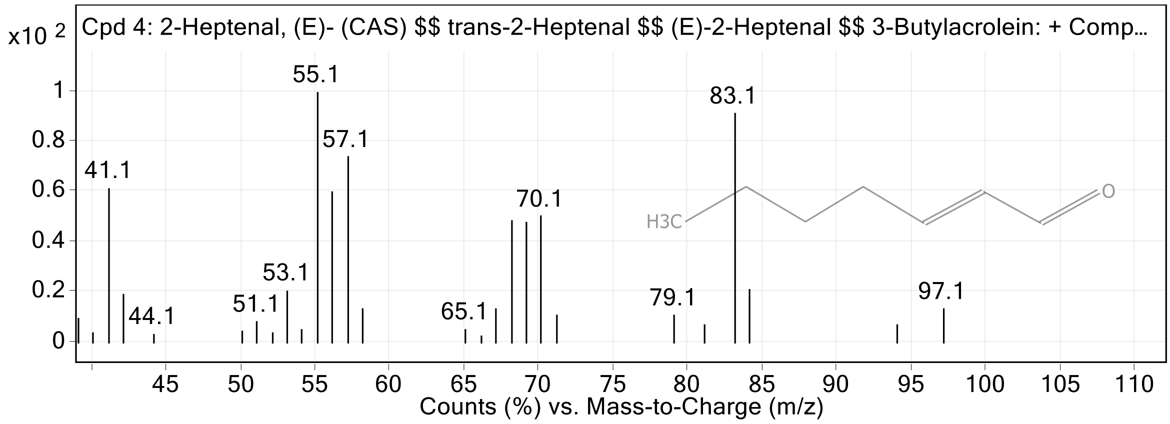

MS Zoomed spectrum

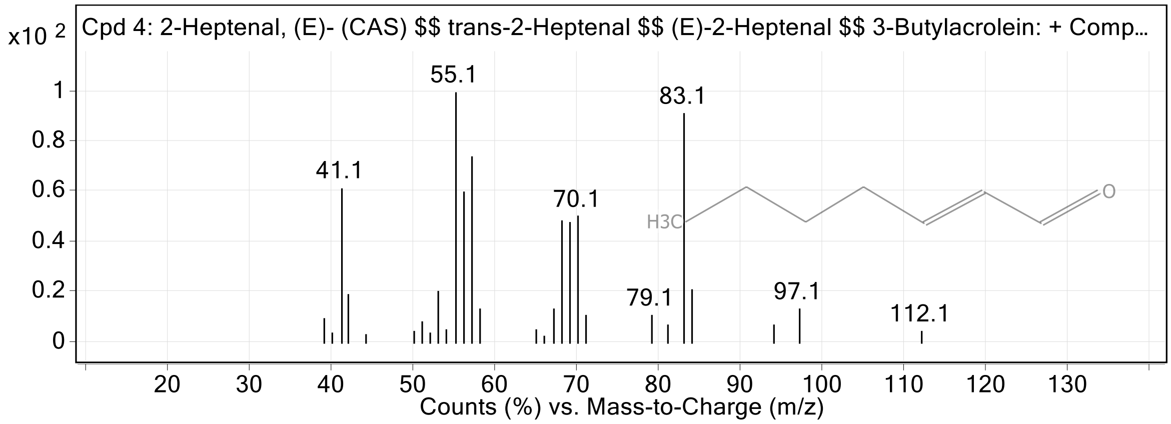

MS Spectrum Peak List

m/z	Abund
41,1	14152
53,1	4828,9
55,1	22873
56,1	13851
57,1	16971
68,1	11228
69,1	11023
70,1	11544
83,1	20929
84,1	4904,1

Library Spectrum

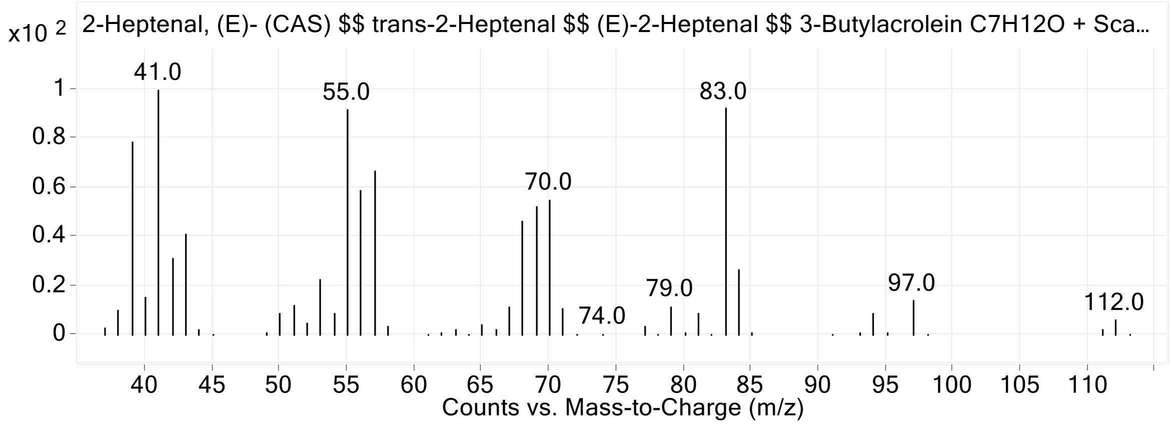

Difference Spectrum

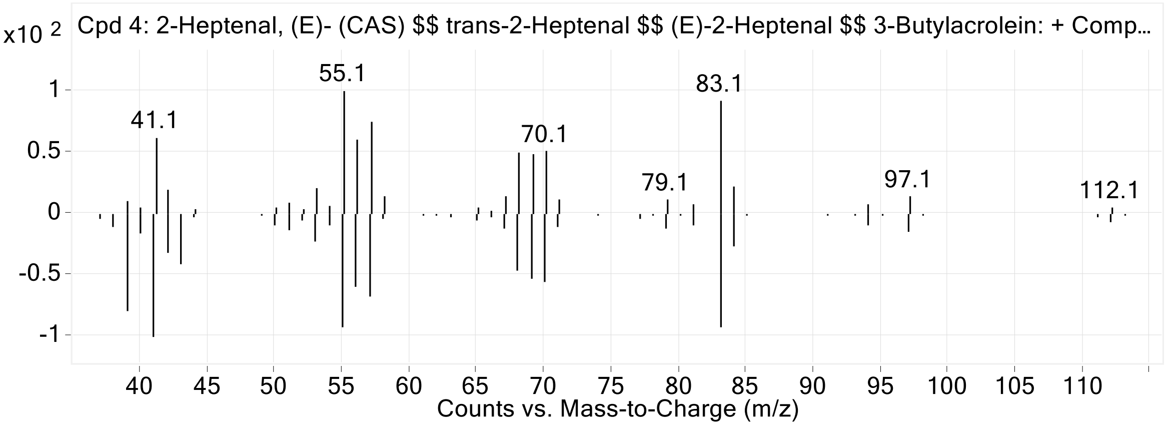

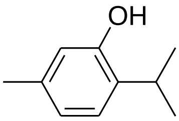

(B)

**Compound Label**	**Name**	***m/z***	**RT**	**Algorithm**
Cpd 6: Phenol, 5-methyl-2-(1-methylethyl)- (CAS) $$ Thymol (CAS) $$ m-Thymol	**Phenol, 5-methyl-2-(1-methylethyl)- (CAS) $$ Thymol (CAS) $$ m-Thymol**	135	23,56	Find by Chromatogram Deconvolution

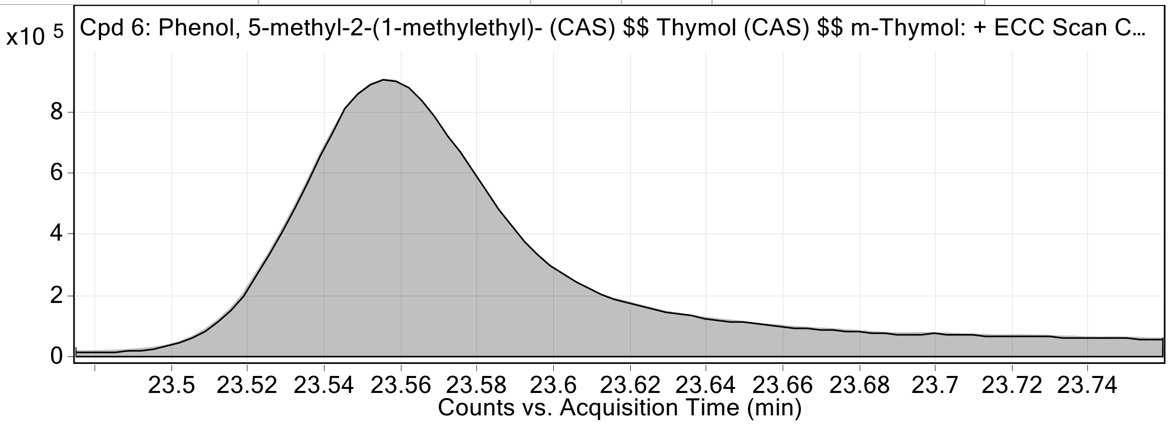

MS Spectrum

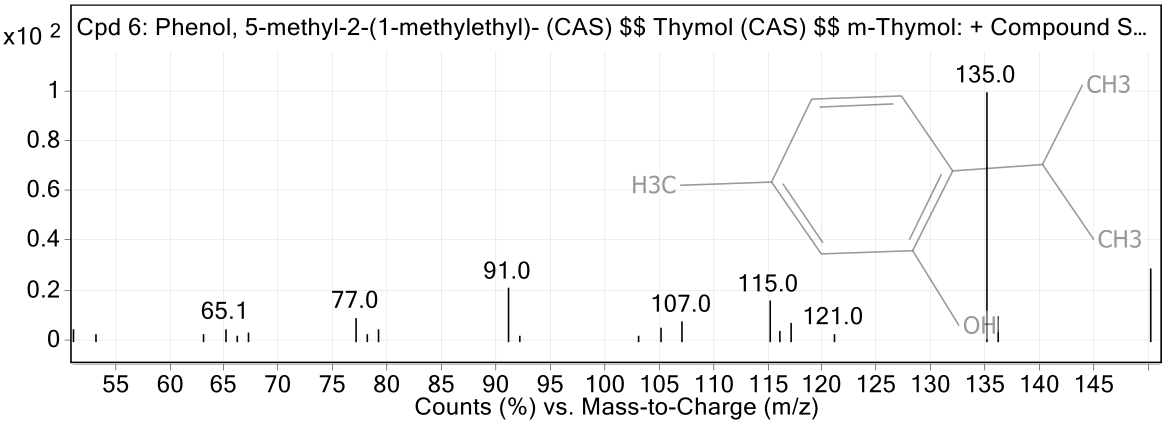

MS Zoomed Spectrum

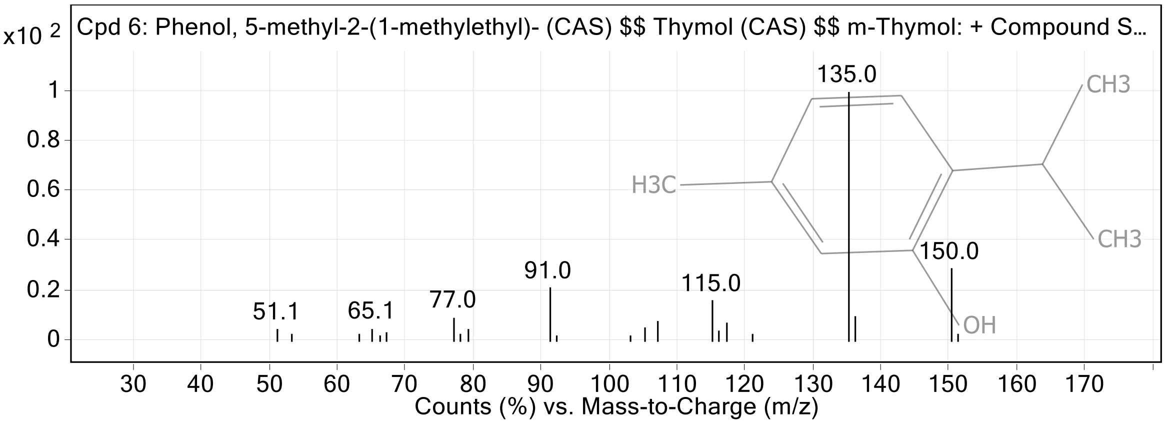

MS Spectrum Peak List

m/z	**Abund**
77	33764,1
79,1	18602,5
91	74758,3
105	19432,8
107	28343,3
115	56792,2
117	26322,6
135	350327,5
136	35888,3
150	101853,9

Library Spectrum

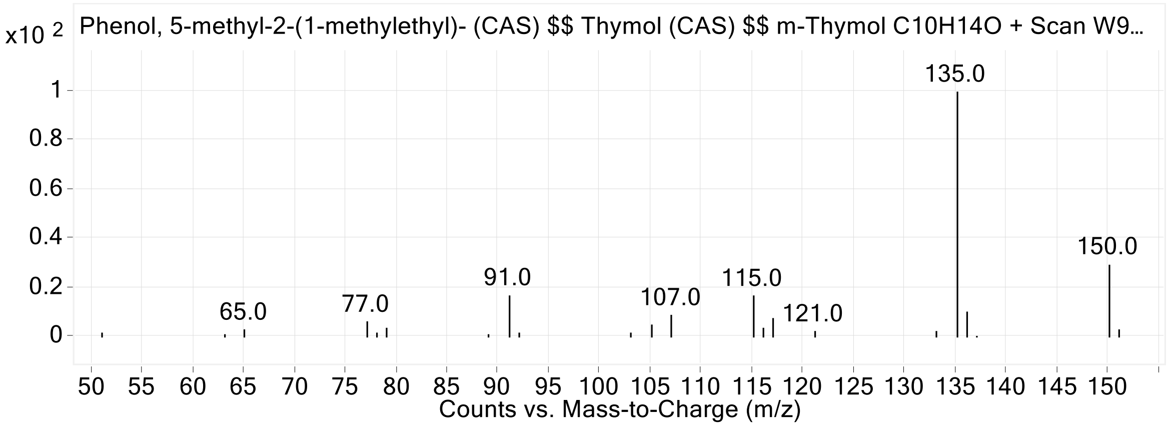

Difference Spectrum

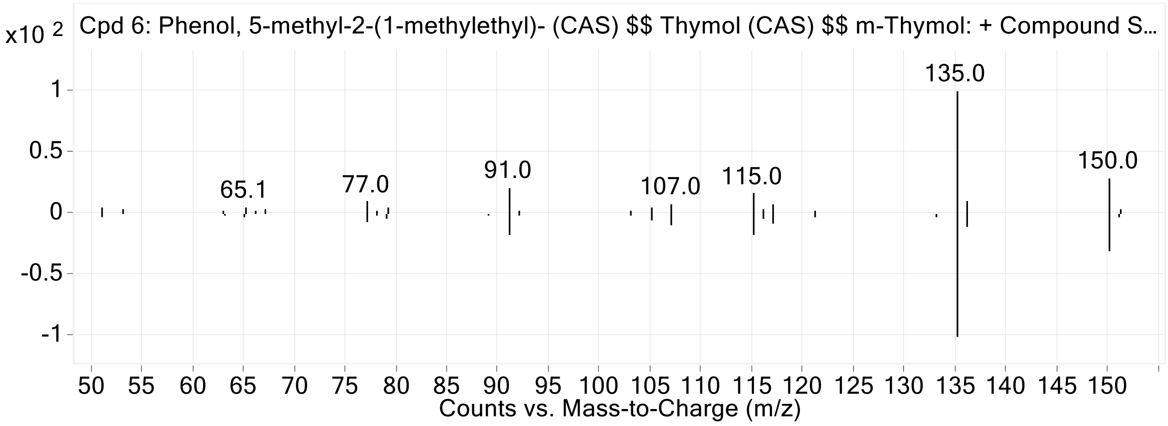

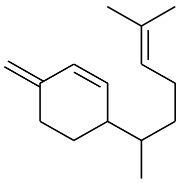

(C)

**Compound Label**	**Name**	***m/z***	**RT**	**Algorithm**
Cpd 8: Cyclohexene, 3-(1,5-dimethyl-4-hexenyl)-6-methylene-, [S-(R*,S*)]-	**Cyclohexene, 3-(1,5-dimethyl-4-hexenyl)-6-methylene-, [S-(R*,S*)]-**	69,1	30,71	Find by Chromatogram Deconvolution

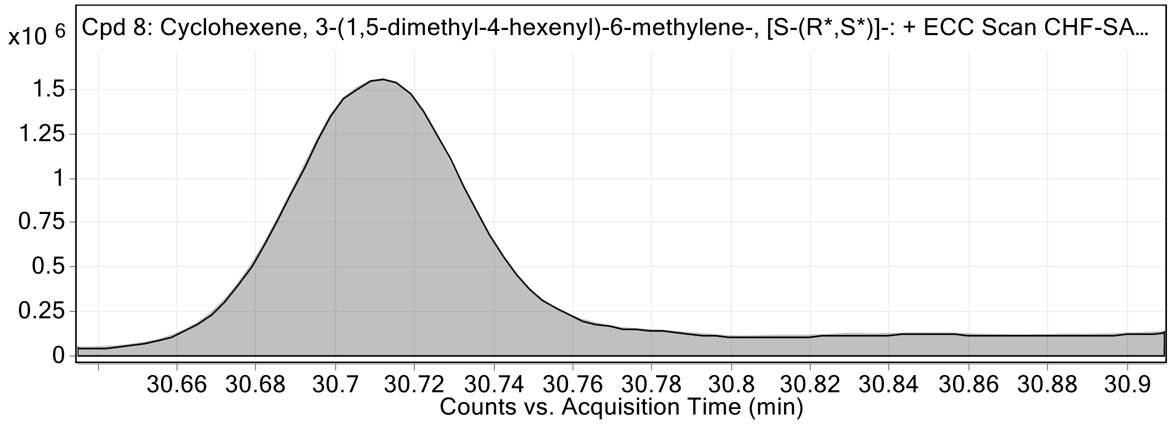

MS Spectrum

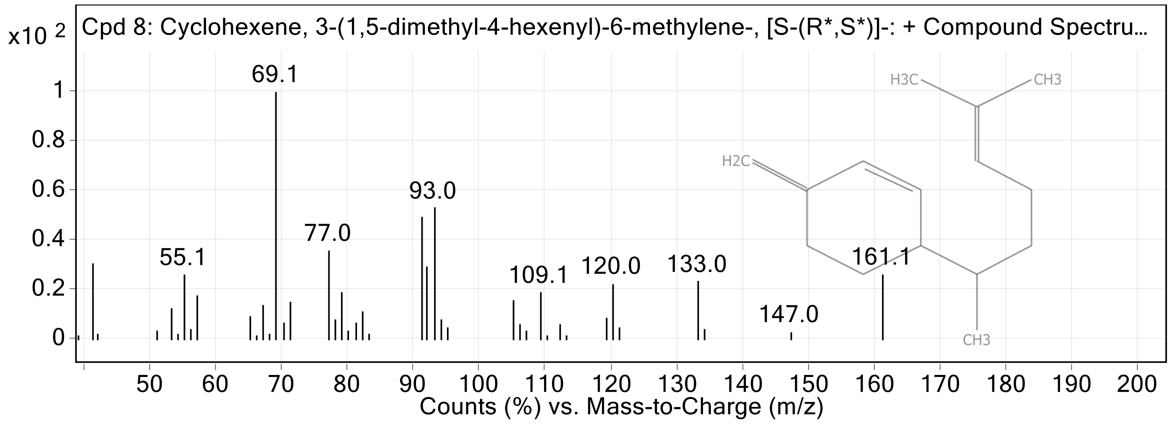

MS Zoomed Spectrum

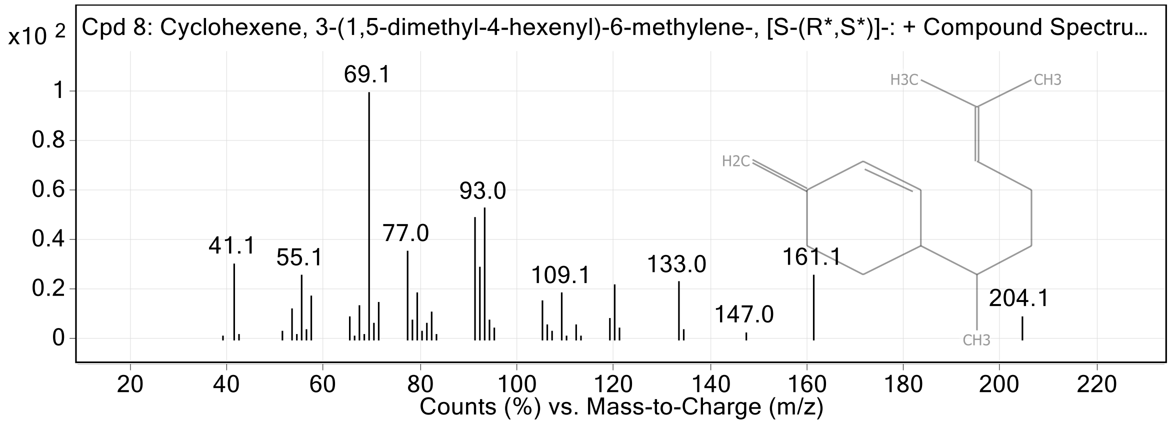

MS Spectrum Peak List

m/z	**Abund**
41,1	71306,4
55,1	60510,2
69,1	228473,6
77	82764,4

91	113773,5
92	68461,9
93	121576,6
120	52527,7
133	54203,6
161,1	60942,7

Library Spectrum

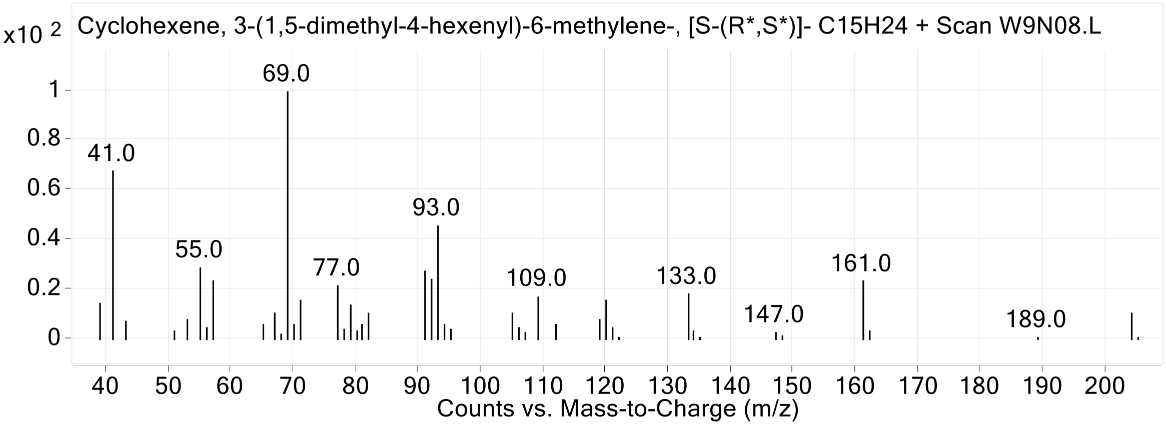

Difference Spectrum

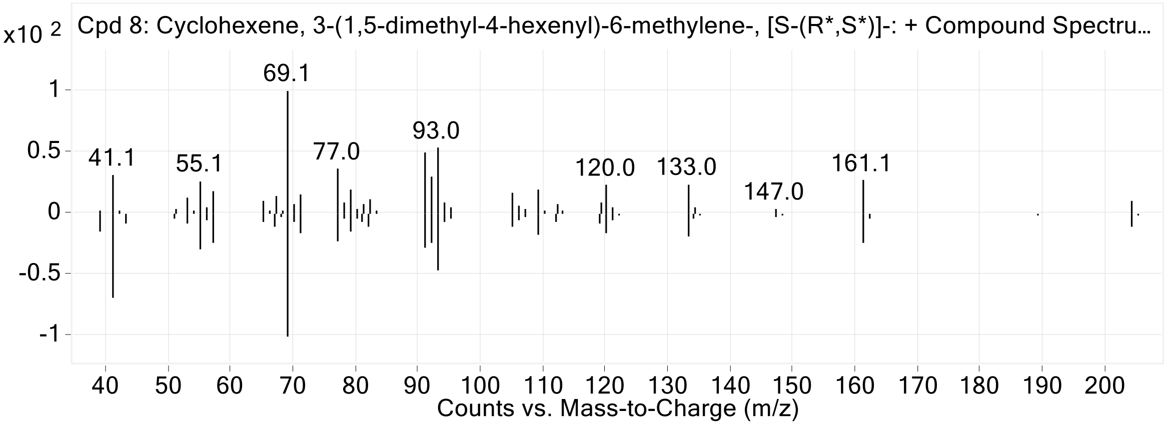

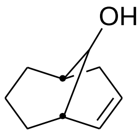

(D)

**Compound Label**	**Name**	***m/z***	**RT**	**Algorithm**

Cpd 10: Bicyclo[3.3.1]non-2-en-9-ol, syn- $$ syn-9-Hydroxybicyclo[3.3.1]Non-2-ene	**Bicyclo[3.3.1]non-2-en-9-ol, syn- $$ syn-9-Hydroxybicyclo[3.3.1]Non-2-ene**	120	34	Find by Chromatogram Deconvolution

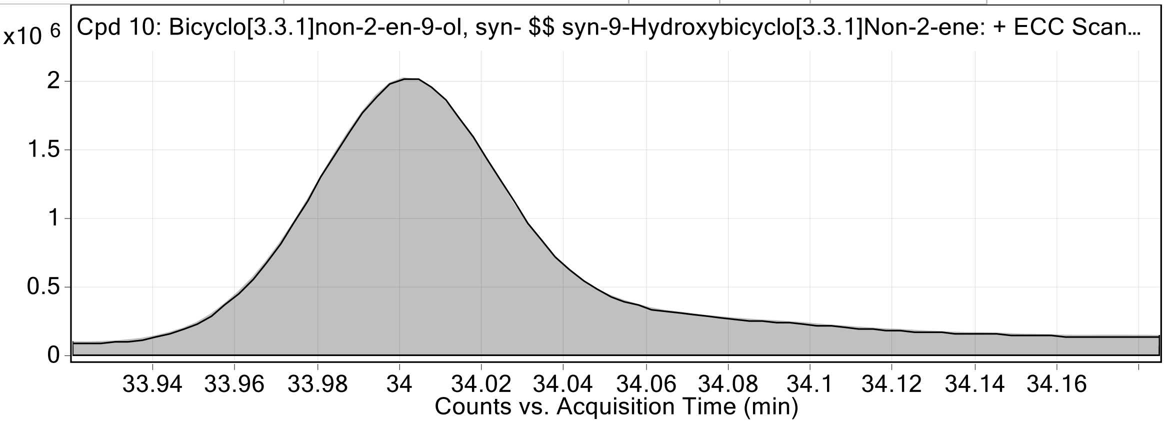

MS Spectrum

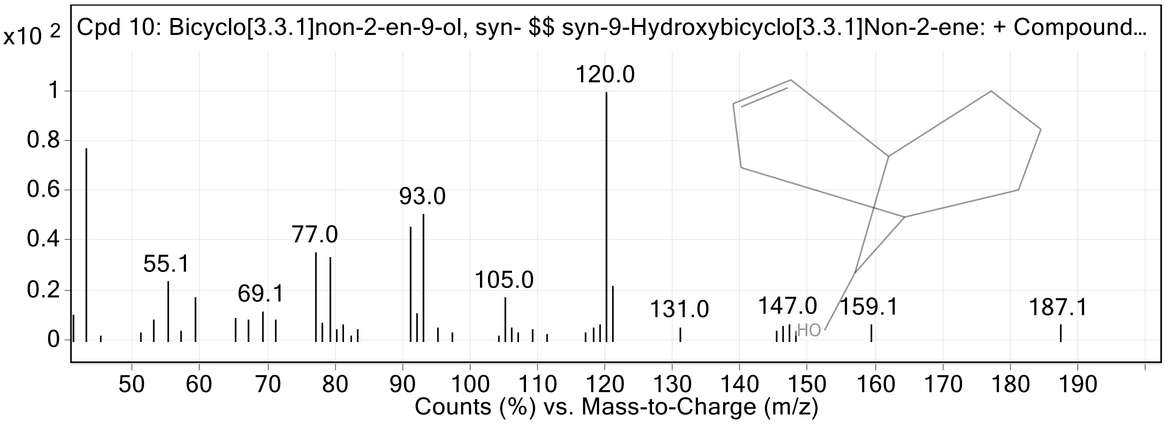

MS Zoomed Spectrum

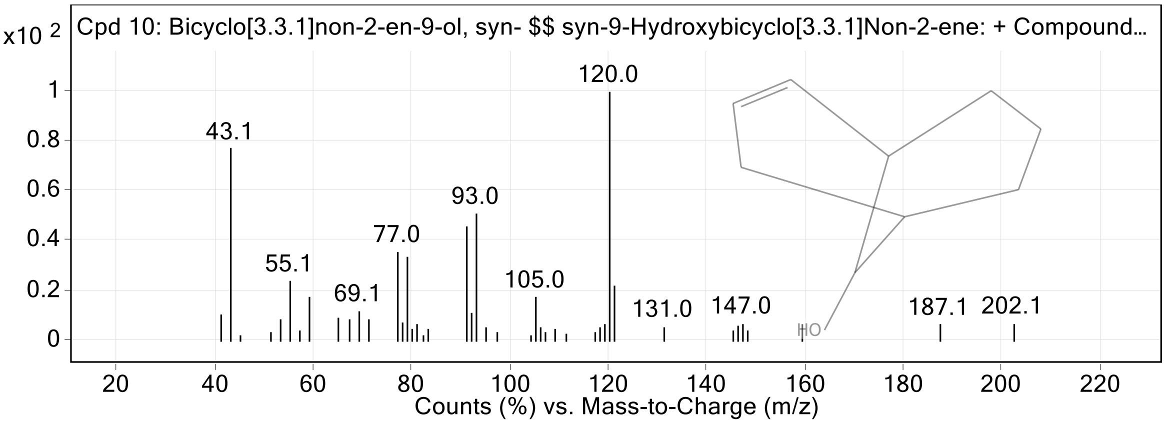

MS Spectrum Peak List

m/z	Abund
43,1	240896,8
55,1	75160
59,1	55744,1
77	112009,3
79,1	104662
91	142784,7
93	159946,7
105	55026,4
120	312821,9
121	68641,1

Library Spectrum

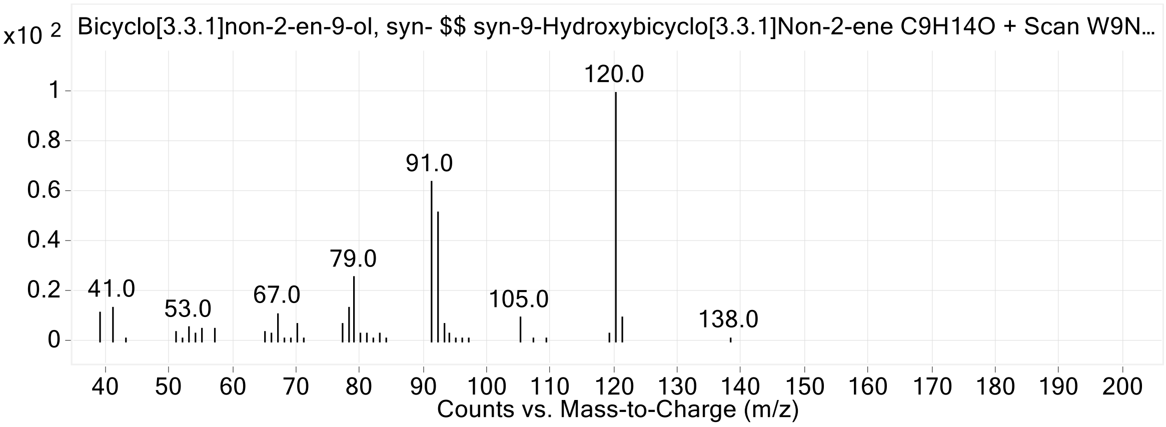

Difference Spectrum

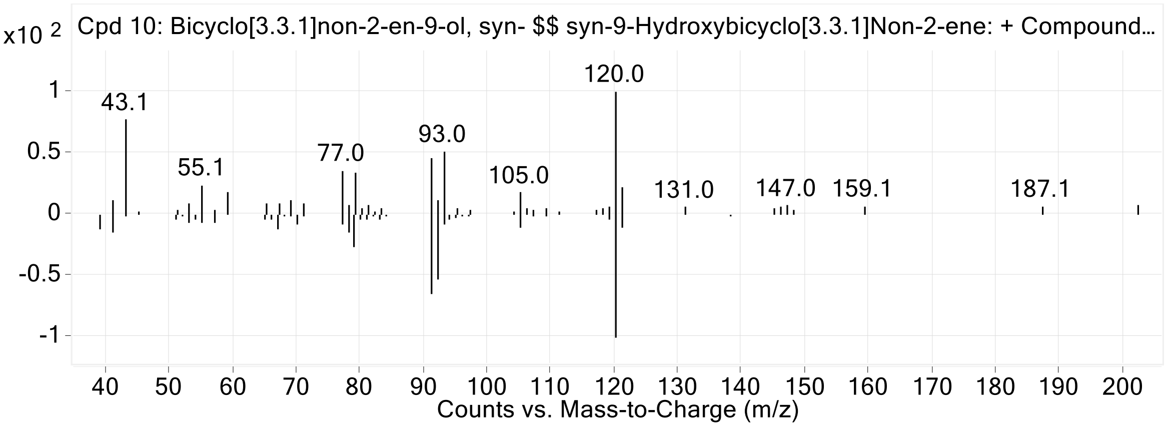

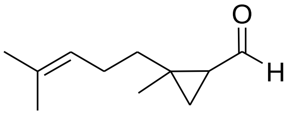

(E)

Compound Label	Name	*m/z*	RT	Algorithm

Cpd 14: Cyclopropanecarboxaldehyde, 2-methyl-2-(4-methyl-3-pentenyl)-, trans-(.+-.)-	**Cyclopropanecarboxaldehyde, 2-methyl-2-(4-methyl-3-pentenyl)-, trans-(.+-.)-**	69,1	35,5	Find by Chromatogram Deconvolution

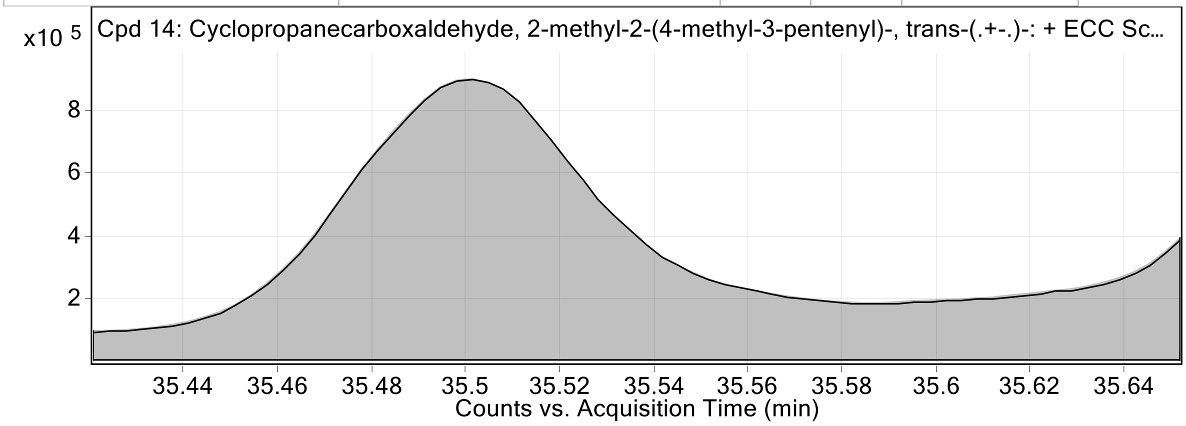

MS Spectrum

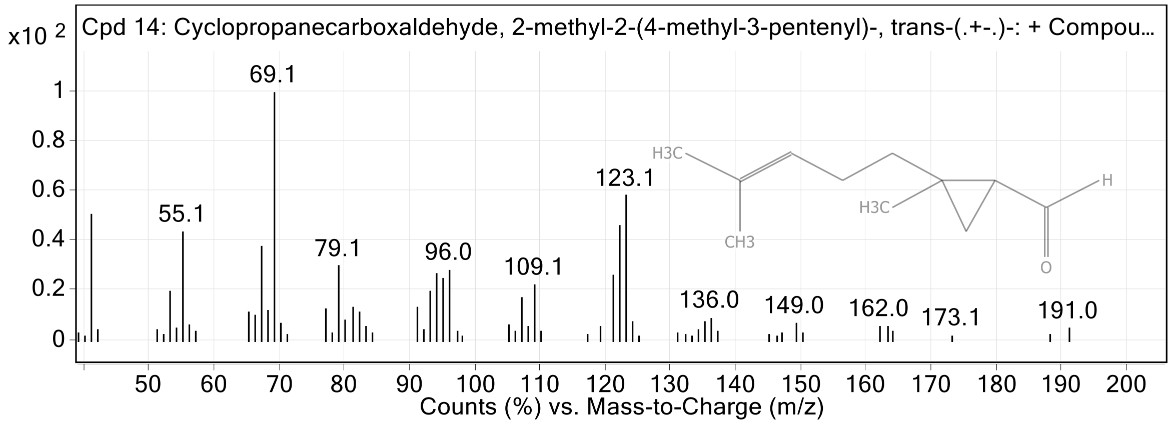

MS Zoomed Spectrum

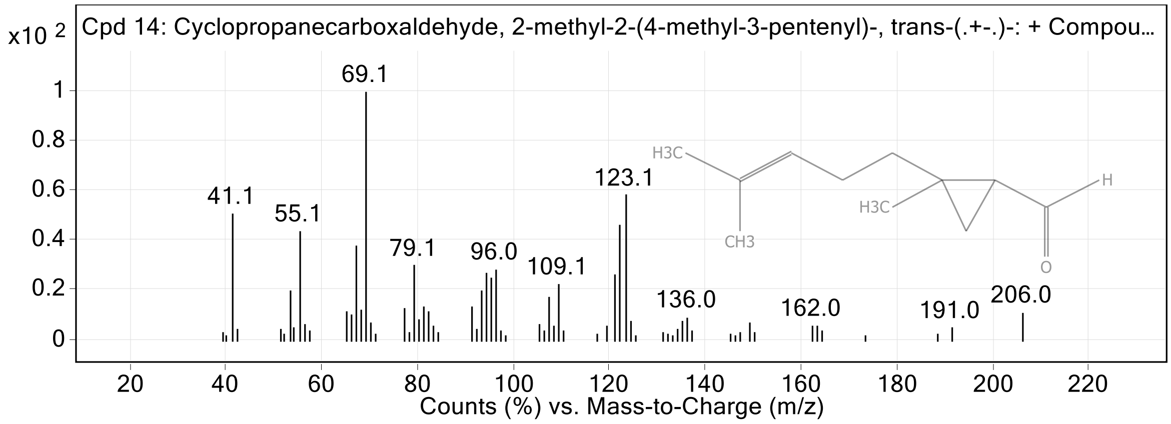

MS Spectrum Peak List

m/z	**Abund**
41,1	48496
55,1	41814
67,1	36477
69,1	94981
79,1	28660
94	25855
96	27294
121	25502
122	44322
123,1	55469

Library Spectrum

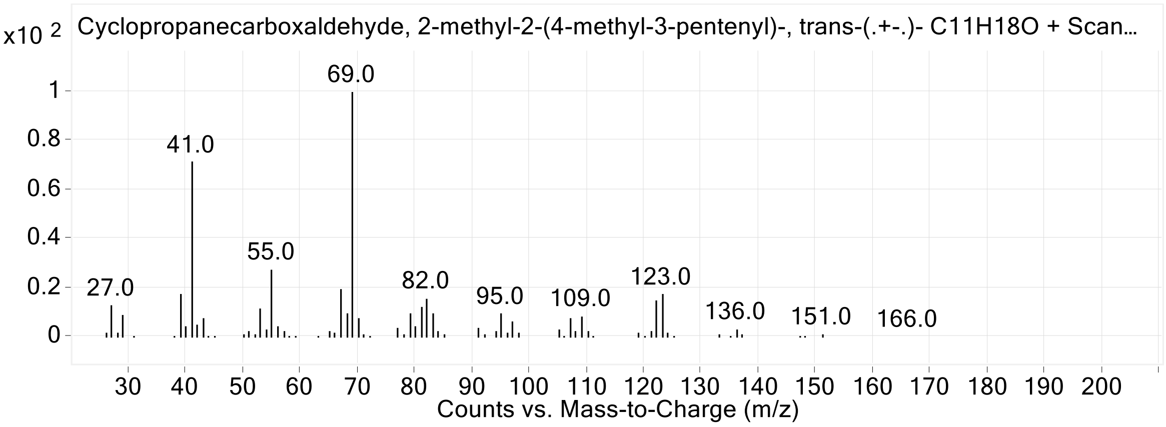

Difference Spectrum

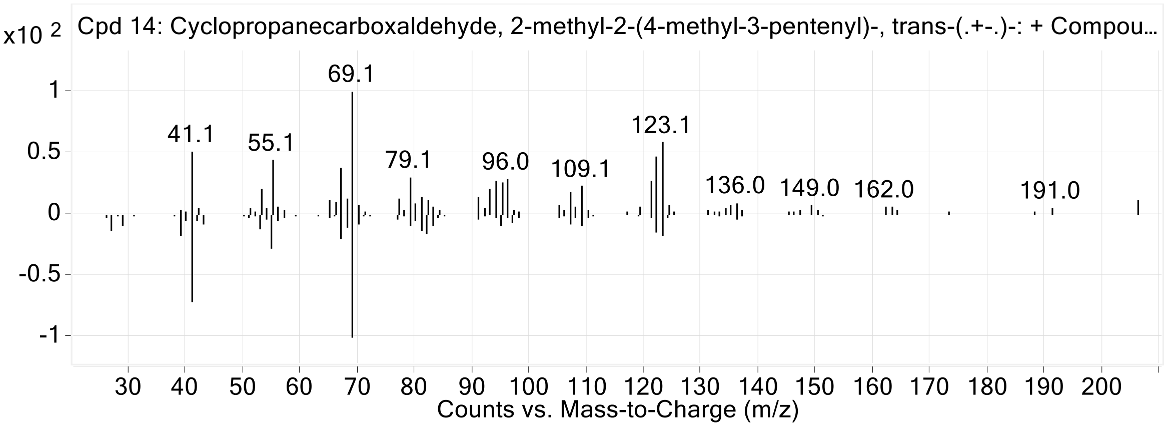

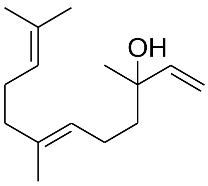

(F)

Compound Label	Name	m/z	RT	Algorithm
Cpd 15: Nerolidol $$ 1,6,10-Dodecatrien-3-ol, 3,7,11-trimethyl- (CAS) $$ E-farnesol	**Nerolidol $$ 1,6,10-Dodecatrien-3-ol, 3,7,11-trimethyl- (CAS) $$ E-farnesol**	69,1	35,7	Find by Chromatogram Deconvolution

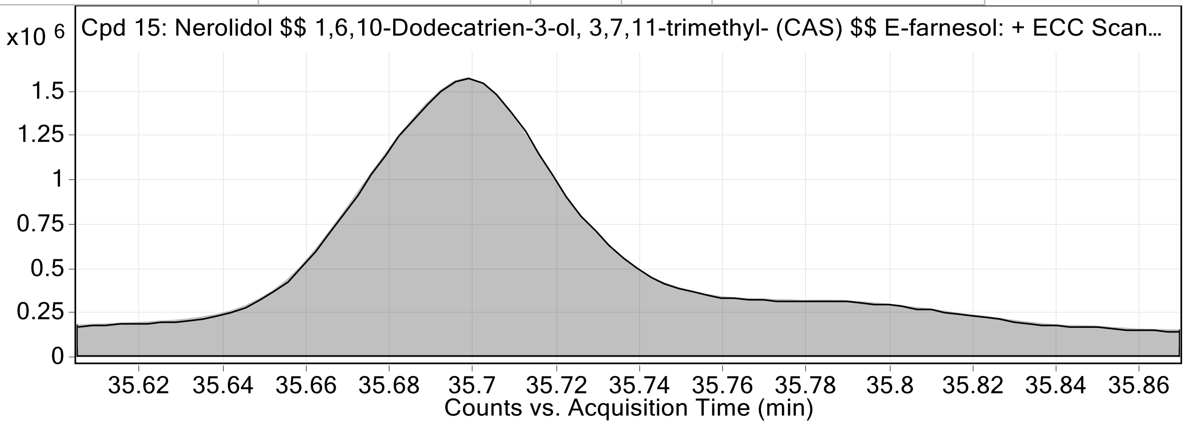

MS Spectrum

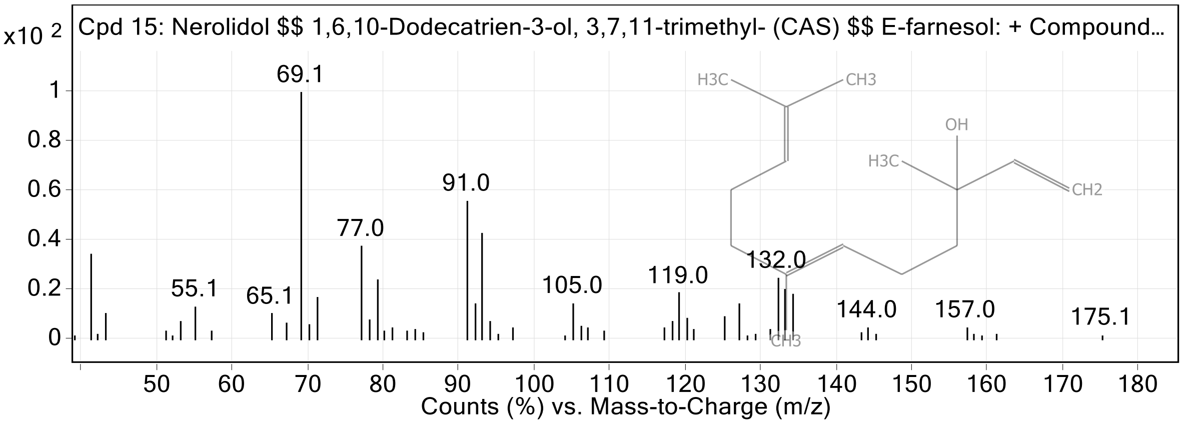

MS Zoomed Spectrum

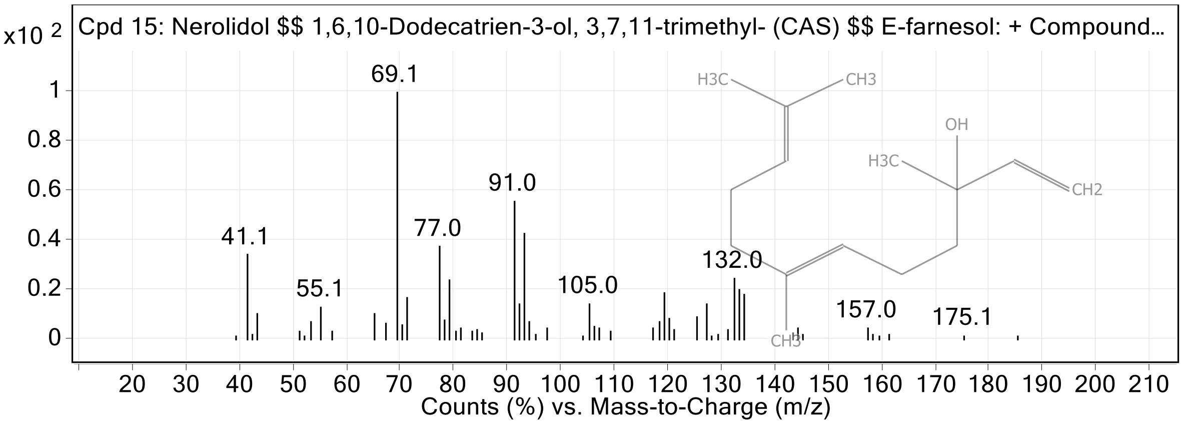

MS Spectrum Peak List

m/z	**Abund**
41,1	77966,2
69,1	224238,1
77	85583,1
79,1	55644,4
91	126388,2
93	97008,9
119	42999,5
132	56857,2
133	47286,7
134	42601,5

Library Spectrum

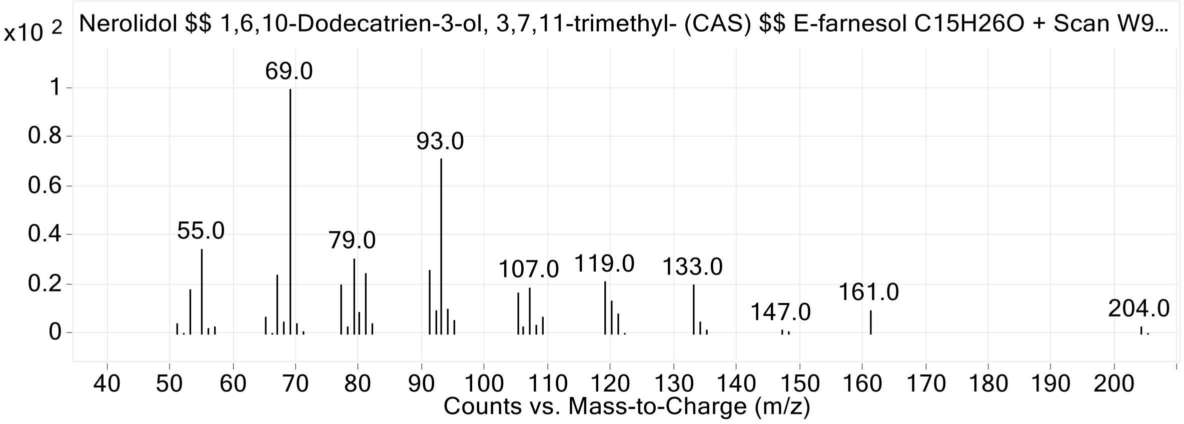

Difference Spectrum

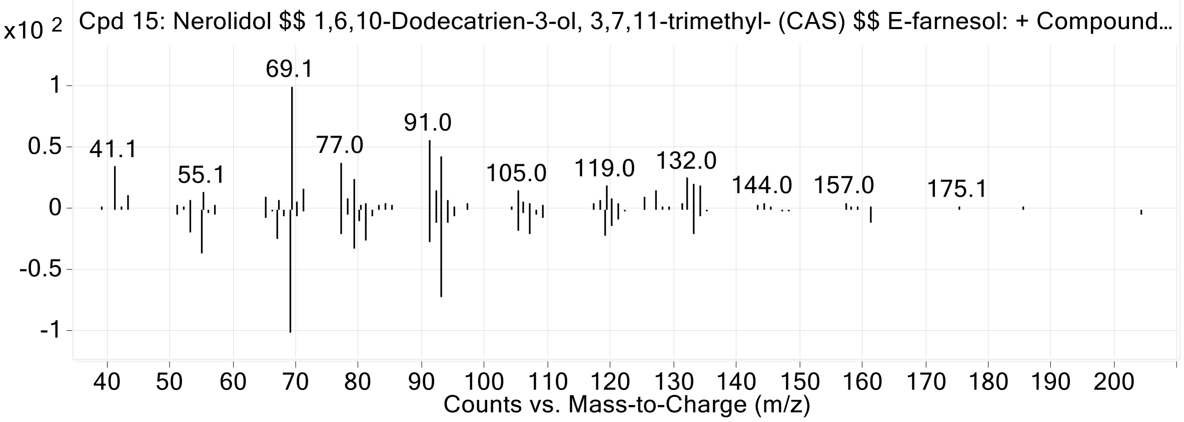

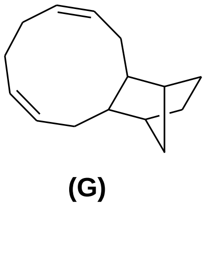

(G)

Compound Label	Name	*m/z*	RT	Algorithm
Cpd 16: 1,4-Methanobenzocyclodecene, 1,2,3,4,4a,5,8,9,12,12a-decahydro-	**1,4-Methanobenzocyclodecene, 1,2,3,4,4a,5,8,9,12,12a-decahydro-**	91	35,96	Find by Chromatogram Deconvolution

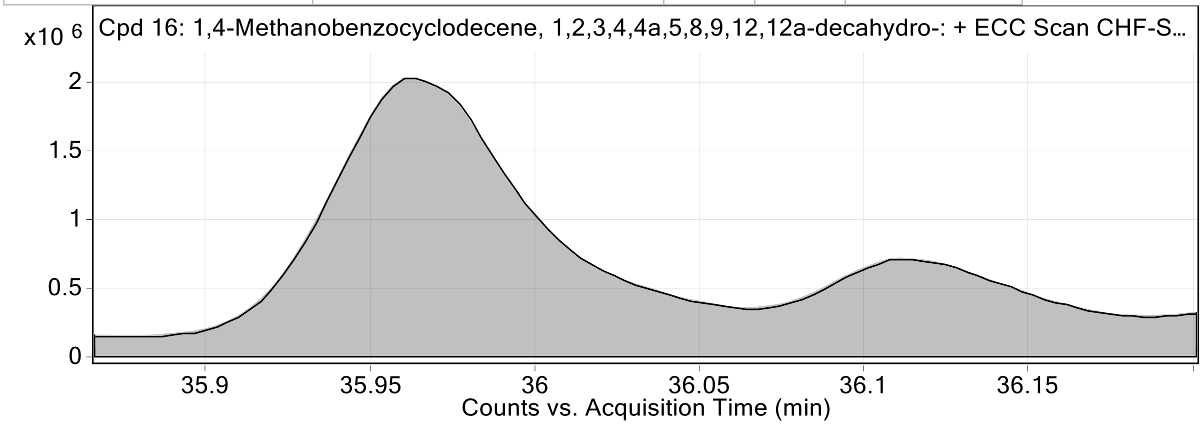

MS Spectrum

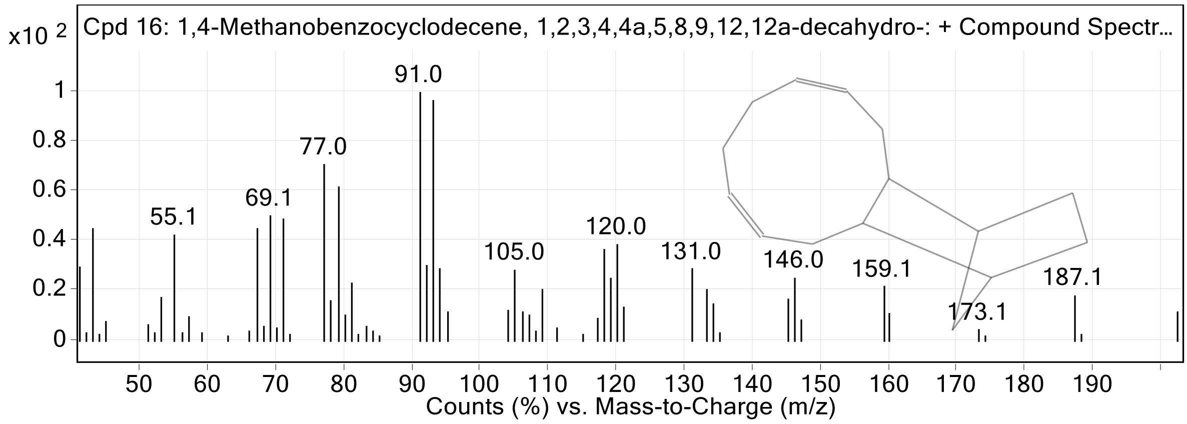

MS Zoomed Spectrum

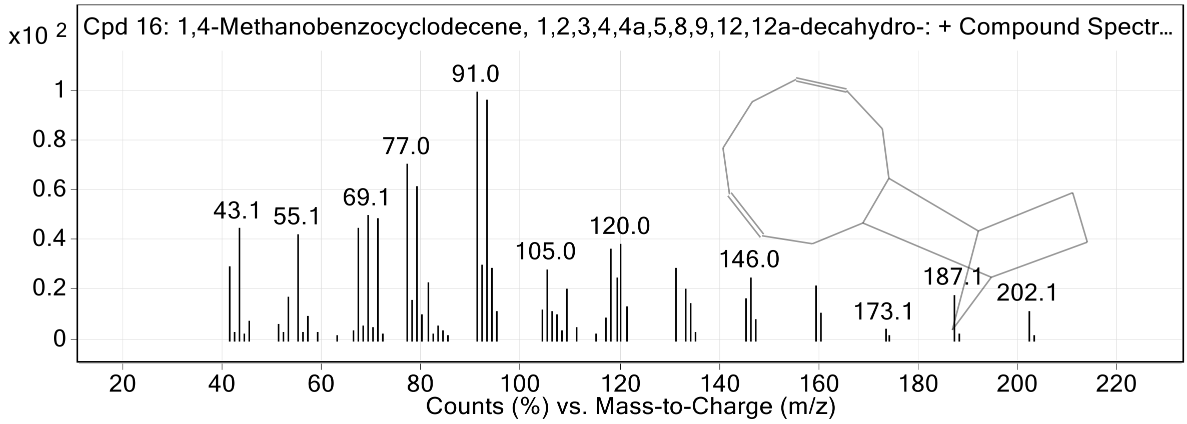

MS Spectrum Peak List

m/z	Abund
43,1	70906,1
55,1	67226,9
67,1	70558,7
69,1	79180,6
71,1	77410,6
77	111470,3
79,1	97427,7
91	156987,7
93	151154,8
120	61177

Library Spectrum

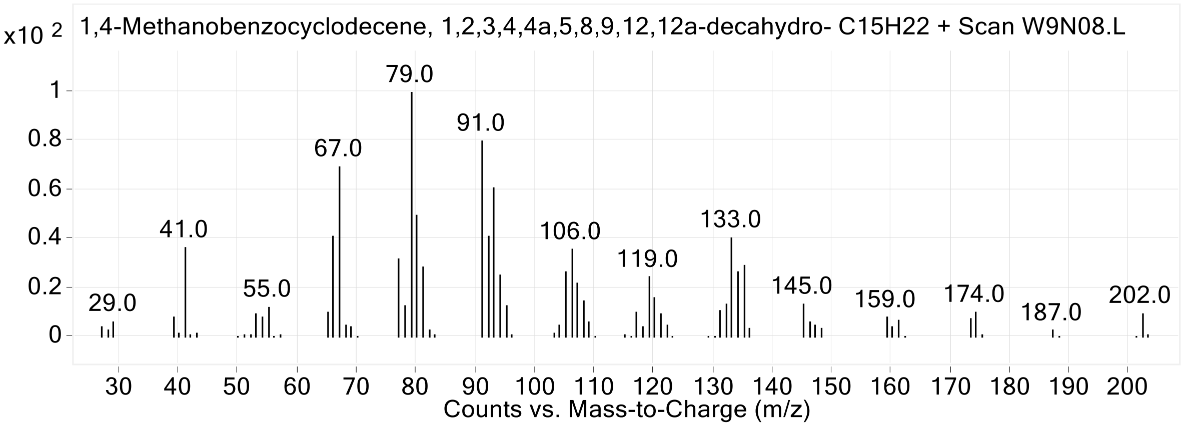

Difference Spectrum

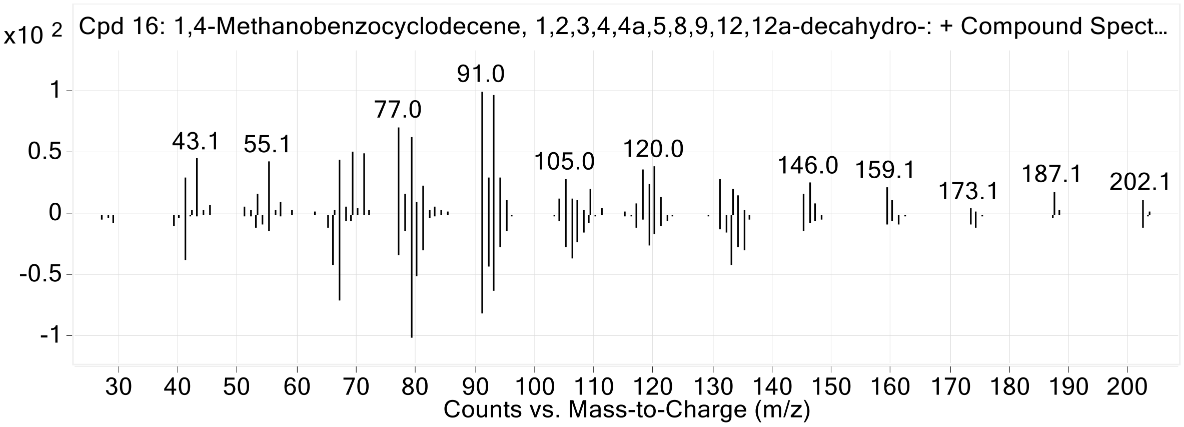

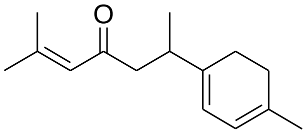

(H)

Compound Label	Name	*m/z*	RT	Algorithm

Cpd 21: (+)-.alpha.-Atlantone	**(+)-.alpha.-Atlantone**	83,1	37,81	Find by Chromatogram Deconvolution

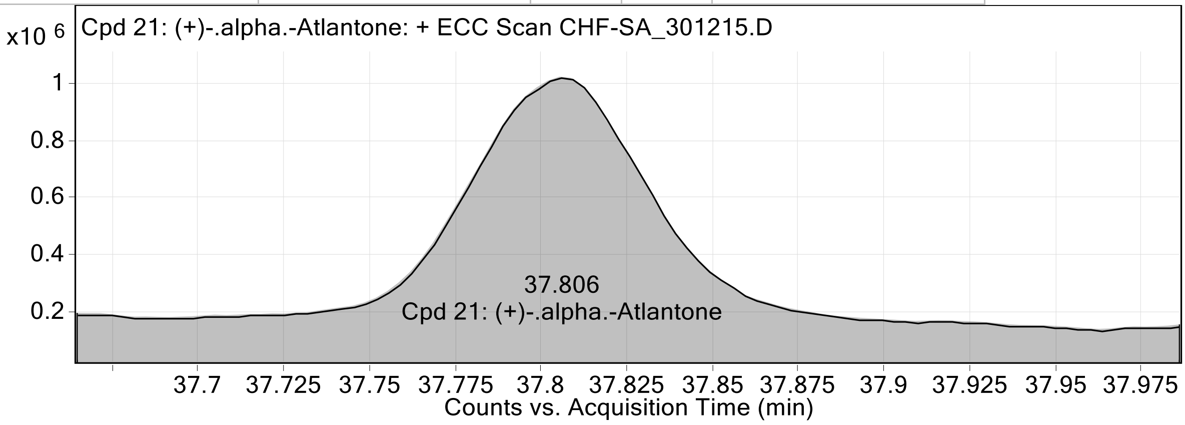

MS Spectrum

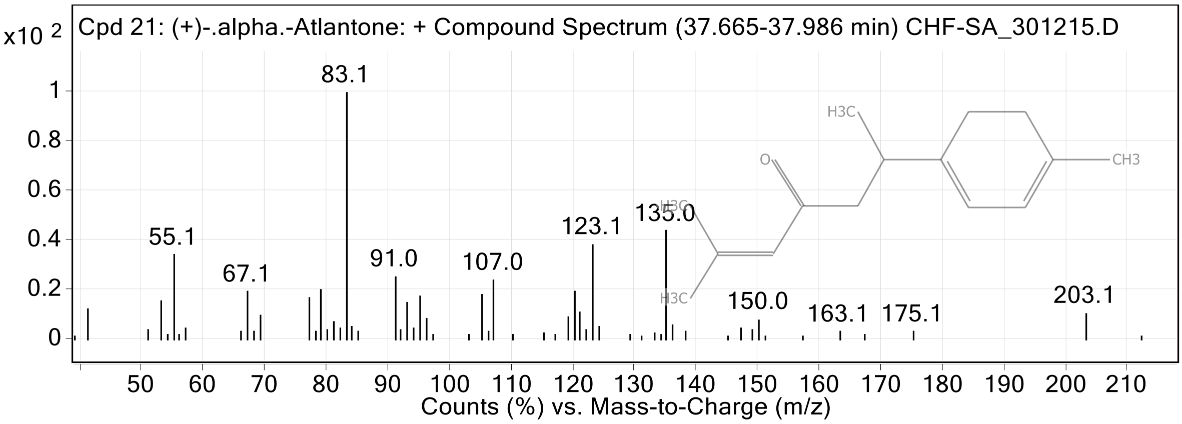

MS Zoomed Spectrum

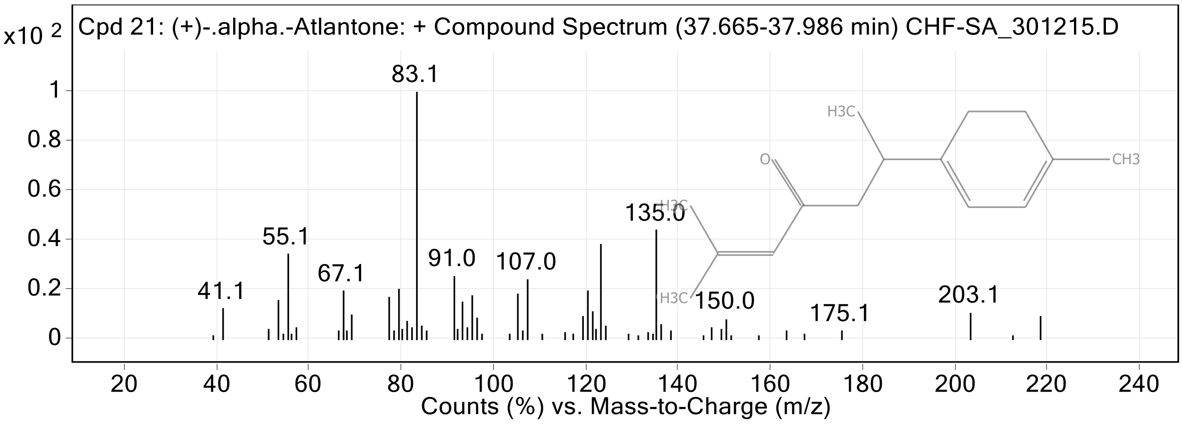

MS Spectrum Peak List

m/z	Abund
55,1	49063,9
67,1	28138,4

79,1	29566,3
83,1	141343,4
91	36782,8
105	26411,9
107	34812
120	28648,1
123,1	55074,2
135	62542,9

Library Spectrum

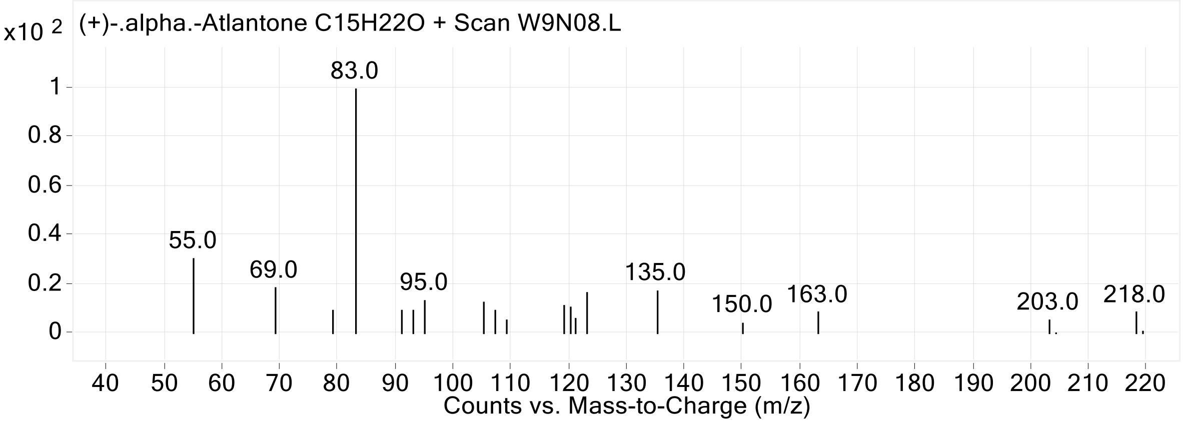

Difference Spectrum

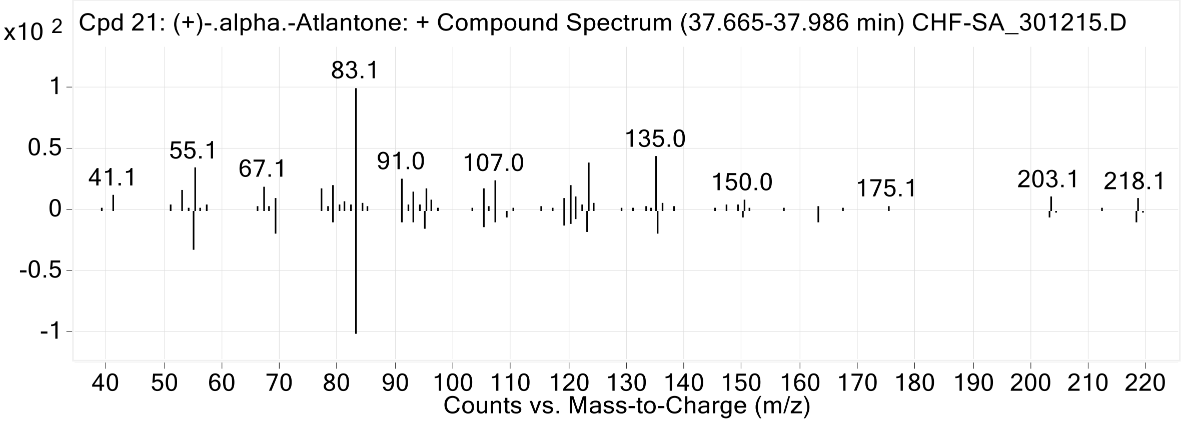

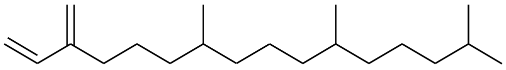

(I)

**Compound Label**	**Name**	***m/z***	**RT**	**Algorithm**

Cpd 24: Neophytadiene $$ 7,11,15-TRIMETHYL,3-METHYLENE-1-HEXADECENE	**Neophytadiene $$ 7,11,15-TRIMETHYL,3-METHYLENE-1-HEXADECENE**	68,1	39,41	Find by Chromatogram Deconvolution

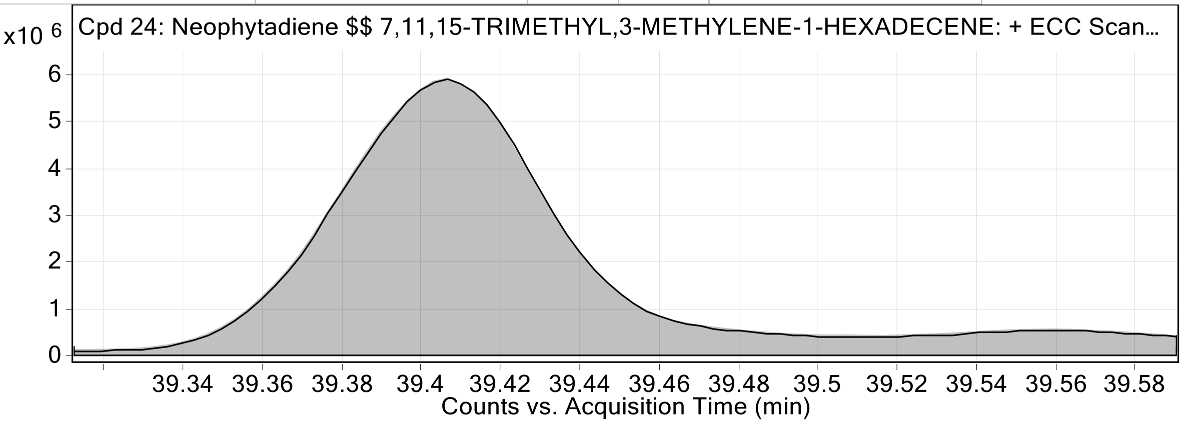

MS Spectrum

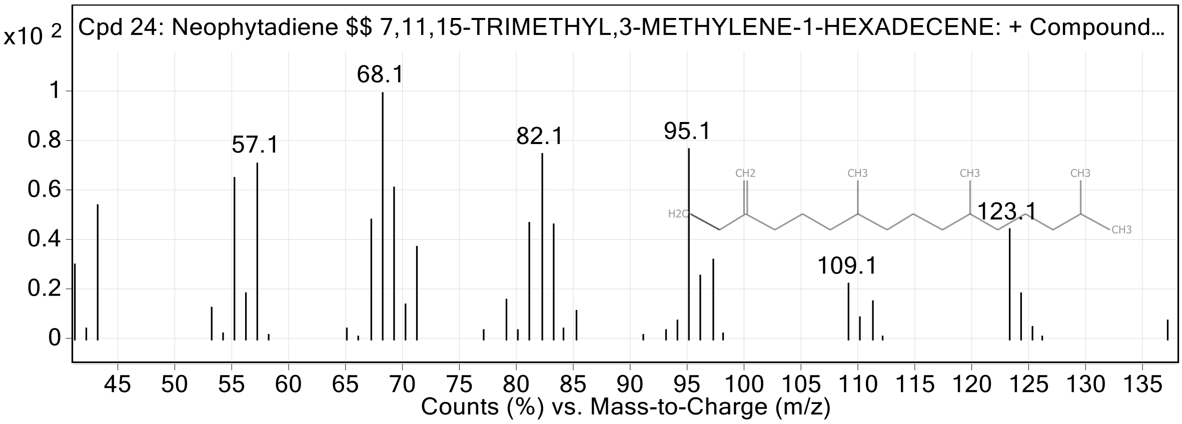

MS Zoomed Spectrum

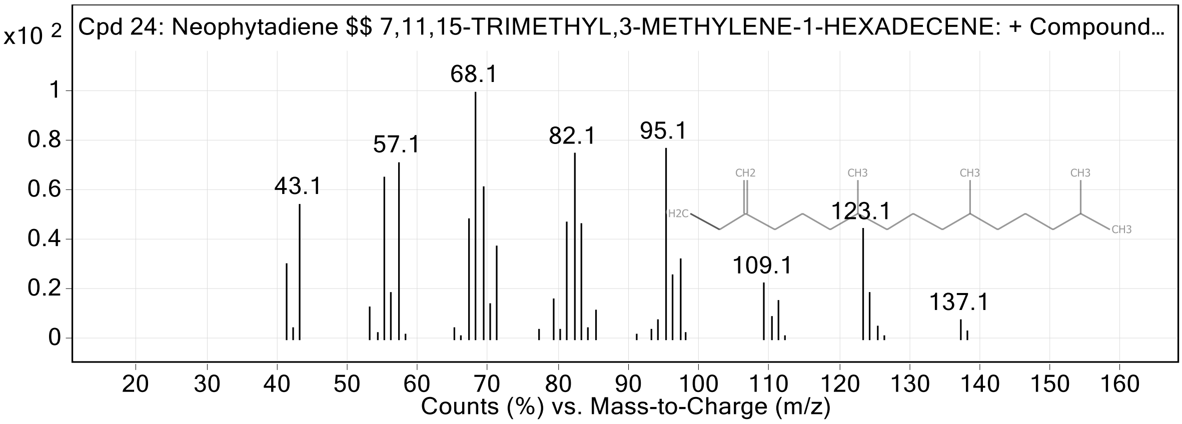

MS Spectrum Peak List

m/z	Abund
43,1	304922,6
55,1	363252,9
57,1	397858,1
67,1	272754
68,1	554584
69,1	343178,1
81,1	265855,7
82,1	416965,9
83,1	260153,3
95,1	428246,1

Library Spectrum

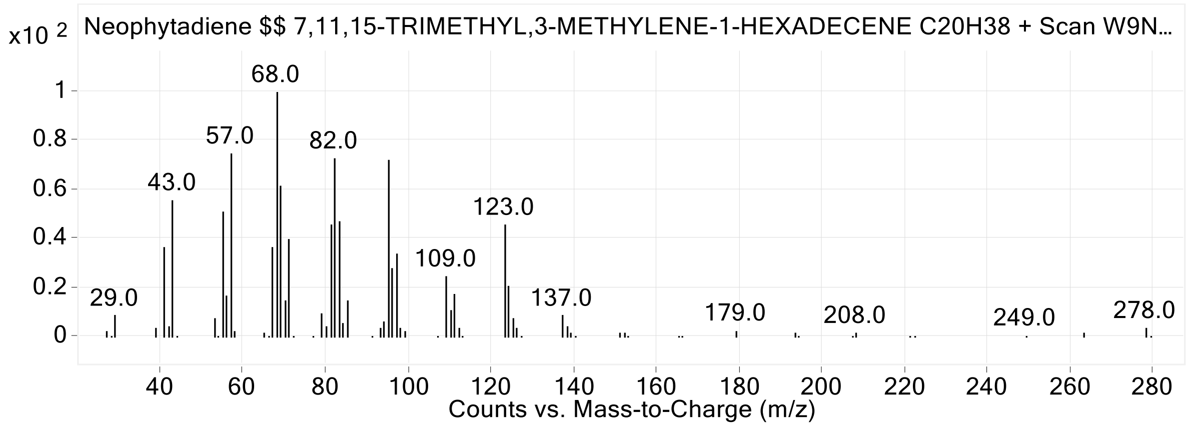

Difference Spectrum

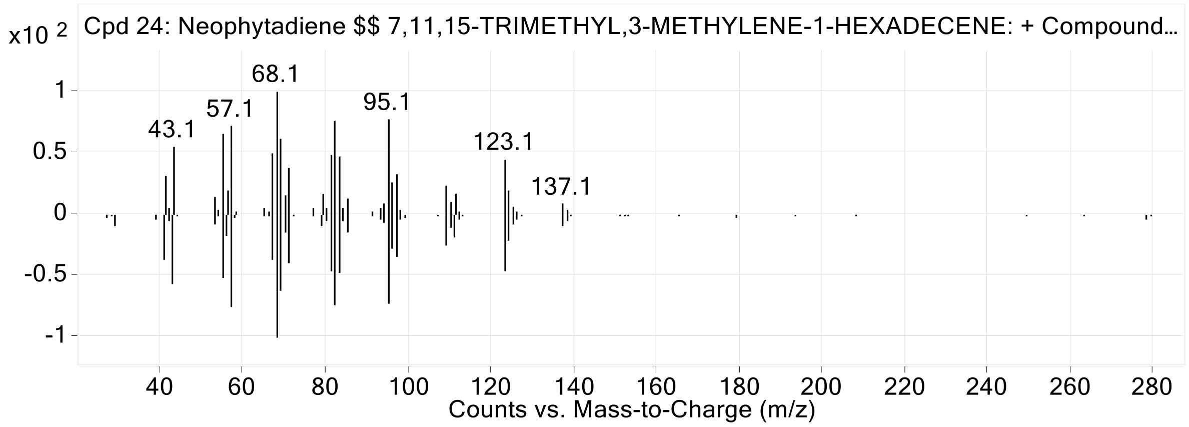

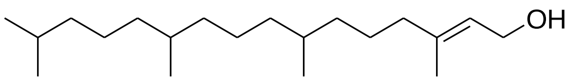

(J)

Compound Label	Name	*m/z*	RT	Algorithm

Cpd 47: Phytol $$ 2-Hexadecen-1-ol, 3,7,11,15-tetramethyl-, [R-[R*,R*-(E)]]-	**Phytol $$ 2-Hexadecen-1-ol, 3,7,11,15-tetramethyl-, [R-[R*,R*-(E)]]-**	71,1	49,43	Find by Chromatogram Deconvolution

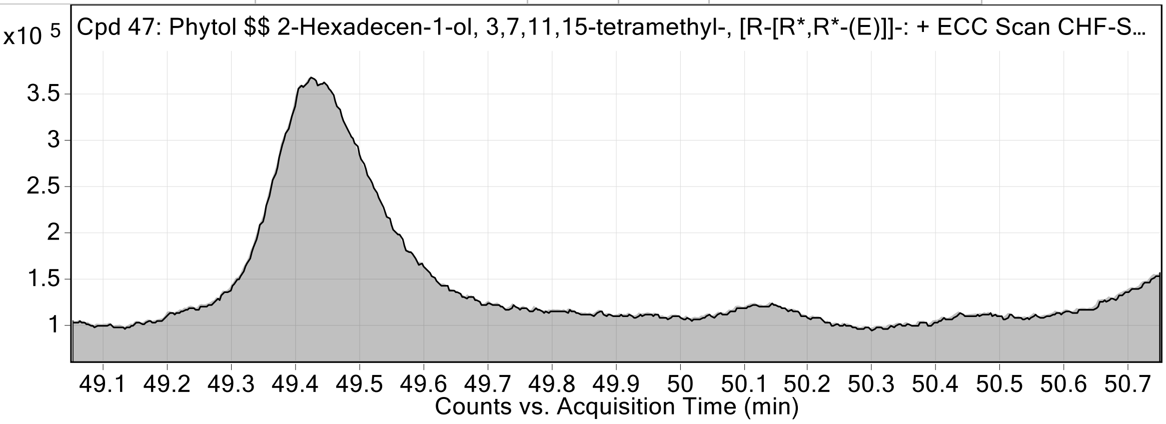

MS Spectrum

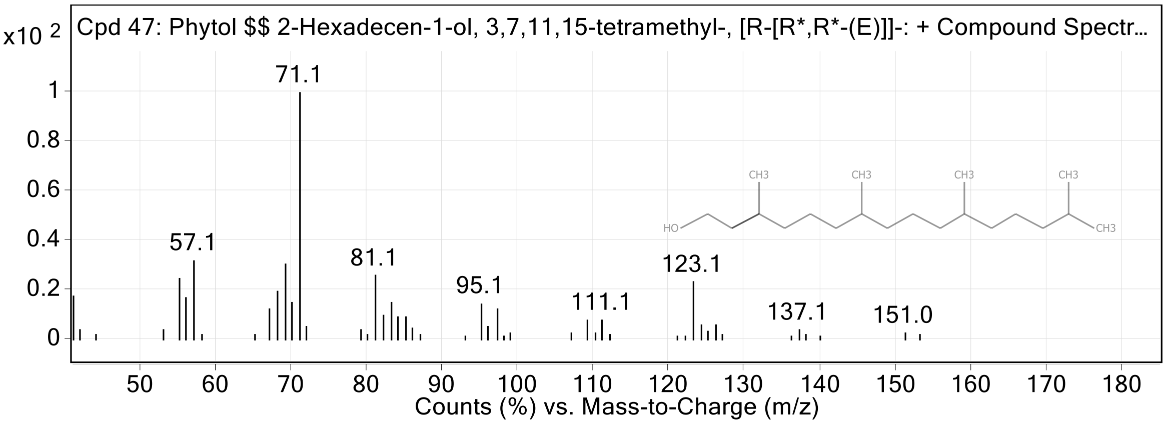

MS Zoomed Spectrum

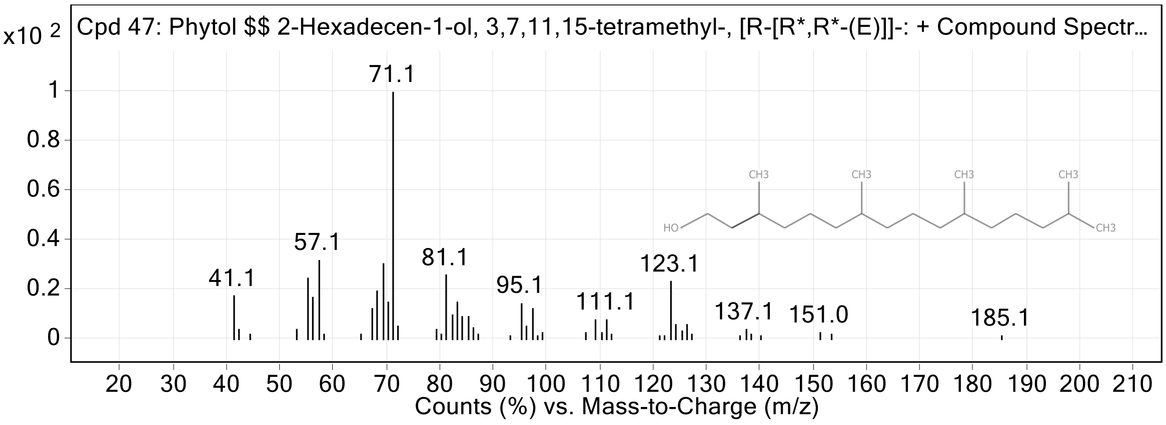

MS Spectrum Peak List

m/z	Abund
41,1	9842,2
55,1	13950
56,1	9470,4
57,1	17788
68,1	11119
69,1	16862
70,1	8725,7
71,1	54735
81,1	14412
123,1	13017

Library Spectrum

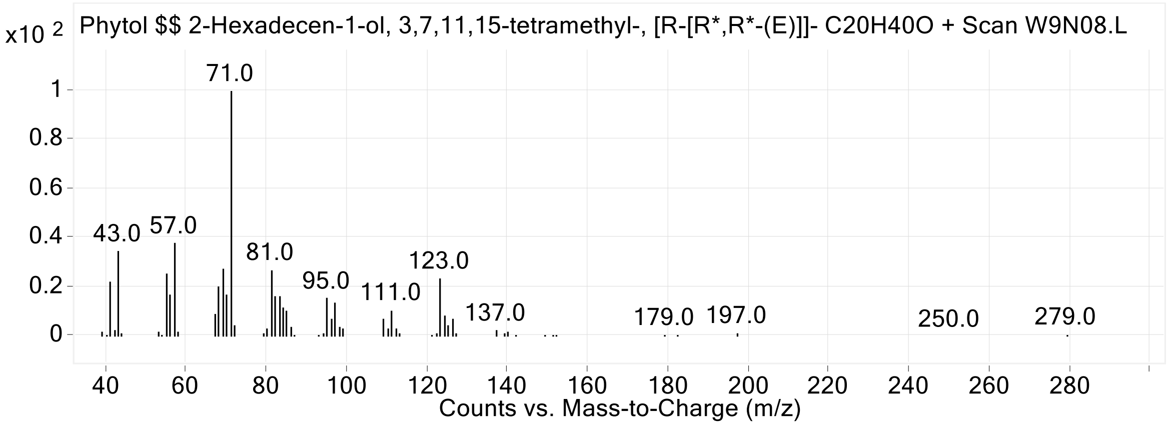

Difference Spectrum

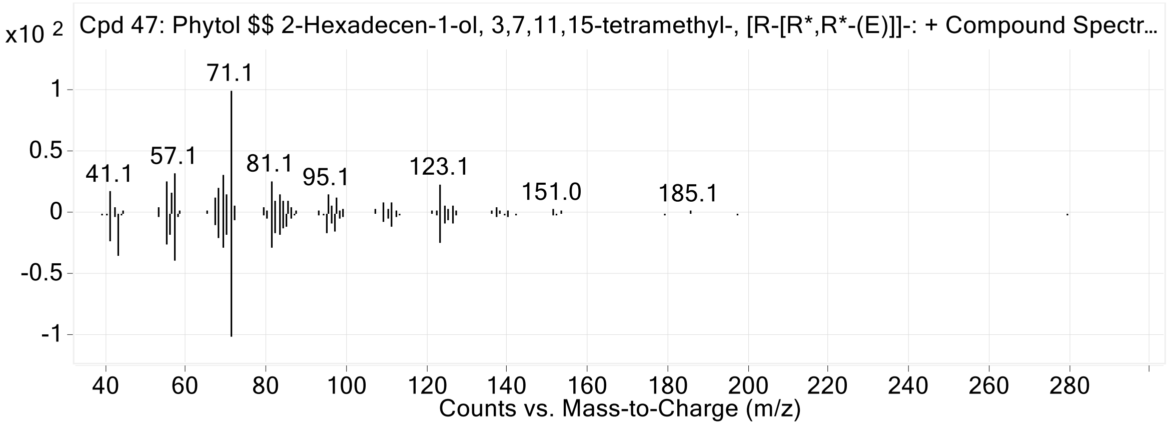

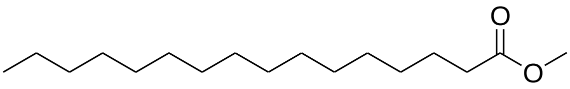

(K)

Compound Label	Name	*m/z*	RT	Algorithm

Cpd 35: Hexadecanoic acid, methyl ester (CAS) $$ Methyl palmitate $$ Uniphat A60	**Hexadecanoic acid, methyl ester (CAS) $$ Methyl palmitate $$ Uniphat A60**	74	41,73	Find by Chromatogram Deconvolution

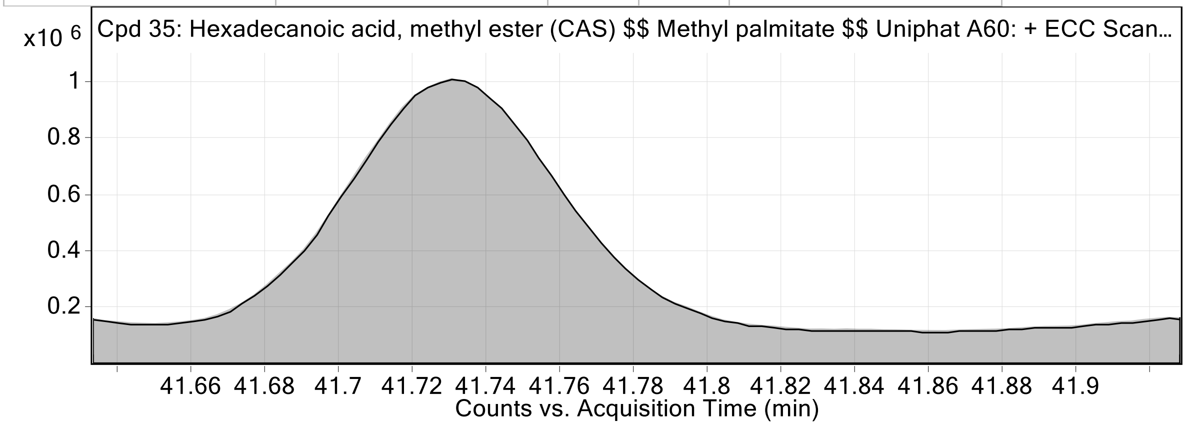

MS Spectrum

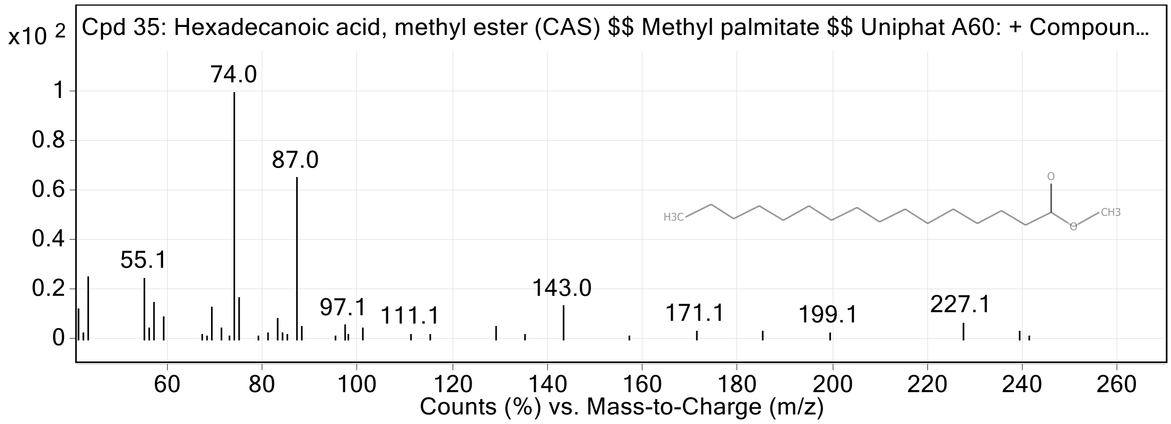

MS Zoomed Spectrum

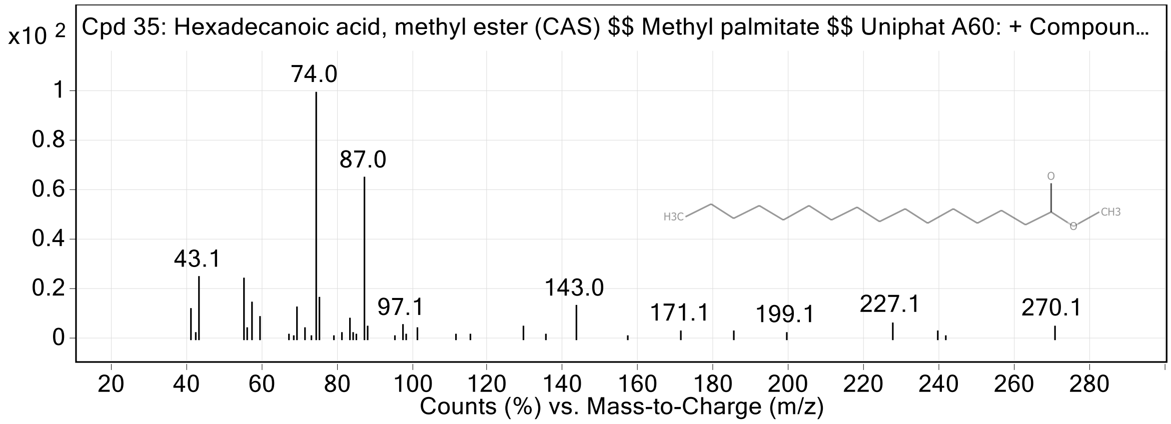

MS Spectrum Peak List

m/z	Abund
41,1	29284,8
43,1	56971,9
55,1	56291,7
57,1	35100,2
59,1	21791,8
69,1	30364,3
74	221358,3
75	39542,9
87	145320,6
143	31298,1

Library Spectrum

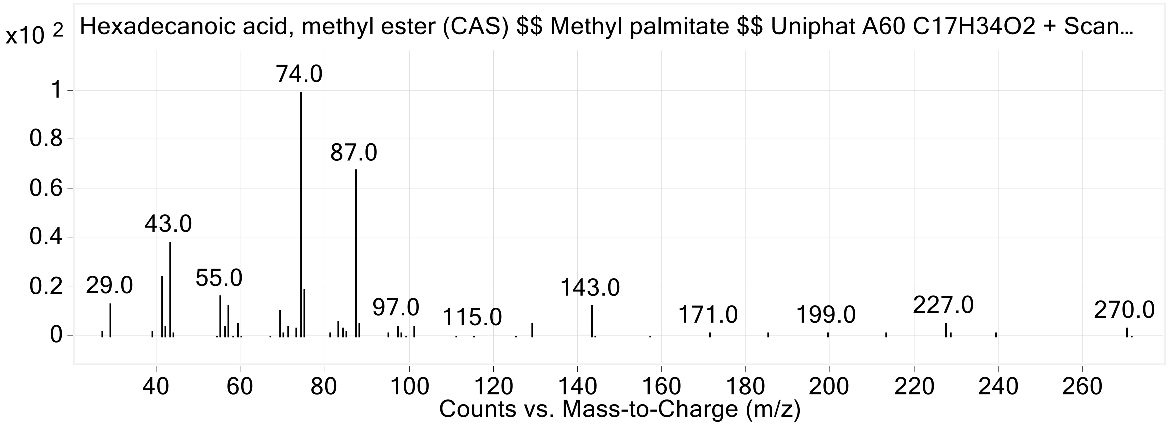

Difference Spectrum

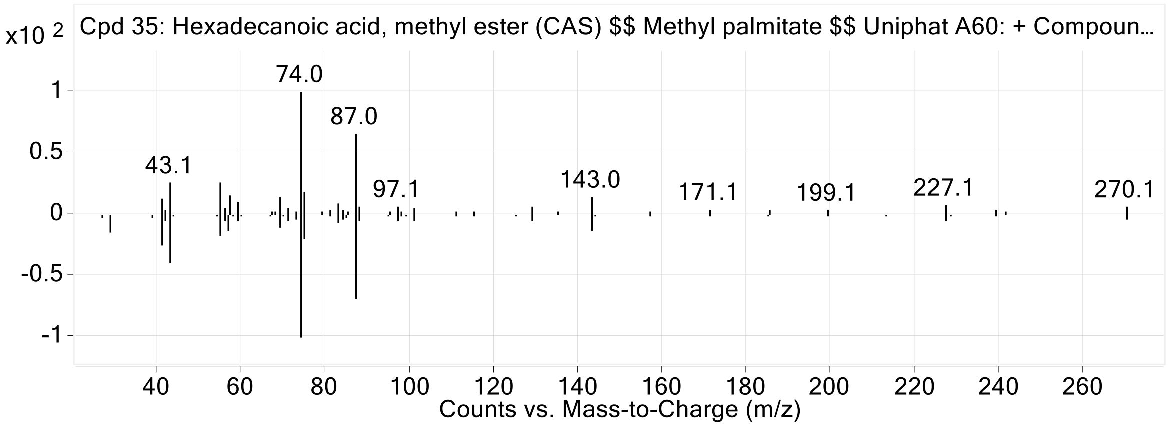

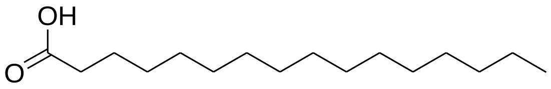

(L)

Compound Label	Name	*m/z*	RT	Algorithm

Cpd 39: n-Hexadecanoic acid $$ Hexadecanoic acid $$ n-Hexadecoic acid $$ Palmitic acid	**n-Hexadecanoic acid $$ Hexadecanoic acid $$ n-Hexadecoic acid $$ Palmitic acid**	73	43,36	Find by Chromatogram Deconvolution

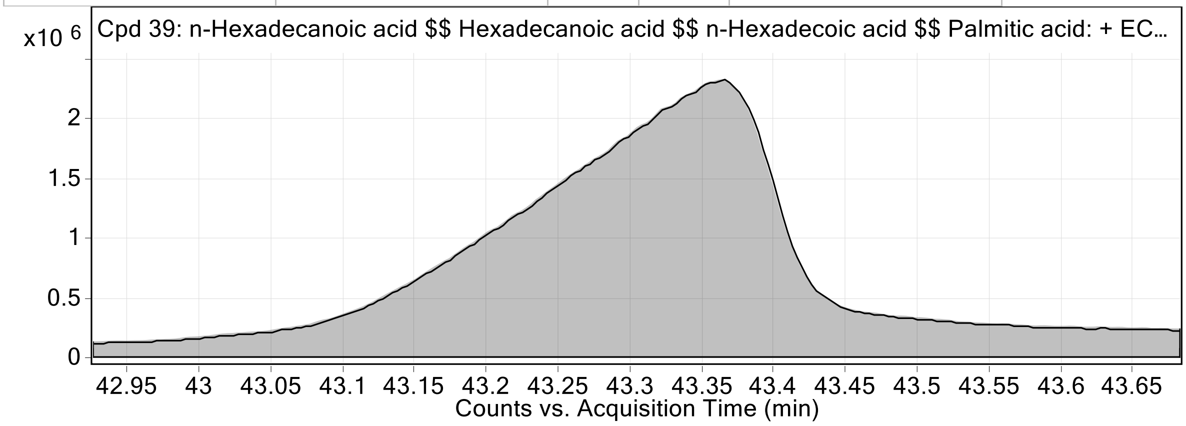

MS Spectrum

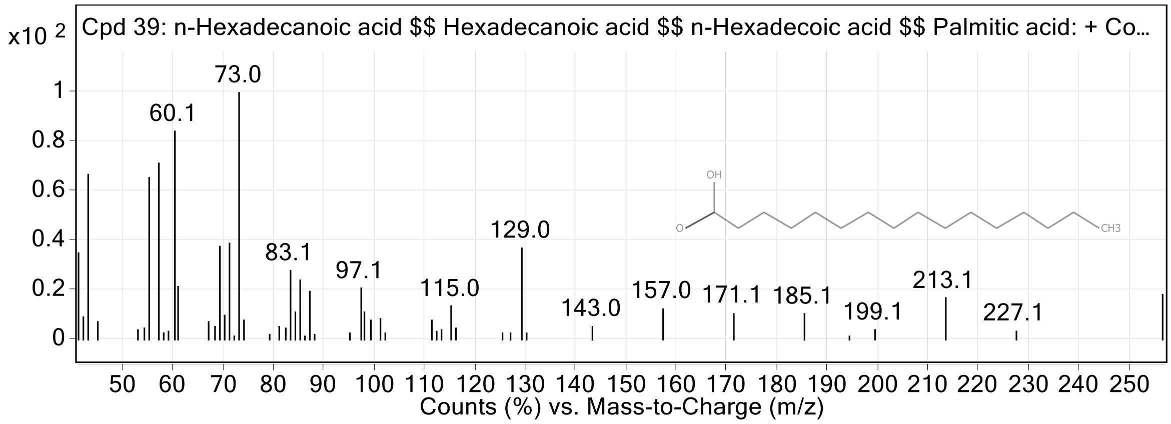

MS Zoomed Spectrum

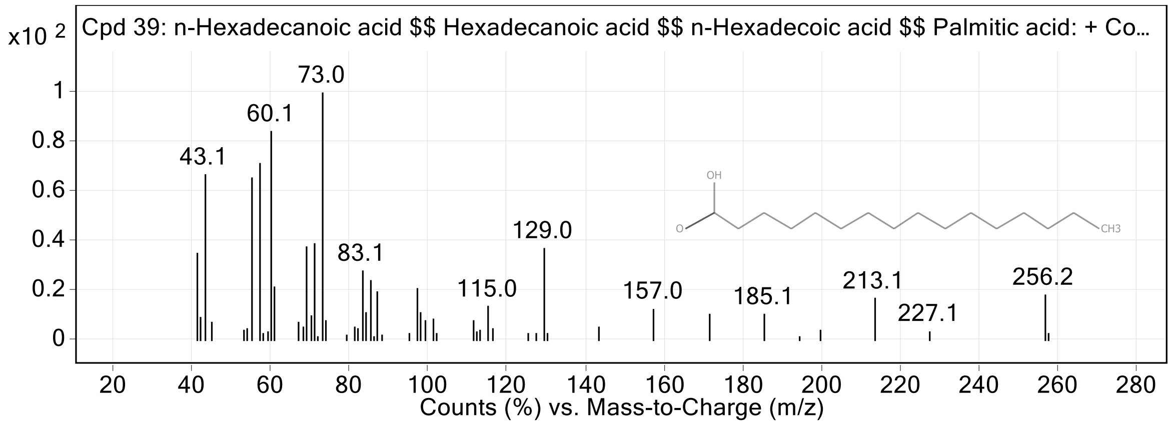

MS Spectrum Peak List

m/z	Abund
41,1	83991,3
43,1	159560,1
55,1	156711
57,1	169956,4
60,1	200766
69,1	90831,3
71,1	93169,7
73	237672,3
83,1	67764,7
129	89315,9

Library Spectrum

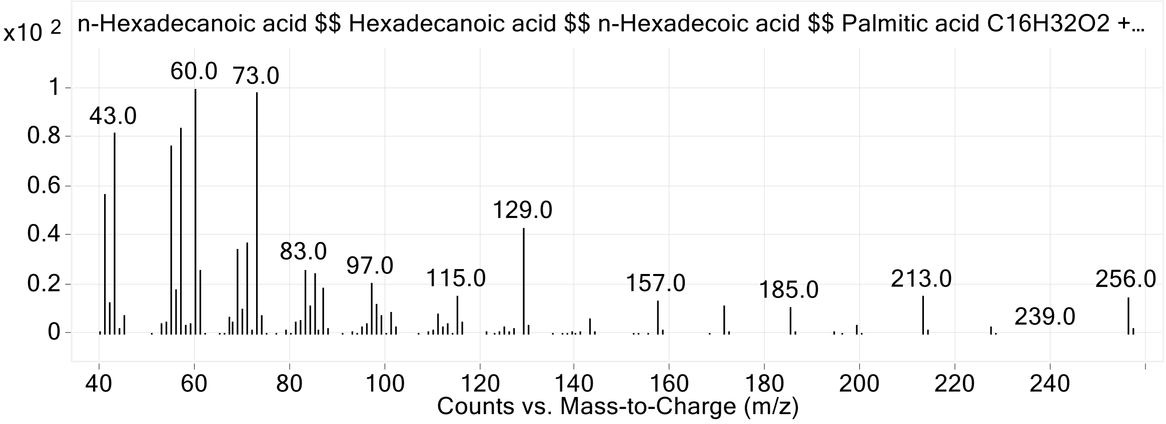

Difference Spectrum

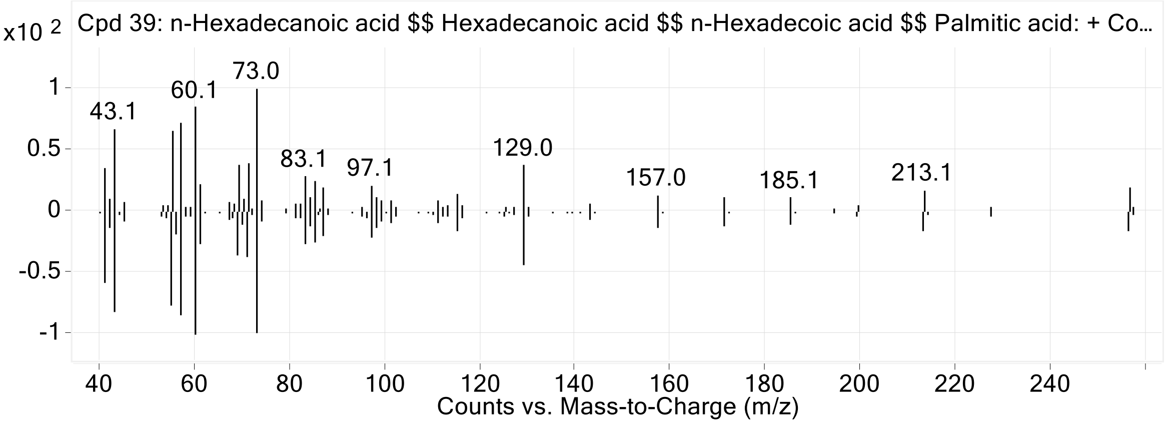

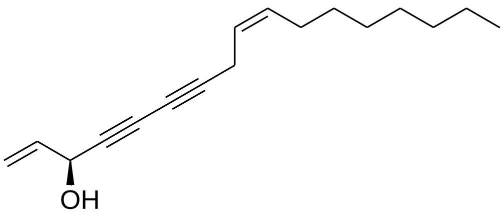

(M)

Compound Label	Name	*m/z*	RT	Algorithm
Cpd 44: Falcarinol $$ (Z)-(-)-1,9-heptadecadiene-4,6-diyne-3-ol	**Falcarinol $$ (Z)-(-)-1,9-heptadecadiene-4,6-diyne-3-ol**	55,1	45,8	Find by Chromatogram Deconvolution

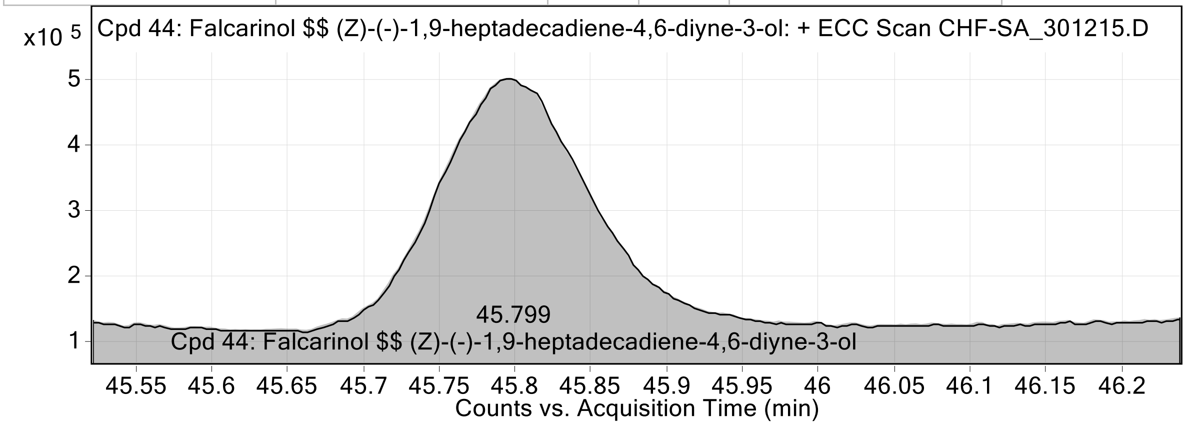

MS Spectrum

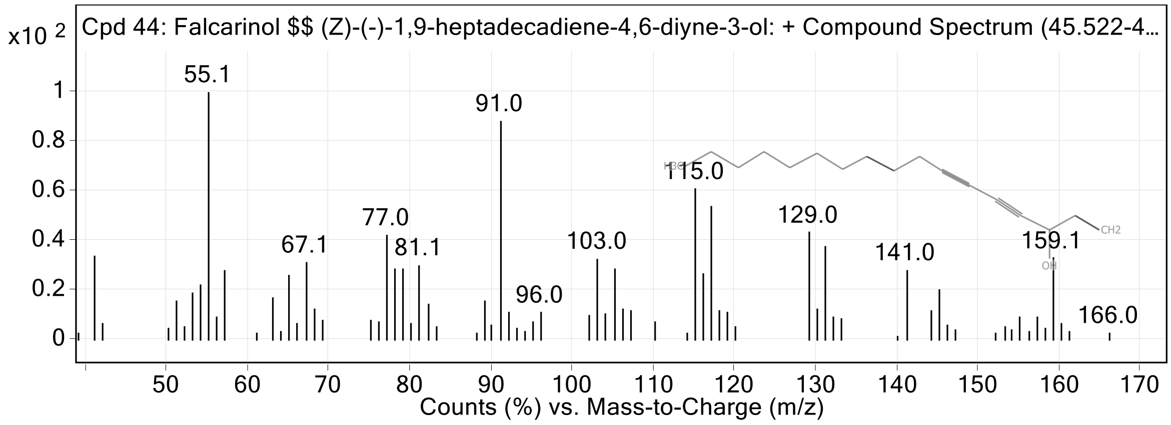

MS Zoomed Spectrum

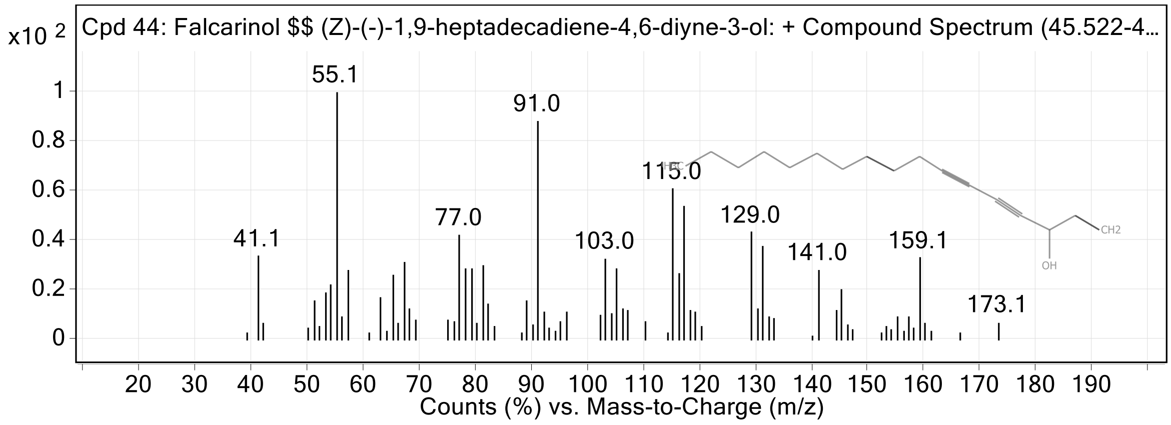

MS Spectrum Peak List

m/z	Abund
41,1	11026
55,1	32352
77	13777
m/z	Abund
91	28589
103	10722
115	19781
117	17446
129	14109
131	12332
159,1	10776

Library Spectrum

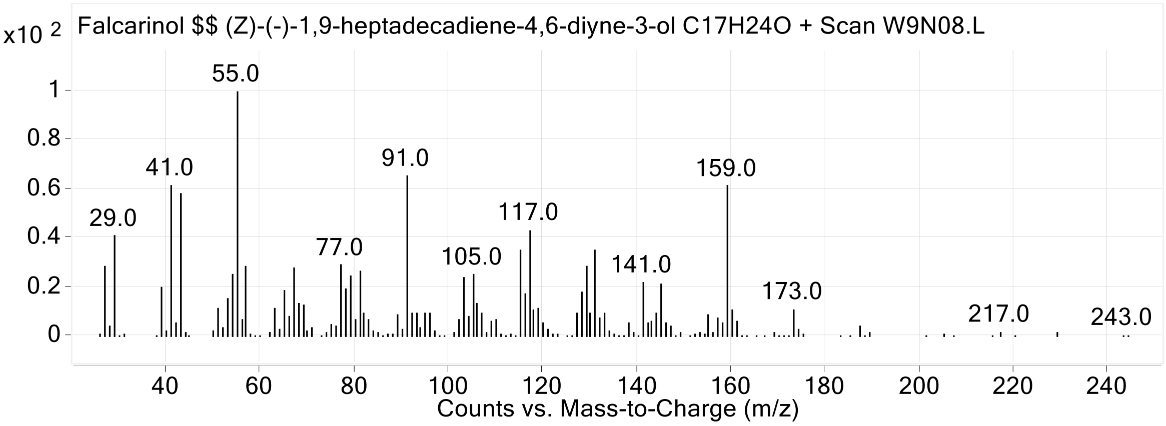

Difference Spectrum

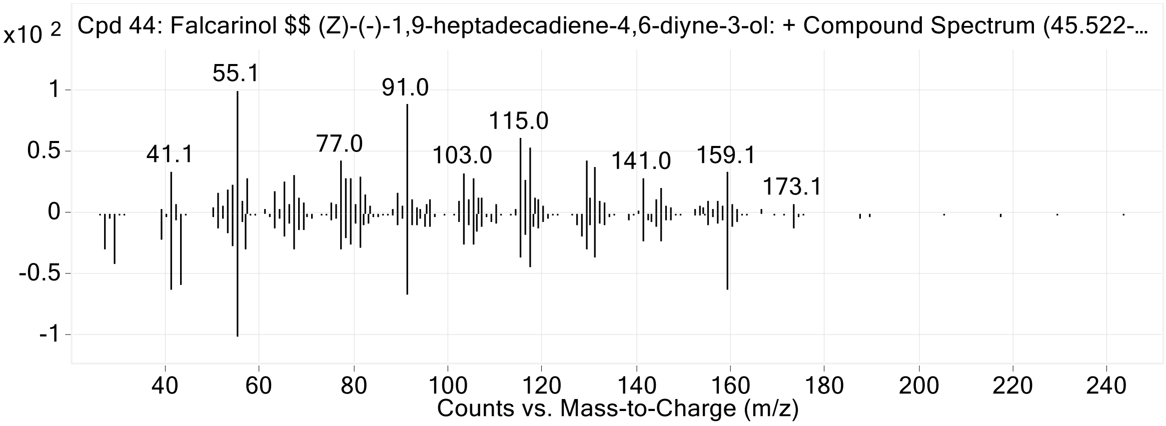

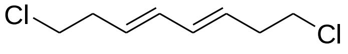

(N)

Compound Label	Name	*m/z*	RT	Algorithm

Cpd 52: 3,5-Octadiene, 1,8-dichloro- $$ (3E,5E)-1,8-Dichloro-3,5-octadiene #	**3,5-Octadiene, 1,8-dichloro- $$ (3E,5E)-1,8-Dichloro-3,5-octadiene #**	55,1	55,74	Find by Chromatogram Deconvolution

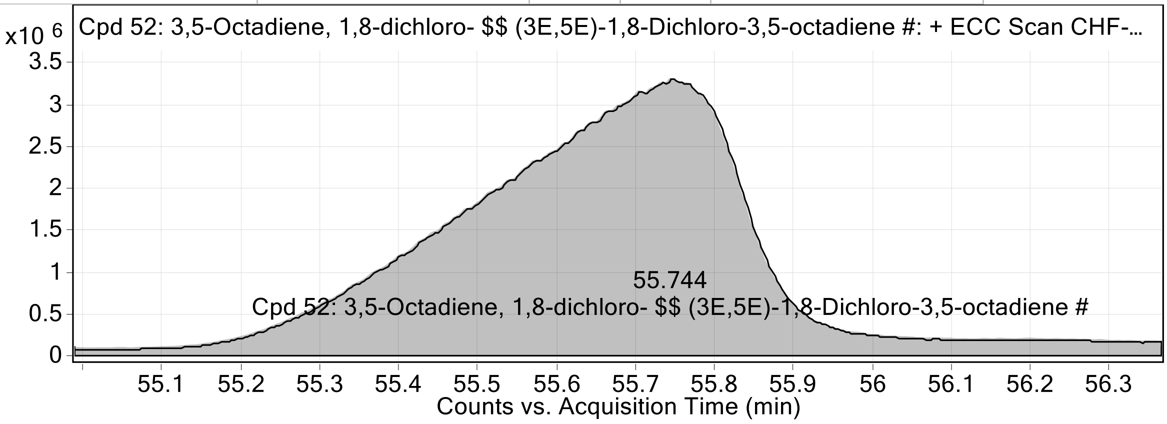

MS Spectrum

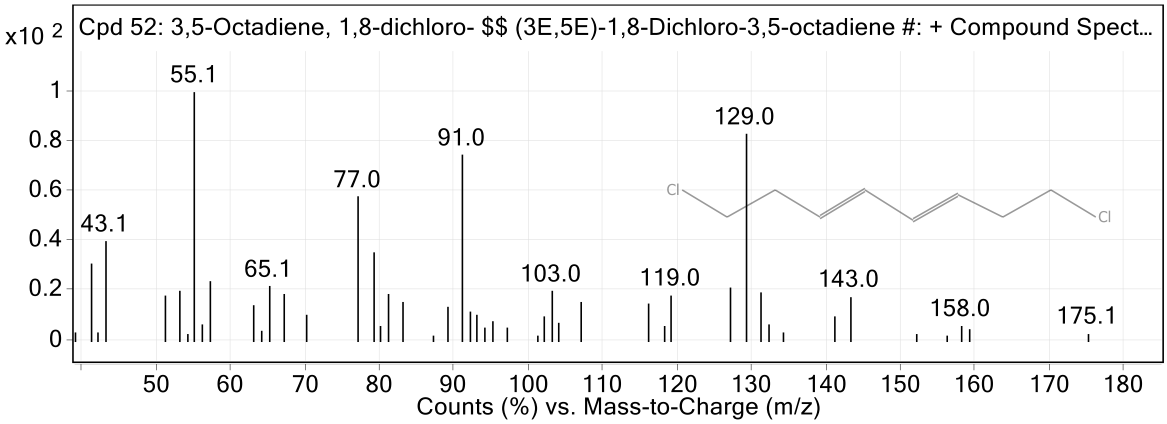

MS Zoomed Spectrum

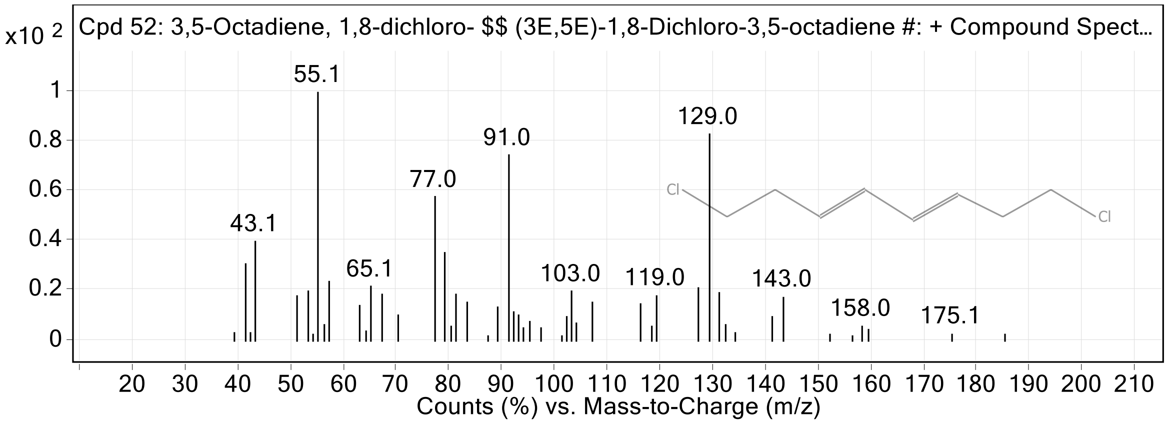

MS Spectrum Peak List

m/z	Abund
41,1	114972,6
43,1	147918
55,1	368895,9
57,1	89602,6
65,1	80822,7
77	213445,8
79,1	132115,1
91	275413,8
127	80105,7
129	306579,1

Library Spectrum

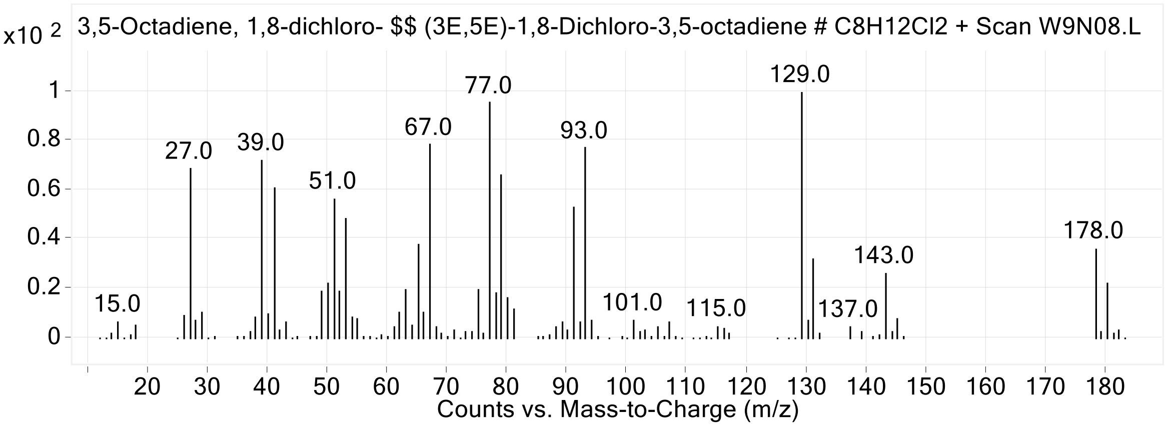

Difference Spectrum

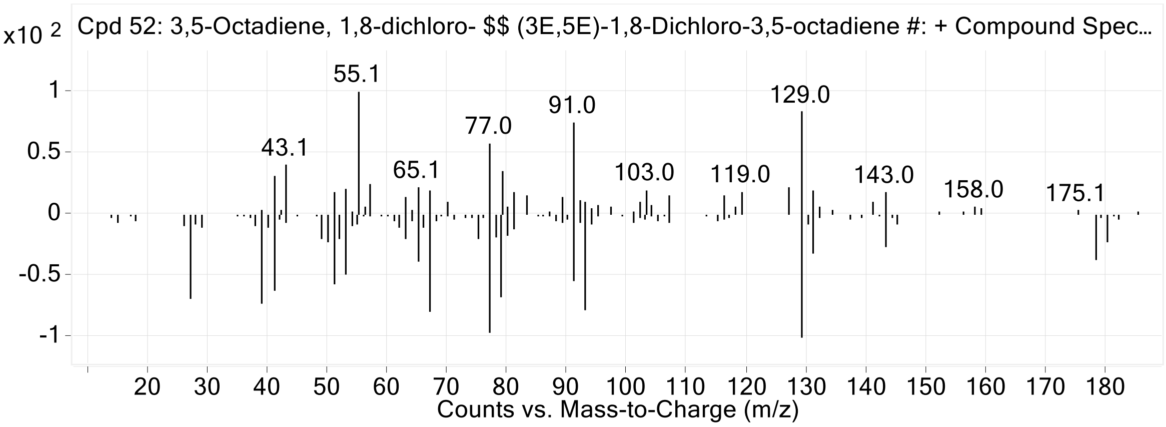

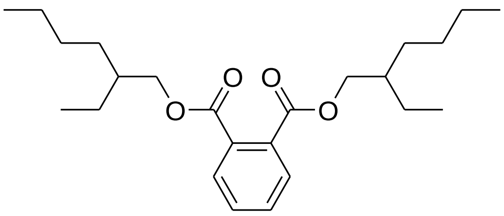

(O)

Compound Label	Name	*m/z*	RT	Algorithm
Cpd 65: 1,2-Benzenedicarboxylic acid, bis(2-ethylhexyl) ester (CAS) $$ DEHP $$ DNOP	**1,2-Benzenedicarboxylic acid, bis(2-ethylhexyl) ester (CAS) $$ DEHP $$ DNOP**	149	63,06	Find by Chromatogram Deconvolution

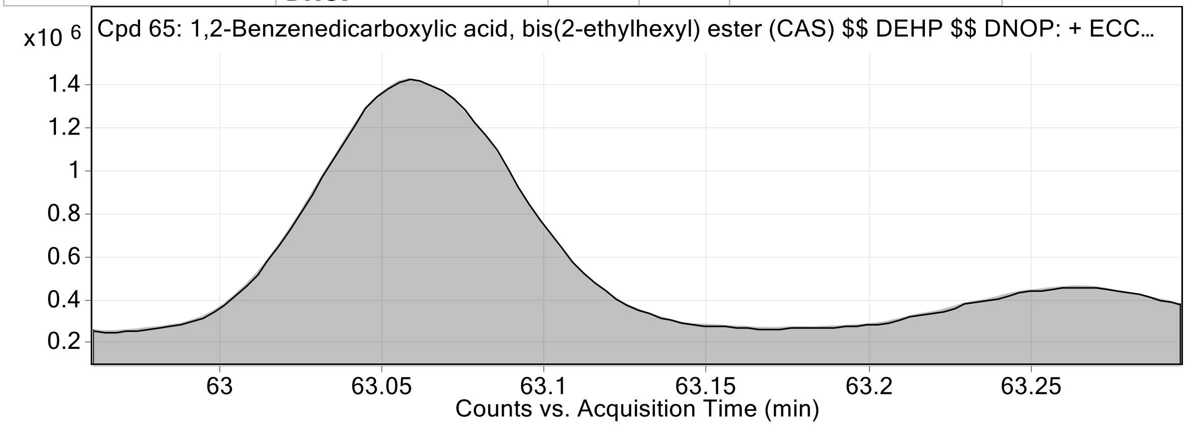

MS Spectrum

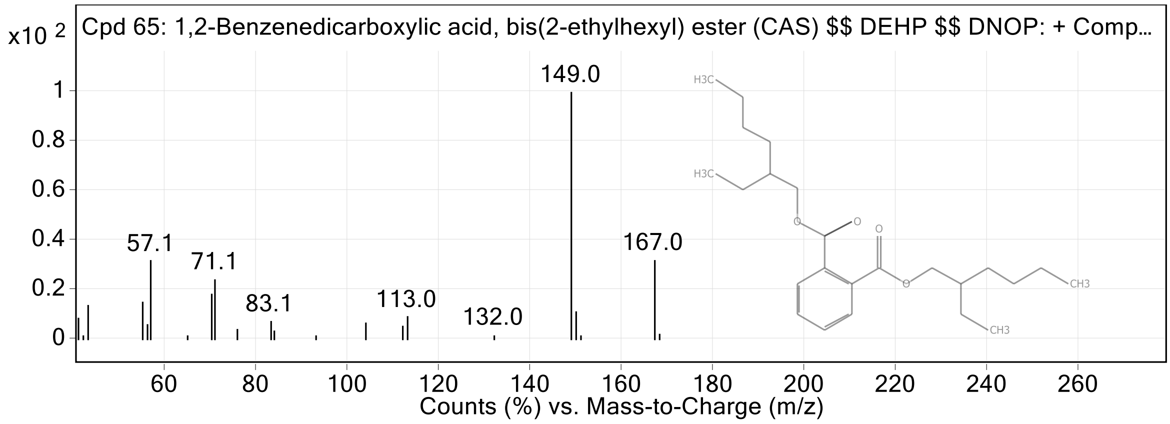

MS Zoomed Spectrum

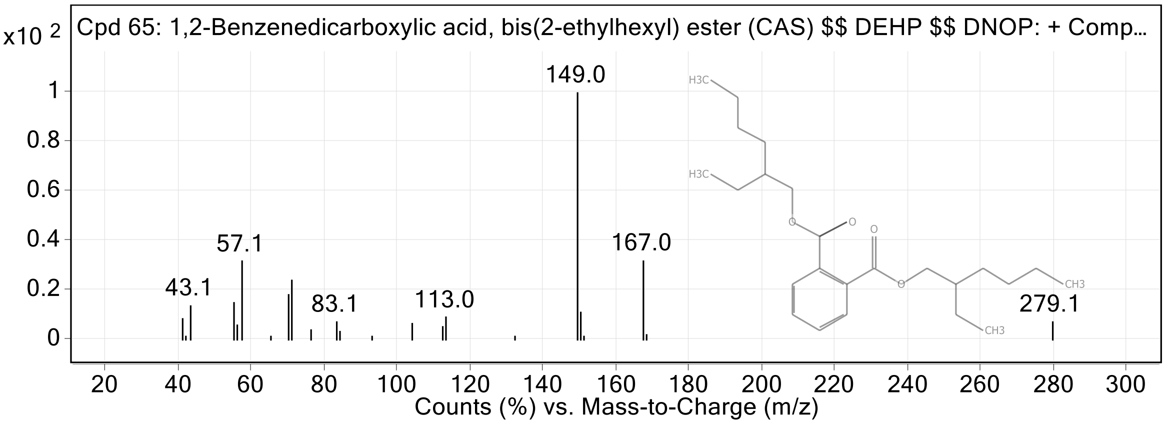

MS Spectrum Peak List

m/z	Abund
41,1	34293,9
43,1	54129,7
55,1	58312
57,1	120270,4
70,1	71334,9
71,1	92917,6
113	36055,1
149	374681,7
150	43686
167	121940,7

Library Spectrum

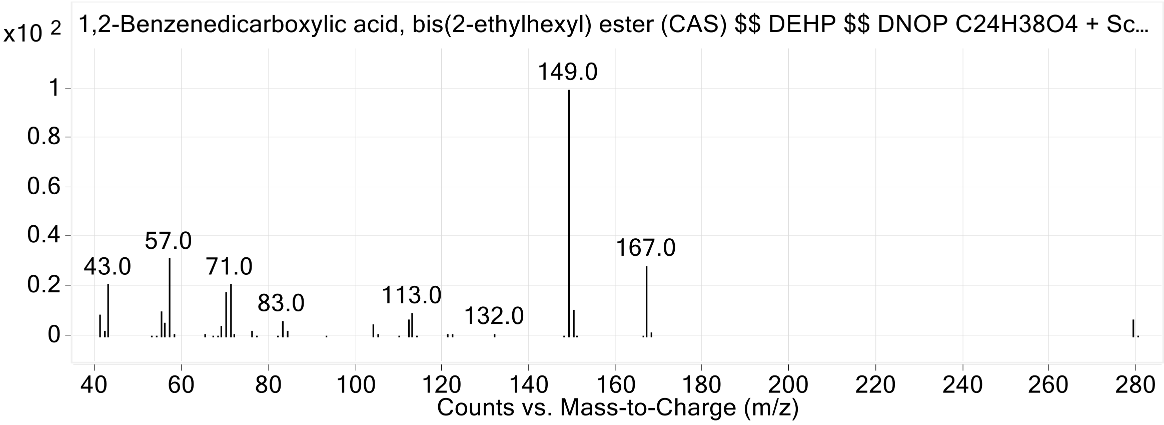

Difference Spectrum

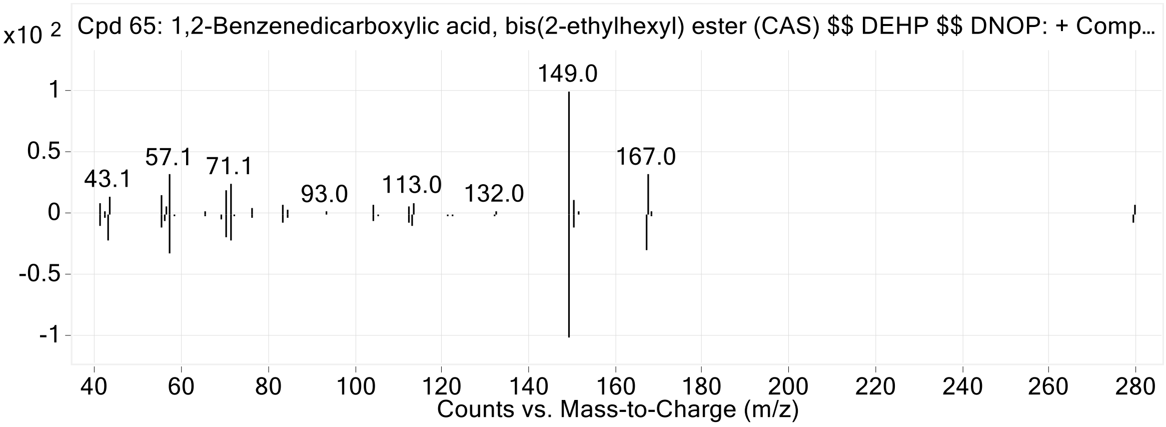
x10 2
Cpd 65: 1,2-Benzenedicarboxylic acid, bis(2-ethylhexyl) ester (CAS) $$ DEHP $$ DNOP: + Comp...
1
0.5
0
-0.5
-1
43.1
57.1
71.1
93.0
113.0
132.0
149.0
167.0
40
60
80
100
120
140
160
180
200
220
240
260
280
Counts vs. Mass-to-Charge (m/z)
