# Supplementary material for: Treating Hyperglycemia From Eryngium caeruleum M. Bieb: In-vitro α-Glucosidase, Antioxidant, in-vivo Antidiabetic and Molecular Docking-Based Approaches
Source: Front Chem. 2020 Nov 26;8:558641. doi: 10.3389/fchem.2020.558641 (PMC7737655; doi:10.3389/fchem.2020.558641)
Supplement: Supplementary file 2 [file Data_Sheet_2.PDF]

Treating hyperglycemia from *Eryngium caeruleum* M. Bieb: *In-vitro* α-glucosidase, antioxidant, *in-vivo* antidiabetic and molecular docking-based approaches

Abdul Sadiq*[1], Umer Rashid[2], Sadiq Ahmad[1], Mohammad Zahoor[3], Farhat Ullah[1], Muhammad Ayaz[1], Muhammad Iftikhar Khan[4] and Zia-Ul Islam[5]

[1]Department of Pharmacy, Faculty of Biological Sciences, University of Malakand, Chakdara, 18000 Dir (L), KP, Pakistan.

[2]Department of Chemistry, COMSATS University Islamabad, Abbottabad Campus, Abbottabad 22060, Pakistan

[3]Department of Chemistry, University of Malakand, Chakdara, 18000 Dir (L), KP, Pakistan.

[4]Department of Pharmacy, COMSATS University Islamabad, Abbottabad Campus, Abbottabad 22060, Pakistan

[5]Department of Biotechnology, Abdul Wali Khan University Mardan, KP, Pakistan

Corresponding author (*): Dr. Abdul Sadiq, Associate Professor, Department of Pharmacy, Faculty of Biological Sciences, University of Malakand, Chakdara 18000, Dir (L), KP, Pakistan. Email: sadiquom@yahoo.com; Contact No. +92(0)301-2297 102

Supporting Information-II

(Bioactive compounds)

<u>**GC-MS spectra/details of the compounds**</u>

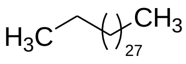

Triacontane

Compound Label	Name	*m/z*	RT	Algorithm
Cpd 66: Triacontane (CAS) $$ n-Triacontane $$ n - triacontane	**Triacontane (CAS) $$ n-Triacontane $$ n - triacontane**	57,1	63,26	Find by Chromatogram Deconvolution

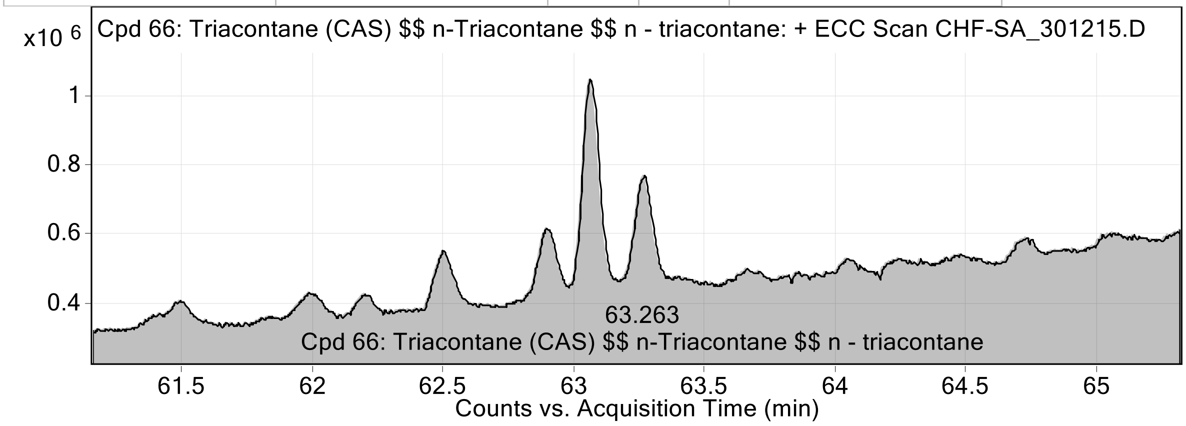

MS Spectrum

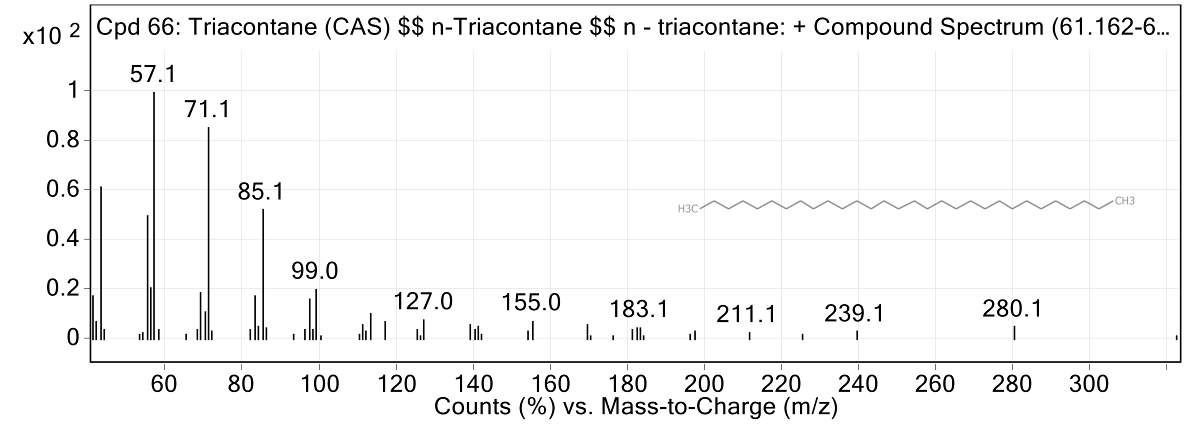

MS Zoomed Spectrum

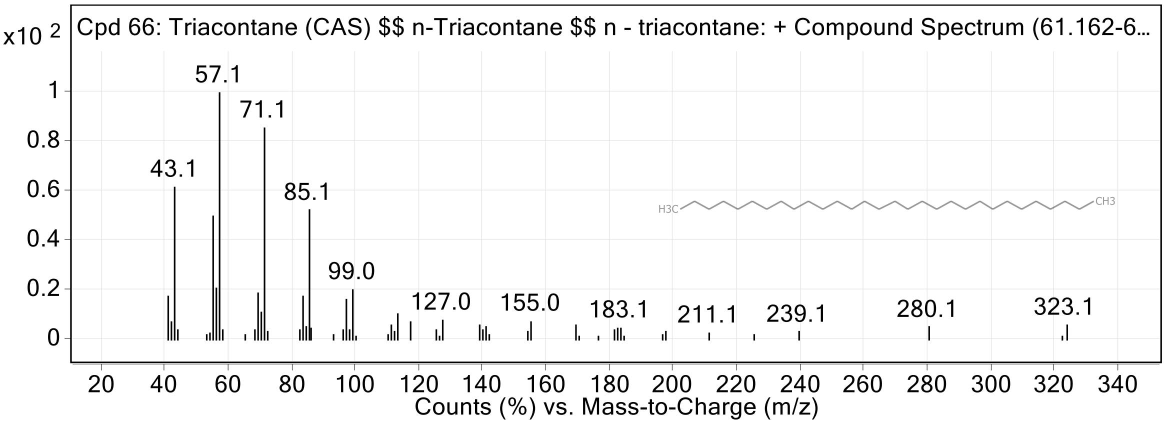

MS Spectrum Peak List

m/z	Abund
41,1	10892
43,1	37206
55,1	30142
56,1	12737
57,1	59929
69,1	11574
71,1	51468
83,1	10932
85,1	31629
99	12534

Library Spectrum

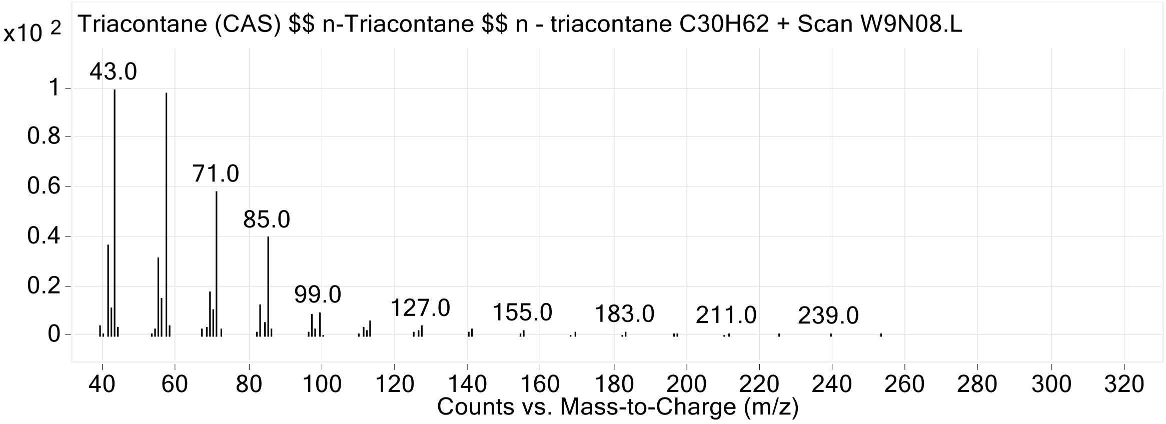

Difference Spectrum

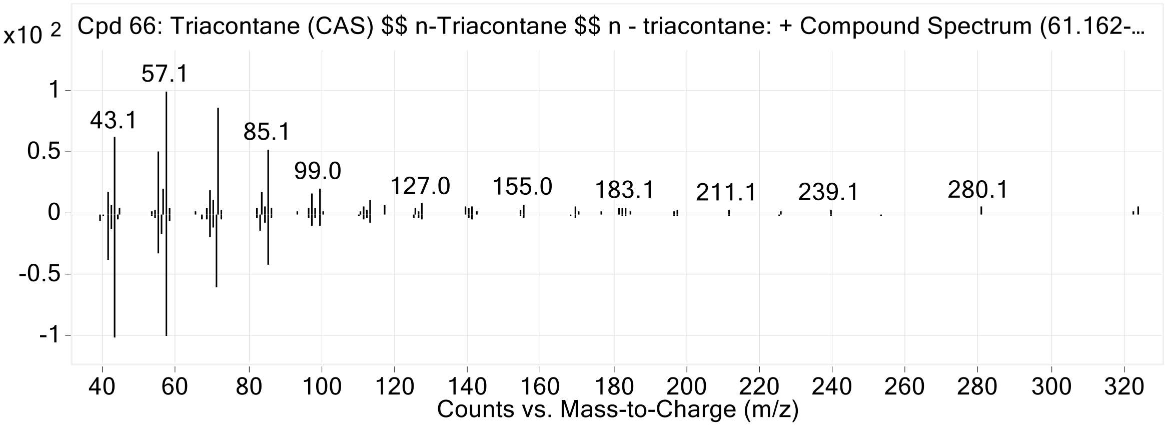

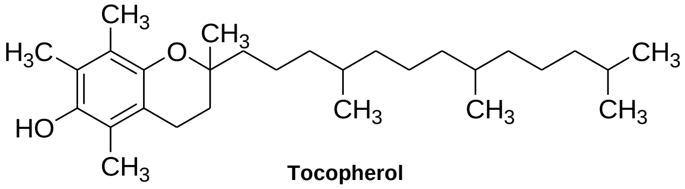

Tocopherol

Compound Label	Name	*m/z*	RT	Algorithm
Cpd 110: Vitamin e $$ .alpha.-Tocopherol $$ Vi-E $$ Esorb $$ Evion $$ Endo E $$ Etavit	**Vitamin e $$ .alpha.-Tocopherol $$ Vi-E $$ Esorb $$ Evion $$ Endo E $$ Etavit**	165	69,78	Find by Chromatogram Deconvolution

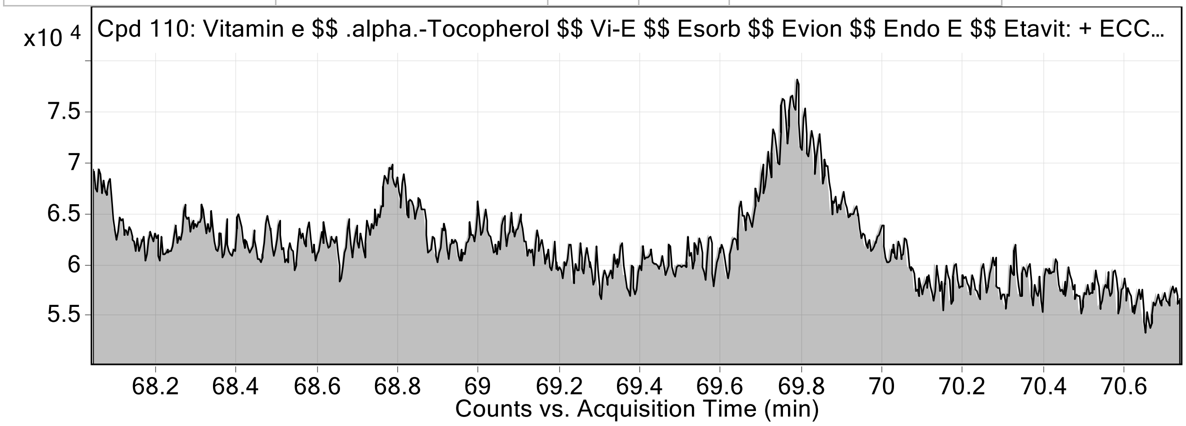

MS Spectrum

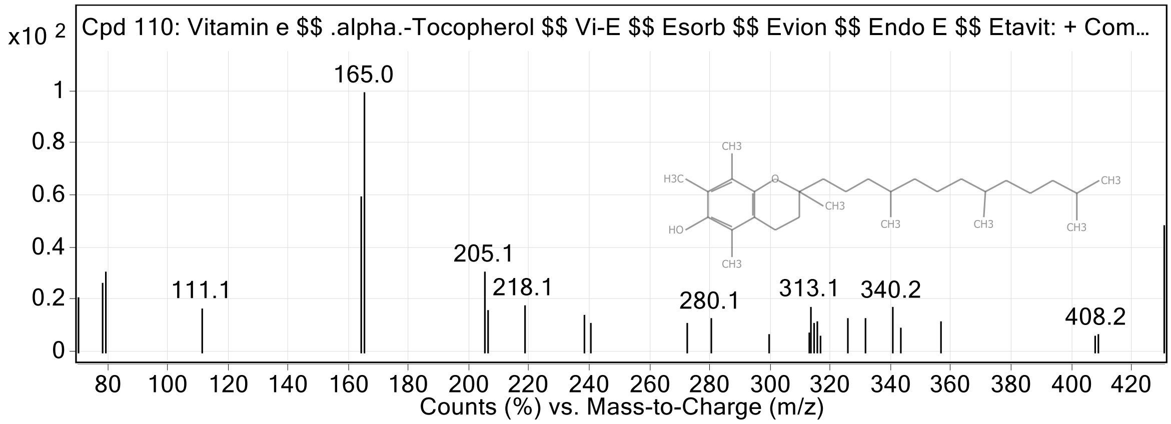

MS Zoomed Spectrum

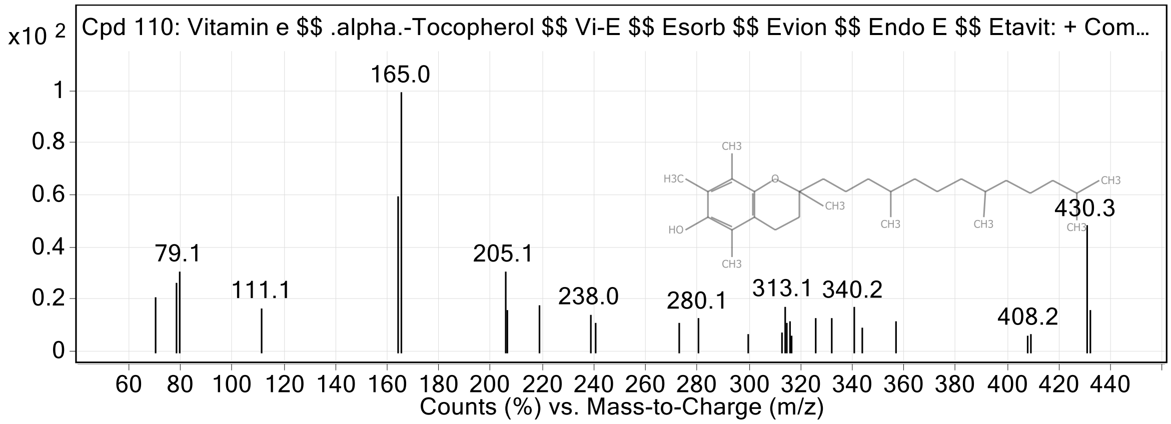

MS Spectrum Peak List

m/z	Abund
70,1	1737
78	2165,5
79,1	2521,9
164	4798,3
165	8035,8
205,1	2516,6
218,1	1478,3
313,1	1448
340,2	1401,5
430,3	3914,6

Library Spectrum

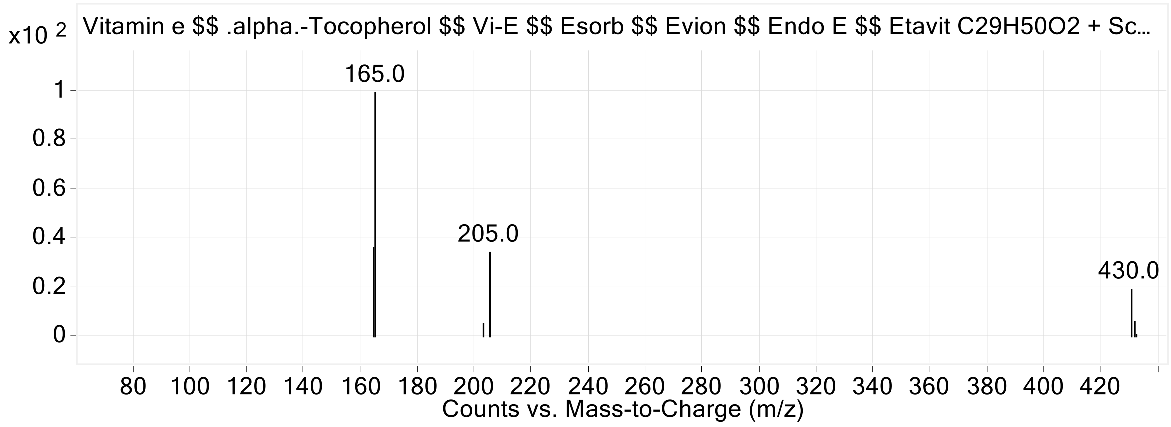

Difference Spectrum

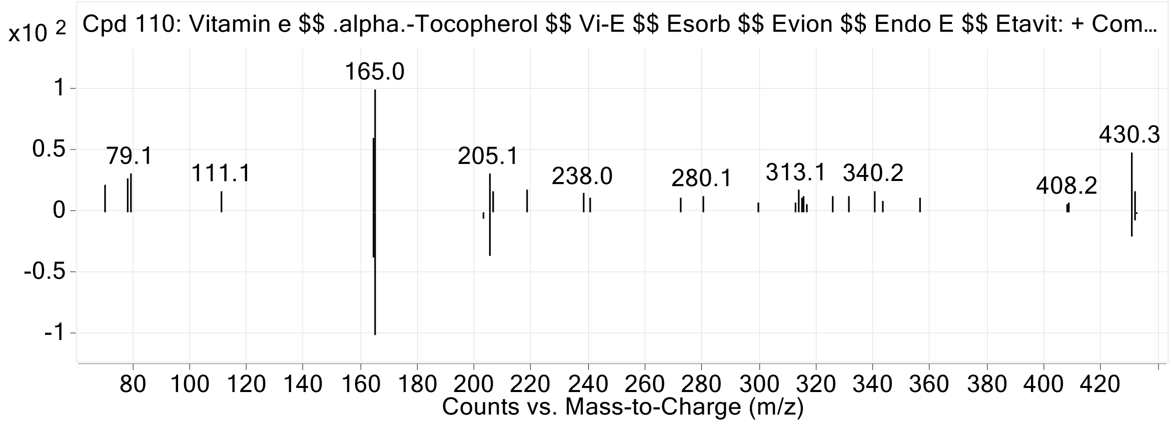

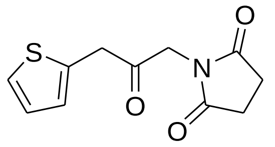

2,5-Pyrrolidione, N-[(thienyl)acetyloxy]

Compound Label	Name	*m/z*	RT	Algorithm
Cpd 59: 2,5-Pyrrolidione, N-[2-(thienyl)acetyloxy]-	**2,5-Pyrrolidione, N-[2-(thienyl)acetyloxy]-**	55,1	60,13	Find by Chromatogram Deconvolution

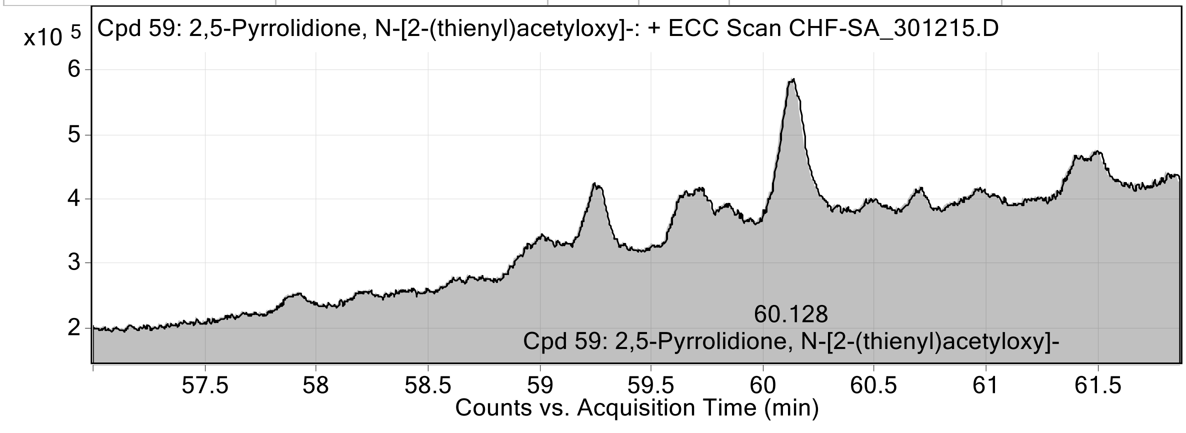

MS Spectrum

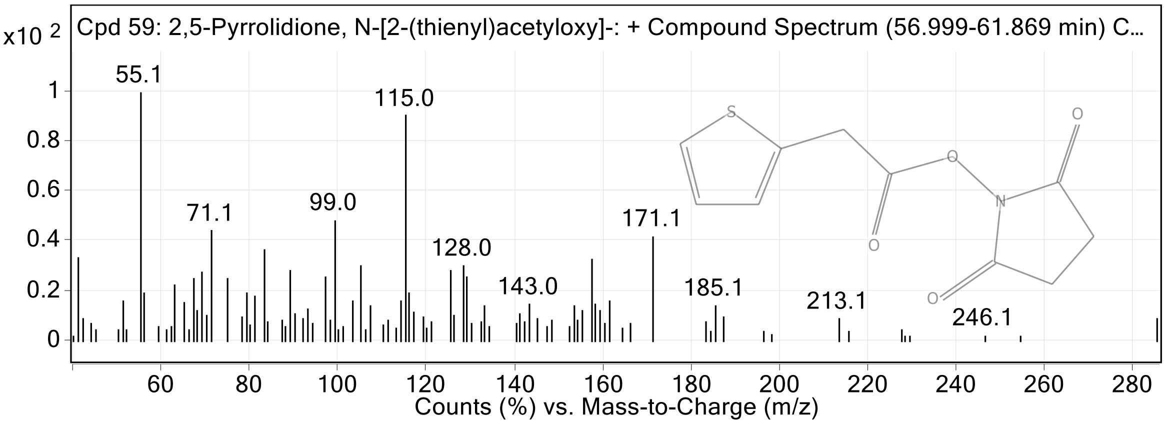

MS Zoomed Spectrum

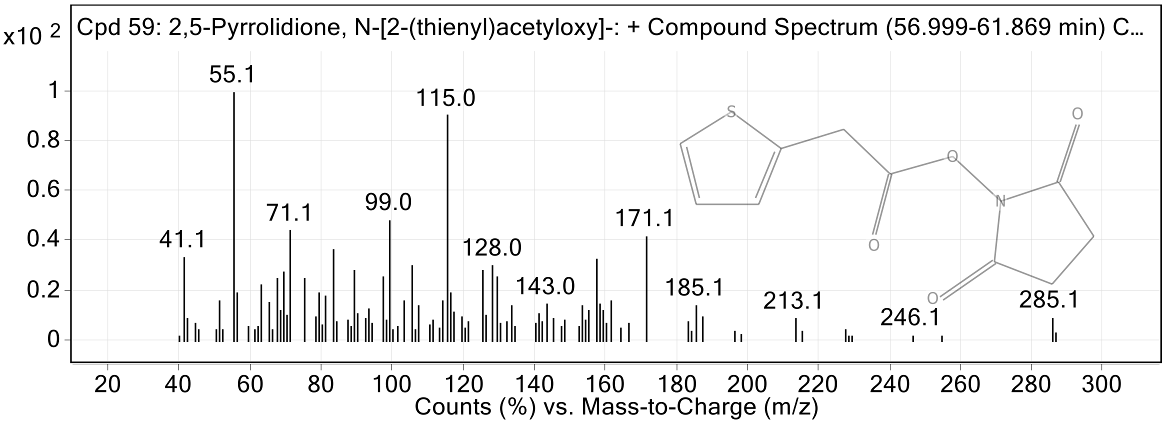

MS Spectrum Peak List

m/z	Abund
41,1	7298,5
55,1	21500
71,1	9583,6
83,1	7919,3
99	10504
105	6645,8
115	19472
128	6584
157	7194,6
171,1	9118,9

Library Spectrum

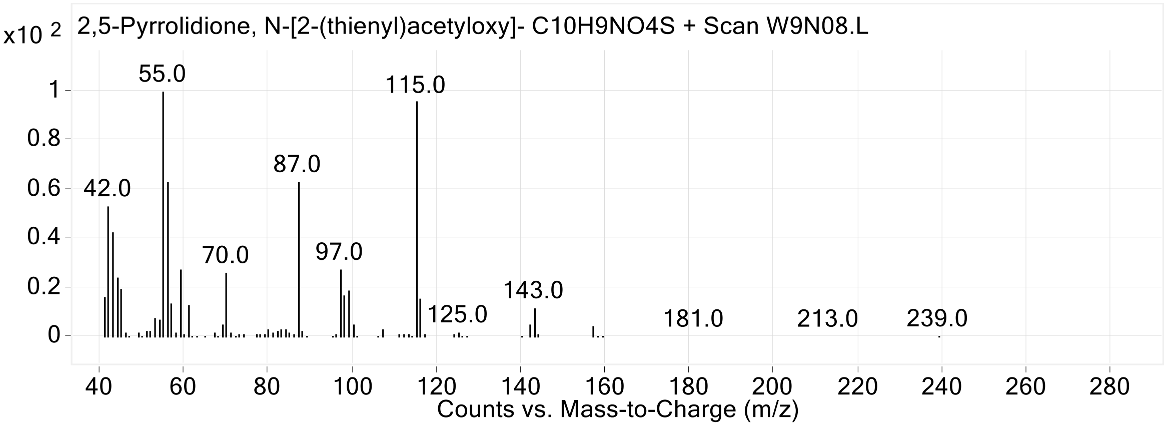

Difference Spectrum

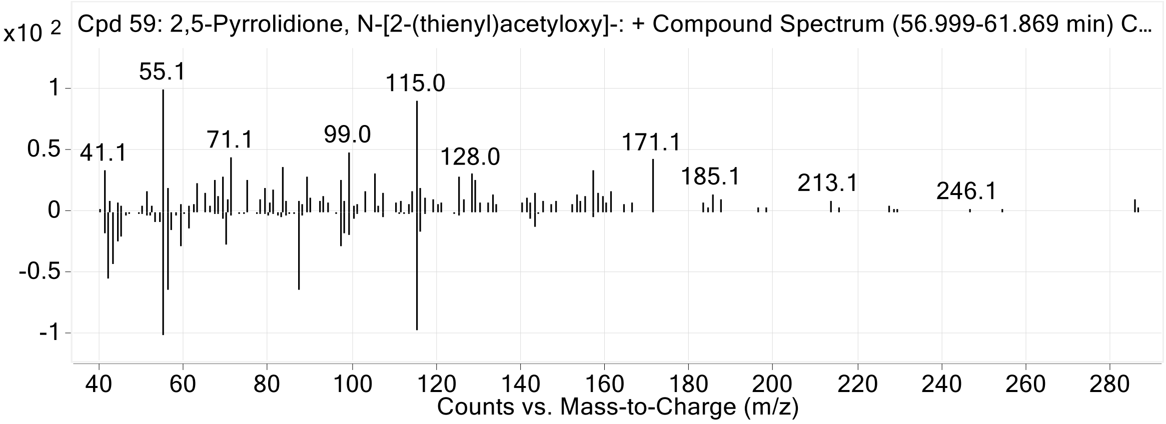

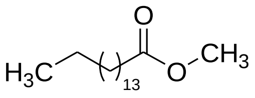

Methyl palmitate

This is a repeated compound. For details please see the supporting information of Compound **(K)** in Figure 2.

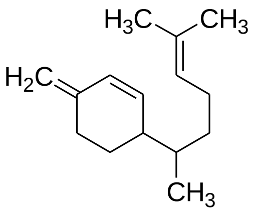

Cyclohexene, 3-(1,5-dimethyl-4-hexenyl)-6-methylene

This is a repeated compound. For details please see the supporting information of Compound **(C)** in Figure 2.

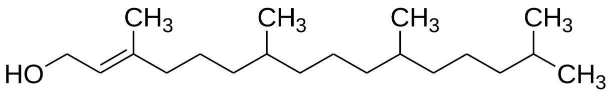

Phytol

This is a repeated compound. For details please see the supporting information of Compound **(J)** in Figure 2.

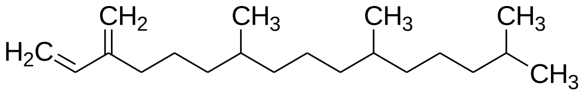

(I)-Neophytadiene

This is a repeated compound. For details please see the supporting information of Compound **(I)** in Figure 2.

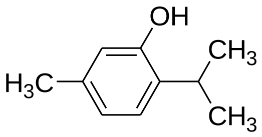

Thymol

This is a repeated compound. For details please see the supporting information of Compound **(B)** in Figure 2.

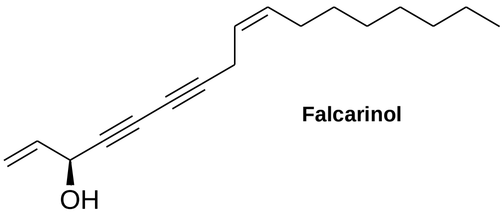

Falcarinol

This is a repeated compound. For details please see the supporting information of Compound **(M)** in Figure 2.

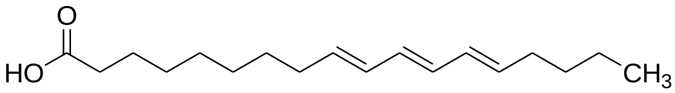

Linolenic acid

**Compound Label**	**Name**	***m/z***	**RT**	**Algorithm**
Cpd 49: 9,12,15-Octadecatrienoic acid, (Z,Z,Z)- $$ Linolenic acid $$ Industrene 120	**9,12,15-Octadecatrienoic acid, (Z,Z,Z)- $$ Linolenic acid $$ Industrene 120**	55,1	51,53	Find by Chromatogram Deconvolution

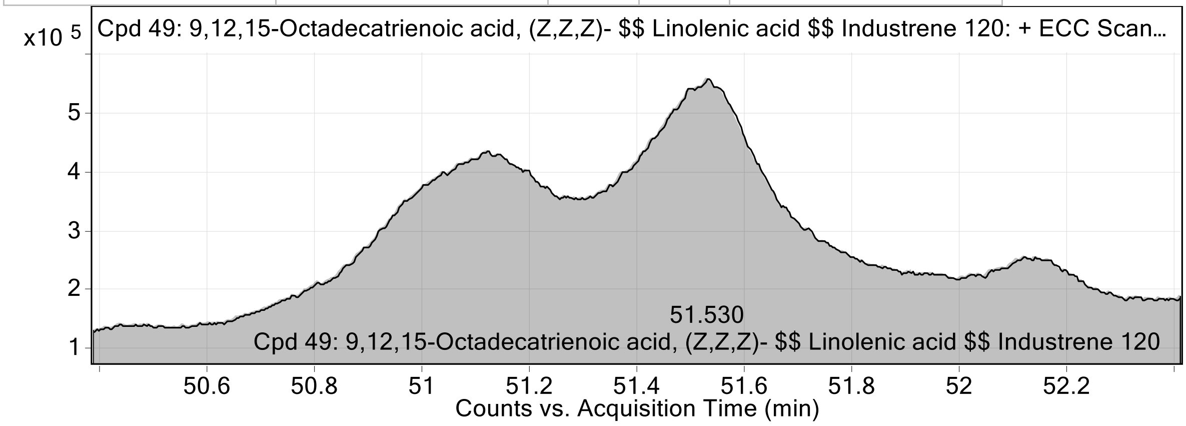

MS Spectrum

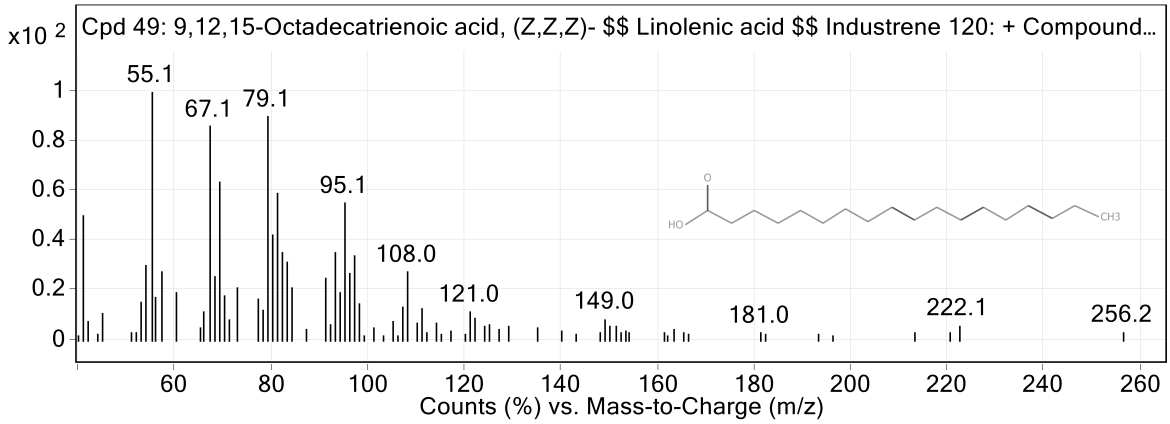

MS Zoomed Spectrum

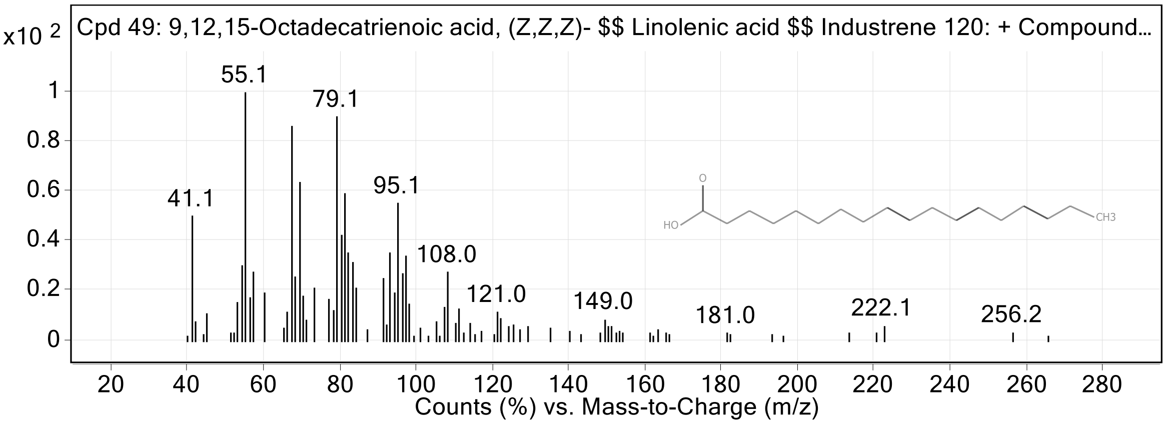

MS Spectrum Peak List

m/z	Abund
41,1	15854
55,1	31558
67,1	27172
69,1	20050
79,1	28464
80	13457
81,1	18698
82,1	11214
93	11237
95,1	17501

Library Spectrum

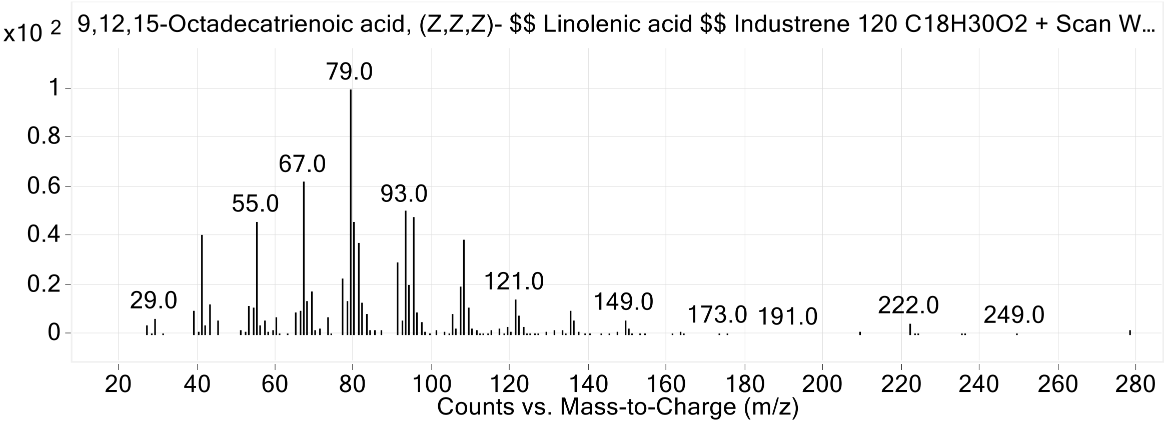

Difference Spectrum

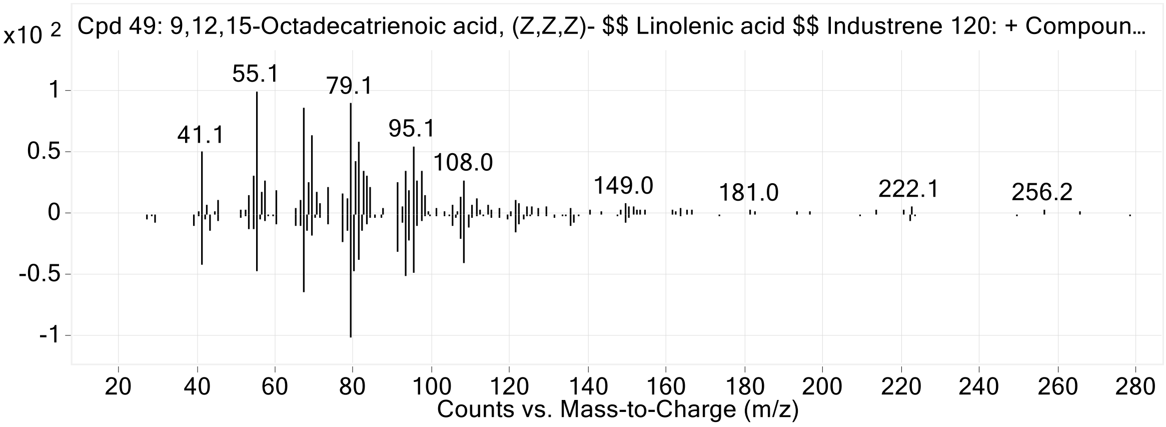

Cpd 49: 9,12,15-Octadecatrienoic acid, (Z,Z,Z)- $$ Linolenic acid $$ Industrene 120: + Compoun...
x10 2
1
0.5
0
-0.5
-1
41.1
55.1
79.1
95.1
108.0
149.0
181.0
222.1
256.2
20
40
60
80
100
120
140
160
180
200
220
240
260
280
Counts vs. Mass-to-Charge (m/z)
