# Supplementary figures and images for: Treating Hyperglycemia From Eryngium caeruleum M. Bieb: In-vitro α-Glucosidase, Antioxidant, in-vivo Antidiabetic and Molecular Docking-Based Approaches
Source: Front Chem. 2020 Nov 26;8:558641. doi: 10.3389/fchem.2020.558641 (PMC7737655; doi:10.3389/fchem.2020.558641)

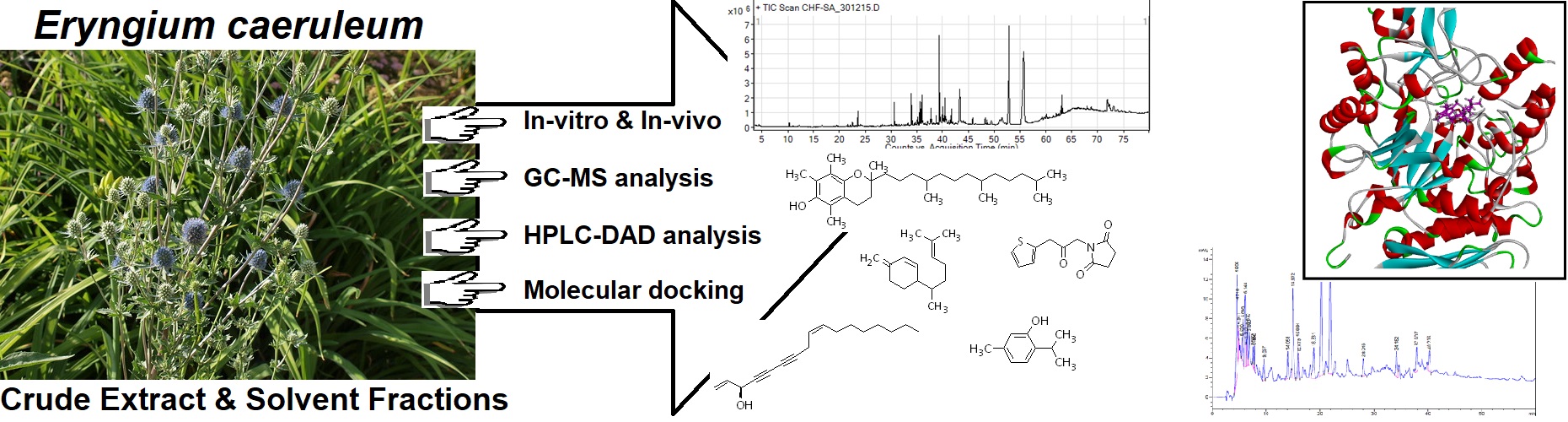

Supplement: Supplementary file 3 [file Image_1.JPEG]
